# Spatially explicit models of seasonal habitat for greater sage‐grouse at broad spatial scales: Informing areas for management in Nevada and northeastern California

**DOI:** 10.1002/ece3.5842

**Published:** 2019-11-25

**Authors:** Peter S. Coates, Brianne E. Brussee, Mark A. Ricca, John P. Severson, Michael L. Casazza, Kit Benjamin Gustafson, Shawn P. Espinosa, Scott C. Gardner, David J. Delehanty

**Affiliations:** ^1^ Western Ecological Research Center U.S. Geological Survey Dixon CA USA; ^2^ Nevada Department of Wildlife Reno NV USA; ^3^ California Department of Fish and Wildlife Sacramento CA USA; ^4^ Department of Biological Sciences Idaho State University Pocatello ID USA

**Keywords:** *Centrocercus urophasianus*, Great Basin, resource selection function, sage‐grouse, seasonal mapping, spatiotemporal variation

## Abstract

Defining boundaries of species' habitat across broad spatial scales is often necessary for management decisions, and yet challenging for species that demonstrate differential variation in seasonal habitat use. Spatially explicit indices that incorporate temporal shifts in selection can help overcome such challenges, especially for species of high conservation concern. Greater sage‐grouse *Centrocercus urophasianus* (hereafter, sage‐grouse), a sagebrush obligate species inhabiting the American West, represents an important case study because sage‐grouse exhibit seasonal habitat patterns, populations are declining in most portions of their range and are central to contemporary national land use policies. Here, we modeled spatiotemporal selection patterns for telemetered sage‐grouse across multiple study sites (1,084 sage‐grouse; 30,690 locations) in the Great Basin. We developed broad‐scale spatially explicit habitat indices that elucidated space use patterns (spring, summer/fall, and winter) and accounted for regional climatic variation using previously published hydrographic boundaries. We then evaluated differences in selection/avoidance of each habitat characteristic between seasons and hydrographic regions. Most notably, sage‐grouse consistently selected areas dominated by sagebrush with few or no conifers but varied in type of sagebrush selected by season and region. Spatiotemporal variation was most apparent based on availability of water resources and herbaceous cover, where sage‐grouse strongly selected upland natural springs in xeric regions but selected larger wet meadows in mesic regions. Additionally, during the breeding period in spring, herbaceous cover was selected strongly in the mesic regions. Lastly, we expanded upon an existing joint–index framework by combining seasonal habitat indices with a probabilistic index of sage‐grouse abundance and space use to produce habitat maps useful for sage‐grouse management. These products can serve as conservation planning tools that help predict expected benefits of restoration activities, while highlighting areas most critical to sustaining sage‐grouse populations. Our joint–index framework can be applied to other species that exhibit seasonal shifts in habitat requirements to help better guide conservation actions.

## Graphical abstract

Sage‐grouse habitat and populations are declining across their range, which has put this species at the center of national land use policies in western North America. Our study provides valuable information of seasonally important areas for sage‐grouse populations for restoration and/or protection, as well as spatially explicit indices that can be integrated into quantitative planning tools to aid decisions related to sage‐grouse population restoration. We developed these spatially explicit products for populations within Nevada and California, which comprise the majority of sage‐grouse within the Great Basin.

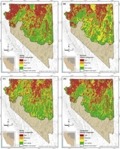

## 1 INTRODUCTION

Habitat selection is a key behavior that strongly affects species distribution and persistence (Manly, McDonald, Thomas, McDonald, & Erickson, 2002). Quantifying habitat attributes allows formal assessments of positive and negative relationships between species and their environment, an endeavor that has been facilitated by the availability of fine‐scale high‐resolution spatial data within geographic information systems (GIS) and multidimensional resource selection modeling. However, temporal and spatial variation in environmental conditions affect prevailing habitat attributes, which may alter the functional response of animals to habitat characteristics and make generalizations difficult (Osborne & Suárez‐Seoane, 2002). Also, a species' life history needs vary temporally across its annual cycle, influencing habitat selection, which should be taken into account to better inform management decisions (Schooley, 1994). A central goal of applied ecology is to develop quantitative tools for managers that account for such variation in habitat selection patterns. Identifying seasonal habitat quality allows managers to specifically improve conditions for important life‐stages, for example, reproduction and overwintering.

**Figure 1:**
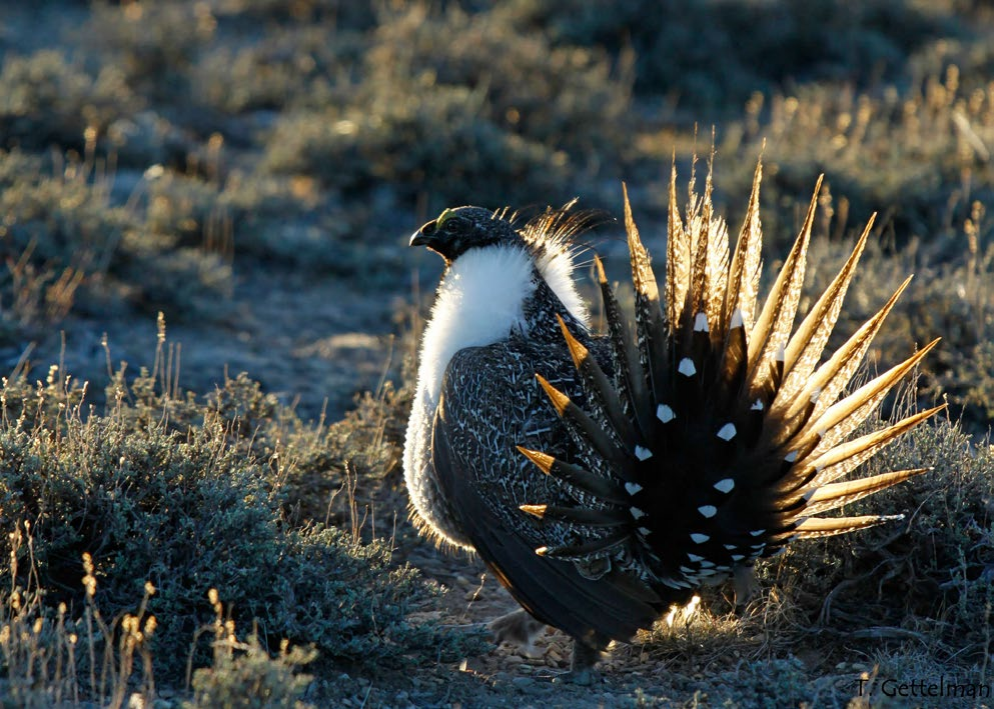
Male greater sage‐grouse (*Centrocercus urophasianus*) displaying on a communal breeding ground (i.e., lek) in the Great Basin of the United States. Photograph credit: Tatiana Gettelman

Greater sage‐grouse *Centrocercus urophasianus* (hereafter, sage‐grouse) are a sagebrush *Artemisia* spp. obligate species in western North America (Figure 1). Sage‐grouse populations have declined with the loss, degradation, and fragmentation of sagebrush ecosystems (Knick & Connelly, 2011), and they currently occupy slightly more than half of their former range (Miller, Chambers, Pyke, Pierson, & Williams, 2013; Schroeder et al., 2004). Habitat alterations include wildfire and subsequent conversion of sagebrush to cheatgrass *Bromus tectorum* (Chambers et al., 2014; Coates et al., 2016b; Coates, Ricca, et al., 2016), altered fire regimes (Baker, 2011), energy development (Doherty, Naugle, Walker, & Graham, 2008; Walker, Naugle, & Doherty, 2007), cropland conversion (Smith et al., 2016), and expansion of conifer trees into sagebrush‐dominated ecosystems (Baruch‐Mordo et al., 2013). Sage‐grouse are considered an indicator species within sagebrush ecosystems (Hanser & Knick, 2011) because they use a range of habitats at large spatial scales during the annual cycle, all of which are important for population persistence. Thus, management that conserves sage‐grouse is thought to support numerous sagebrush obligate and semiobligate species that function at smaller spatial scales (Rowland, Wisdom, Suring, & Meinke, 2006). Sage‐grouse habitat loss and population declines have led to multiple petitions for federal protection (U.S. Fish & Wildlife Service, 2015), and actions aimed at conserving sage‐grouse and improving habitat is an important component of state and federal land use policy and planning. Spatially explicit management tools are needed for quantitatively supporting policy decisions. Specifically, generalizable models from multiple sites that span a climate gradient can describe large geographical extents and can be extrapolated to areas where few field data are available. Such models are particularly important for sage‐grouse in Nevada and northeastern California that comprise a large part of the Great Basin where diverse climatic regimes likely lead to variation in primary productivity and pursuant habitat conditions.

Managers need tools that integrate variation across both space and time to improve understanding of habitat quality at scales necessary for conservation. Habitat selection assessments can be important to manage species both at broad extents and at local sites. Coates et al. (2016a) previously described methodology for integrating annual habitat selection indices with abundance and space use indices as management proxies (Stephens, Pettorelli, Barlow, Whittingham, & Cadotte, 2015) that delineate areas important for sage‐grouse conservation across a broader extent, but they did not consider seasonal variation in habitat needs known to be important for sage‐grouse (Atamian, Sedinger, Heaton, & Blomberg, 2010; Doherty et al., 2008; Hagen, Willis, Glenn, & Anthony, 2011; Knick & Connelly, 2011). For example, sage‐grouse rely almost exclusively on sagebrush for food during the winter (Wallestad, Peterson, & Eng, 1975) within snowless, windswept areas (Hagen et al., 2011), whereas they seek out forbs and sagebrush cover during spring nesting (Severson et al., 2017a) and mesic areas during summer brood rearing (Atamian et al., 2010). Here, we expand upon the previous framework to account for differences among functional responses across seasons as well as differences across sagebrush ecosystems at similarly broad spatial scales. Specifically, our objectives were to: (a) calculate seasonal resource selection functions (RSFs) for sage‐grouse across multiple study areas in Nevada and California for each of three seasons: spring, summer/fall and winter; (b) examine the strength and consistency of habitat selection effects across sites and seasons; (c) develop seasonal habitat maps that account for broad climatic variation that then inform an annual habitat selection map extrapolated across the region; and (d) calculate a continuous habitat management index (HMI), as well as a categorical habitat management map, that reflects both annual habitat selection and sage‐grouse abundance and space use.

## 2 MATERIALS AND METHODS

### 2.1 Study area

**Figure 2:**
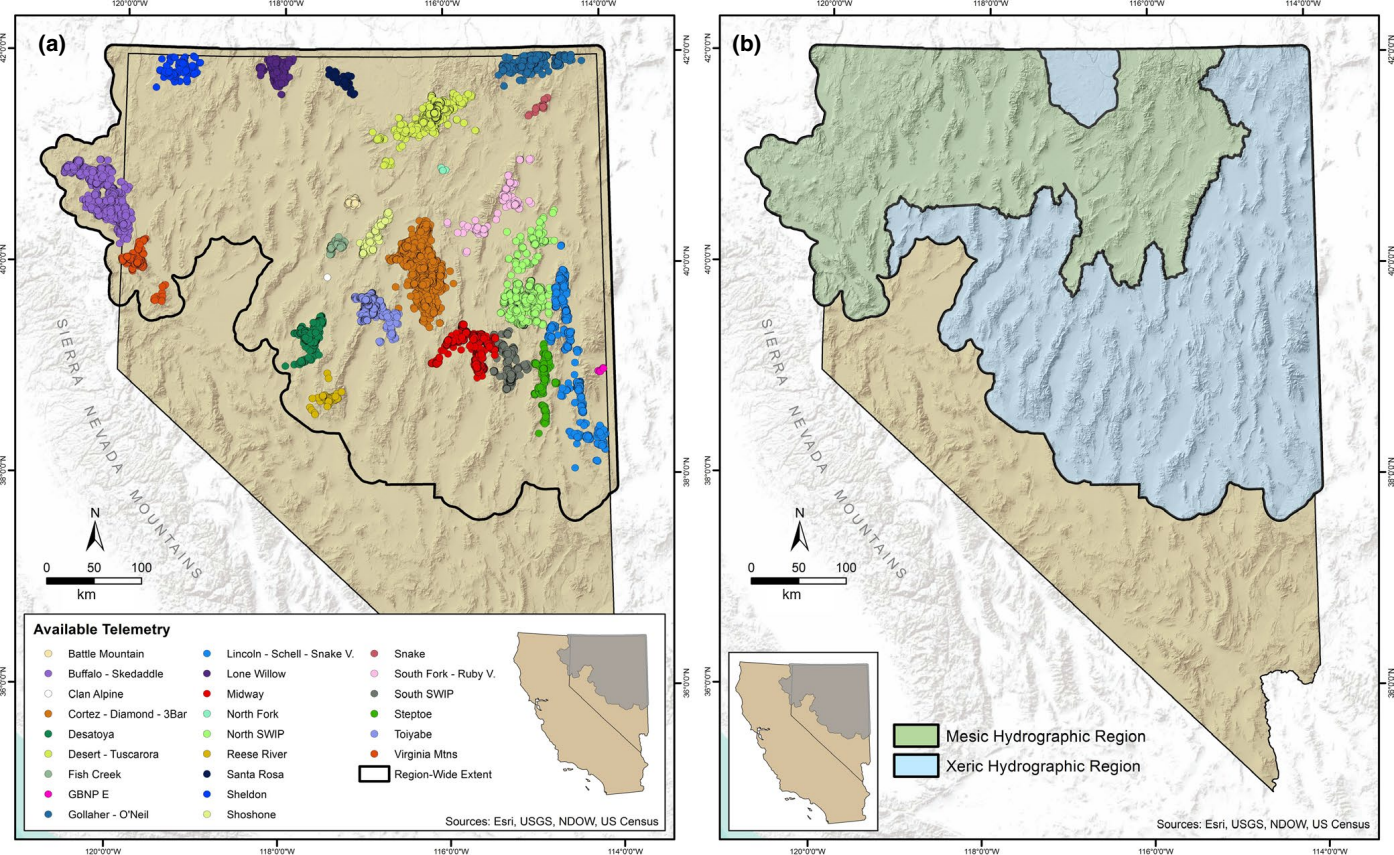
Region‐wide extent and telemetry points (colored dots) comprising greater sage‐grouse (*Centrocercus urophasianus*) locations available for use in resource selection function modeling: (a) Nevada and northeastern California; (b) northern (Mesic) and southern (Xeric) hydrographic subregions. Names refer to locations associated with Nevada Department of Wildlife Population Management Units

Our study area was determined as the outer perimeter of all combined sage‐grouse population management units (PMU; Sagebrush Ecosystem Technical Team, 2014) in Nevada and northeastern California, which were delineated by the state wildlife agencies. We include an 8.5 km buffer (Figure 2a) around PMU boundaries to accommodate the largest spatial scale of our habitat quality computations. Floristically, the region is dominated by Wyoming big sagebrush *A. tridentata wyomingensis* and black *A. nova* and low *A. arbuscula* sagebrush occurring at elevations below 2,100 m. At higher elevations, mountain big sagebrush *A. t. vaseyana* is more abundant. Single‐leaf pinyon pine *Pinus monophylla* and Utah juniper *Juniperus osteosperma* (hereafter, pinyon‐juniper) are native conifers but tend to colonize mid to high elevation and mesic sagebrush habitat. Cheatgrass is a non‐native, invasive annual grass that benefits from wildfire and often dominates and replaces sagebrush at low elevation following burns, especially in xeric environments (Chambers et al., 2014).

### 2.2 Sage‐grouse location data

We collected sage‐grouse location data over 16 years (1998–2014) at multiple study sites across Nevada and northeastern California (Figure 2a). Sage‐grouse were captured using standard spotlighting techniques (Wakkinen, Reese, Connelly, & Fischer, 1992) during spring and late summer/fall and fit with radio or global positioning system transmitters (GPS; GeoTrak, LLC). We sought to locate radio‐marked sage‐grouse weekly during spring and summer, and monthly during fall and winter. GPS‐marked birds were located remotely 10 times per day, on average, but only one randomly selected location per bird per day was used to reduce temporal and spatial autocorrelation. We further accounted for autocorrelation between points of the same bird, as well as variation in number of locations per bird, by fitting random effects structures (see Section 2.5).

### 2.3 Study sites and seasons

Telemetry locations of sage‐grouse were partitioned into 24 distinct sites based on spatial dispersion of marked sage‐grouse (Table 1 and Figure 2a). We did not develop seasonal habitat models for sites represented by <20 marked sage‐grouse or <100 telemetry locations within a single season. After data‐screening, we included telemetry data from 10 sites in the RSFs (Table 1). At each site, the spatial extent of habitat availability was defined by first calculating the minimum convex polygon (MCP) around all locations and then buffering each MCP by the average maximum daily sage‐grouse movement (1,451 m). Data from each site were divided into three seasons: spring from 16 March to 30 June; summer/fall from 01 July to 15 October; winter from 16 October to 15 March. We recognize methodology of using phenology to delineate seasons on an individual bird basis (Birkett, Vanak, Muggeo, Ferreira, & Slotow, 2012); however, we delineated seasons that were generalizable at the population level because the purpose of this study was to develop broad‐scale seasonal habitat maps for use by land and wildlife managers. Our seasons were slightly modified from Coates et al. (2013) and represented life history stages that corresponded to environmental conditions during breeding and nesting (spring), brood rearing (summer/fall) and nonbreeding (winter). While we acknowledge that annual variation in phenology could bias our estimates in some years, we believe our generalized estimates will provide the most useful information for managers in the widest variety of situations.

**Table 1:** Number of greater sage‐grouse (number of telemetry locations in parentheses) used to model seasonal habitat selection within Population Management Units (PMU) across Nevada and northeastern California

Sage‐Grouse PMU	Season		
	Spring	Summer/fall	Winter
Battle Mountain	6 (30)	11 (42)	10 (35)
Buffalo–Skedaddle	207 (1,748)*	174 (1,886)*	149 (565)*
Cortez–Diamond–3Bar	309 (4,142)*	216 (956)*	196 (541)*
Desatoya	26 (772)	28 (482)	15 (520)
Desert–Tuscarora	135 (2,556)*	115 (2,878)*	55 (1,519)*
Fish Creek	10 (70)	19 (126)	16 (64)
GBNP E	0 (0)	4 (8)	4 (26)
Gollaher–O'Neil	114 (1,523)*	120 (902)*	84 (307)*
Lincoln–Schell–Snake	57 (373)*	81 (509)*	67 (378)*
Lone Willow	76 (103)	73 (94)	73 (130)
Midway	60 (1,616)*	46 (1,646)*	22 (1,770)
North SWIP	121 (1,348)*	141 (1,644)*	74 (1,148)*
Reese River	10 (19)	10 (32)	9 (18)
Santa Rosa	10 (18)	9 (23)	9 (17)
Sheldon	18 (18)	19 (54)	21 (39)
Shoshone	11 (65)	12 (66)	10 (76)
Snake	5 (38)	4 (7)	0 (0)
South Fork–Ruby V.	25 (76)	20 (47)	32 (66)
South SWIP	60 (1,399)*	42 (1,246)*	42 (729)*
Steptoe	18 (134)	15 (88)	14 (80)
Toiyabe	95 (2,276)*	71 (2,676)*	52 (1,836)*
Virginia Mtns	96 (850)*	87 (341)*	53 (62)

* Season/site combinations used to train resource selection models. Within these sites, 20% of data were held aside for classification of habitat quality and validation. All other sites (no asterisk) were used for validation of spatial predictions.

### 2.4 Covariate description and spatial scales

Spatially explicit environmental covariates that were considered for RSF analysis are found in Table 2 and detailed in Table S1. We used 900‐m^2^ land cover layers derived from the National Land Cover Database (Xian, Homer, Rigge, Shi, & Meyer, 2015), which was represented as a continuous percent cover of bare ground, herbaceous perennial vegetation, big sagebrush (e.g., mountain big sagebrush, Wyoming big sagebrush), other sagebrush (e.g., low sagebrush), and nonsagebrush shrub (e.g., rabbitbrush [*Chrysothamnus* spp.], bitterbrush [*Purshia tridentata*]) as well as sagebrush height. We also used a 900‐m^2^ resolution layer depicting percent cover of conifers, mostly consisting of pinyon *Pinus* and juniper *Juniperus*, from recent high‐resolution mapping analyses (Gustafson et al., 2018). Other land cover types representing the dominant vegetation within 900‐m^2^ pixels were classified into binary raster layers using existing Landsat‐based mapping products and comprised agricultural cropland, annual grass (e.g., cheatgrass), forest other than pinyon‐juniper, riparian areas, and wet meadow as described in Coates et al. (2016a). To assess heterogeneity, we also calculated the number of land cover types and the number of edges between pixels of unique cover types within each assessed radius (i.e., scale). For all used and random (available) locations, we calculated the percent cover of land cover variables within radii of 167.9 m (8.7 ha), 439.5 m (61.5 ha), or 1,451.7 m (661.4 ha; neighborhood analysis tool in ArcGIS™ Spatial Analyst) representing averages of the minimum, mean, and maximum daily distance traveled by individual sage‐grouse (see Coates et al., 2016a). The purpose of evaluating different spatial scales for each variable stems largely from documented sage‐grouse selection of specific habitat characteristics at specific scales (Aldridge & Boyce, 2007; Aldridge, Saher, Childers, Stahlnecker, & Bowen, 2012; Casazza, Coates, & Overton, 2011; Doherty, Naugle, & Walker, 2010). To avoid issues with small sample sizes of locations in rare habitat types, land cover classes needed to account for >0.1% of the MCP at a site to be included in the analysis.

**Table 2:** Proposed variables assessed in resource selection function model development for each subregion, Nevada and northeastern California

Variable type	Scales
Annual grass	8.7, 61.5, 661.4 ha
Agriculture	8.7, 61.5, 661.4 ha
Bare ground	8.7, 61.5, 661.4 ha
Big sagebrush	8.7, 61.5, 661.4 ha
Forest	8.7, 61.5, 661.4 ha
Herbaceous	8.7, 61.5, 661.4 ha
Nonsagebrush shrubs	8.7, 61.5, 661.4 ha
Other sagebrush	8.7, 61.5, 661.4 ha
Pinyon‐juniper	8.7, 61.5, 661.4 ha
Riparian	8.7, 61.5, 661.4 ha
Wet meadow	8.7, 61.5, 661.4 ha
Sagebrush height	8.7, 61.5, 661.4 ha
Distance to cropland	Linear, exponential decay
Variety of edge types	8.7, 61.5, 661.4 ha
Variety of land cover types	8.7, 61.5, 661.4 ha
Any stream	Linear, exponential decay
Perennial stream	Linear, exponential decay
Intermittent stream	Linear, exponential decay
Spring	Linear, exponential decay
Water body	Linear, exponential decay
Wet meadow	Linear, exponential decay
Elevation	Linear (km)
Roughness index	1 ha
Topographic position index	510, 2,010 m

We measured Euclidean and exponential decay distances to landscape features that might affect probability of sage‐grouse use including water features, wet meadows, and cropland following similar procedures to Coates, Casazza, Ricca, Brussee, et al. (2016). Water features were obtained from the National Hydrography Dataset (U.S. Geological Survey, 2014) and included all streams, perennial streams, intermittent streams, springs, and open water bodies. Distance to wet meadows and cropland were identified on land cover maps and calculated to the nearest perimeter.

Topographic characteristics are often important to sage‐grouse habitat use (Baruch‐Mordo et al., 2013; Doherty et al., 2008; Severson et al., 2017a). Elevation of each location was determined from 30‐m digital elevation models (U.S. Geological Survey, 2009). Topographic roughness (Riley, DeGloria, & Elliot, 1999) was calculated within 1 ha of the locations and indicated the measured variability of the terrain. Topographic position index (TPI; De Reu et al., 2013), representing the concavity or convexity of the terrain, was calculated as the difference between elevation at the central point and the surrounding average elevation within radii of 510 and 2,010 m.

### 2.5 Modeling resource selection functions

#### 2.5.1 Step 1. Subregional RSF modelling by season

We followed similar procedures described in Coates et al. (2016a) to develop population‐level seasonal RSFs using generalized linear mixed‐effects models (GLMM). We quantified spatially explicit environmental variables that were associated potentially with seasonal habitat of sage‐grouse. A full list of variables and descriptions are found in Table S1. We categorized telemetry data into three subsets: (a) RSF model training subset (80%); (b) habitat category classification subset (10%); and (c) model prediction validation subset (10%). These subsets were comprised of randomly selected independent sage‐grouse; all locations from an individual sage‐grouse occurred in only one subset. Using the model training subset, we generated five random map locations for each used location throughout each buffered MCP to define habitat availability and account for environmental heterogeneity (Aldridge et al., 2012). Within our GLMMs, we included habitat covariates as fixed effects while year and individual sage‐grouse were included as random effects to account for nonindependent observations across space and within individuals (Gillies et al., 2006). We fit all models using the lme4 package (Bates, Mächler, Bolker, & Walker, 2015) in the R computing environment (R Core Team, 2014). Because models consisted of a larger sample of random locations than used locations, random locations were down‐weighted proportional to the ratio of used points to random points (i.e., weights, used = 1.0 and random = 0.2), so the two response classes received equal weight in the parameter estimation (Aldridge et al., 2012; Coates, Casazza, Ricca, Brussee, et al., 2016).

We used bias‐corrected Akaike's information criteria (AIC_c_; Burnham & Anderson, 2002) to identify the most parsimonious model for each site in a two‐step process. First, in analyses employing single covariates, we evaluated evidence among scale and distance functions. For each covariate under consideration (Table S1), we carried forward the spatial scale or distance function that fit two criteria: (1) lowest AIC_c_ of those considered and (2) AIC_c_ of 2.0 or more units lower than the null model (random effects only). Then, we produced a series of additive models containing all possible two‐covariate combinations from those covariates carried forward from step 1. Estimates of the partial coefficients in regression models become increasingly conditional on the other covariates in the model as collinearity among covariates increases thereby causing model‐averaged parameter estimates to be nonsensical under high multicollinearity (Cade, 2015). We therefore set a cutoff of |*r*| ≥ .65 and, for correlated variables, removed the variable that yielded a higher AIC_c_ value. We then calculated model‐averaged parameter estimates using the AICcmodavg package (Mazerolle, 2017) from the two‐covariate combination models. Covariates were excluded from the final RSF if model‐averaged 95% unconditional confidence intervals overlapped zero. This two‐step process allowed us to choose the most parsimonious scale for each variable or exclude variables that lacked evidence from the data altogether (Step 1) and estimate coefficients while accounting for variation from all other variables.

#### 2.5.2 Step 2. Evaluation of spatiotemporal variation in habitat selection

A strong, approximately north–south precipitation gradient exists within our overall study region (Miller et al., 2013), which was thought to influence resource availability across latitude. Thus, spatial variation in habitat attributes was analyzed by grouping sites into two subregions based on latitudinal precipitation differences within the northern Great Basin (Miller et al., 2013). We defined subregion boundaries using a modified hydrographic data layer (Mason, Ries, King, Thomas, & Baker, 1999), which divided our overall study area into mesic (wetter) and xeric (drier) areas (Figure 2b). Sites that overlapped the subregion contact boundary were classified to the subregion that included most site‐level telemetry locations. To evaluate spatiotemporal variation among functional responses for habitat characteristics, we employed meta‐analysis techniques using the site‐level seasonal parameter estimates and errors derived from RSFs for each covariate (R computing environment, package meta; Schwarzer, 2007). We calculated inverse variance (*I* ^2^), which is the effect of heterogeneity among estimated coefficients for each variable across different study sites, also known as the inconsistency other than expected by chance alone (Higgins, Thompson, Deeks, & Altman, 2003). This value was reported as a percentage of total variation across sites due to heterogeneity, where <25% = low inconsistency, >25% and <75% = moderate, and >75% = high (Higgins et al., 2003). The meta‐analyses were conducted for each subregion, as well as the entire study area collectively. This allowed us to derive coefficient estimates and 95% confidence intervals for each habitat variable across the different groups. We considered selection or avoidance to be consistent within each grouping if confidence intervals did not overlap zero.

### 2.6 Mapping sage‐grouse habitat

#### 2.6.1 Step 1. Accounting for subregional variation in habitat selection indices

Because resource selection functions for each combination of site and season consisted of values spanning orders of magnitude, we transformed each seasonal RSF to a habitat selection index (HSI) using procedures reported in Coates et al. (2016a). The HSI was not an absolute probability but was equivalent to a logistic transformation to express differences in habitat quality between zero and one for each grid cell. For each season, we averaged corresponding HSI pixel values across sites to derive a single continuous region‐wide seasonal surface (Coates et al., 2016a; Coates, Casazza, Ricca, Brussee, et al., 2016). To account for subregional variation in functional responses among variables, the averaged HSI values within each hydrographic subregions were divided by their respective maximum averaged value. This produced a relativized HSI for each subregion scaled between 0 and 1. For management purposes, each seasonal map was similarly relativized to allow equal weight among seasons and then multiplied together to produce a single composite HSI map of year‐round suitability. The purpose of this step was to produce a single composite map that accounted for habitat selection across all seasons. We masked 50‐m buffers around major roads, lakes, and urban areas to ensure the elimination of nonhabitat from the maps.

#### 2.6.2 Step 2. Seasonal and annual habitat categories

To aid in management planning, we binned seasonal and annual HSI maps into four discrete categories. We assigned HSI values to each location in the habitat category classification dataset (10% of the original telemetry data) and separated locations into mesic and xeric subregions. For each subregion, we calculated the HSI mean and *SD* and delineated HSI values: high: $\bar{x}-0.5$ *SD* to 1; moderate: $\bar{x}-1$ *SD* to $\bar{x}-0.5$ *SD*; low: $\bar{x}-1.5$ *SD* to $\bar{x}-1$ *SD*; nonhabitat: $<\bar{x}-1.5$ *SD* (Coates et al., 2016a).

We used three datasets to assess the accuracy of the habitat selection categories of all seasonal and annual maps (Coates et al., 2016a). The first set was comprised of locations from the 10% validation set within RSF sites. The second set was comprised of all telemetry locations from non‐RSF sites that did not meet the sampling criteria for RSF modeling described. This set was used to validate model predicted extrapolation because locations were not within sites used to develop seasonal RSFs. Within these two validation datasets, the number of locations for individual sage‐grouse was not equal, so we calculated the proportion of locations for each bird in each habitat category and used the average proportion for all birds as the validation statistic. The third validation dataset was active lek locations and was only used to validate the spring and the annual habitat maps. For all validation data, proportion of locations in habitat category was compared to expected proportion based on the habitat class area. To account for agreement by random chance, we calculated Cohen's kappa coefficients with values >0.75 indicating good agreement, 0.40–0.75 was acceptable, <0.40 was poor as suggested by Fleiss (1981). The purpose of the Cohen's kappa was to validate the habitat category map based on model predictions. Good or acceptable agreement indicated that independent data fell within the habitat categories at the expected proportions, meaning habitat was not over or under‐estimated.

#### 2.6.3 Step 3. Integrating indices of seasonal habitat and abundance and space use

For the purposes of updating useable spatially explicit tools for managers, we followed methodology detailed in Coates, Casazza, Ricca, Brussee, et al. (2016) to reconstruct and map example habitat categories for management application (habitat management categories; i.e., core, priority, general, nonhabitat). Specifically, we integrated seasonal HSIs with a composite probabilistic index of abundance and space use (AUI) derived from lek count and distributional data. We also created a habitat management index (HMI) as a continuous surface by multiplying the composite HSI with AUI for additional management and policy decision support for use in quantitative conservation planning tools (e.g., Ricca et al., 2018).

## 3 RESULTS

We compiled 44,853 telemetry locations (*n* = 1,799 individual sage‐grouse) including 19,174, 15,753, and 9,926 locations in spring, summer/fall, and winter, respectively (Table 1). We had sufficient data to produce habitat models at 10 sites in spring, 10 sites in summer/fall, and 7 sites in winter, resulting in 27 sites by season RSF models (Table 1). Details regarding variables constituting evidence for seasonal RSFs (Table S2), model‐averaged parameters (Table S3), and means with standard errors of used and available locations (Table S4) for each study site are provided in Supplementary Materials.

### 3.1 Habitat selection meta‐analysis

**Figure 3:**
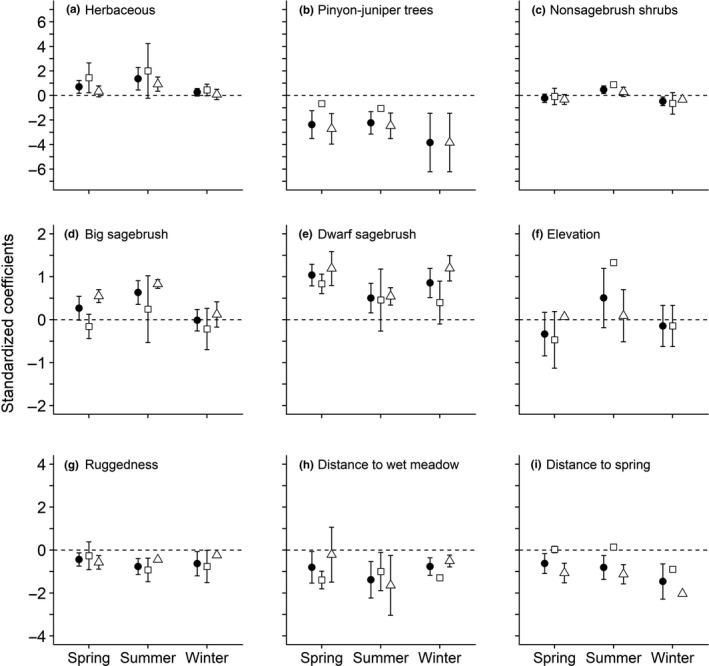
Seasonal meta‐analysis of parameter estimates for important habitat components affecting habitat selection of greater sage‐grouse at multiple sites in Nevada and California. (a) Herbaceous (grasses and forbs); (b) pinyon‐juniper trees; (c) nonsagebrush shrubs; (d) big sagebrush; (e) dwarf sagebrush; (f) elevation; (g) ruggedness; (h) distance to wet meadow; and (i) distance to spring. Estimates were combined across subregions (closed circle), within the mesic subregion (open square), and within the xeric subregion (open triangle). Negative coefficients on distance‐based covariates are evidence for selection. Error bars represent 85% confidence limits

Functional responses varied spatially and temporally for most covariates, but some consistent patterns were evident among nine primary covariates (Figure 3a–i). We found selection for greater perennial herbaceous cover throughout all seasons and sites, with the strongest effect in the mesic subregion and during summer/fall (Figure 3a). However, selection for greater herbaceous cover varied across sites based on meta‐analysis results in which the *I* ^2^ of herbaceous vegetation selection for combined sites and the mesic subregion during summer/fall were both 100%. During spring, areas with greater herbaceous cover were consistently selected at mesic sites, whereas weaker selection occurred in xeric sites (Figure 3a). During winter, evidence of selection for herbaceous cover was substantially less, especially at the xeric sites (Figure 3a).

Sage‐grouse exhibited variation in selection and avoidance based on shrub and tree composition. Sage‐grouse showed consistent evidence of avoidance of pinyon‐juniper (Figure 3b). However, the strength of avoidance varied among hydrographic subregion, leading to heterogeneity in estimates across sites (*I* ^2^ > 99% for all seasons and subregions). Avoidance of conifers was much greater within the xeric compared to the mesic subregion (Figure 3b). Sage‐grouse selected nonsagebrush shrub cover types at all sites within the mesic subregion during the summer/fall but tended to avoid this type of cover during spring and winter (Figure 3c). Sagebrush shrub cover types typically were selected across both hydrographic subregions and all seasons (Figure 3d,e) with evidence of spatiotemporal variation among different sagebrush communities. For example, although sage‐grouse selected big sagebrush cover types during spring and summer/fall months, evidence of selection was much greater at the xeric compared to the mesic subregion. Sage‐grouse also consistently selected species of dwarf sagebrush (Figure 3e), but variation in this effect was strong within mesic sites during summer/fall and winter.

**Figure 4:**
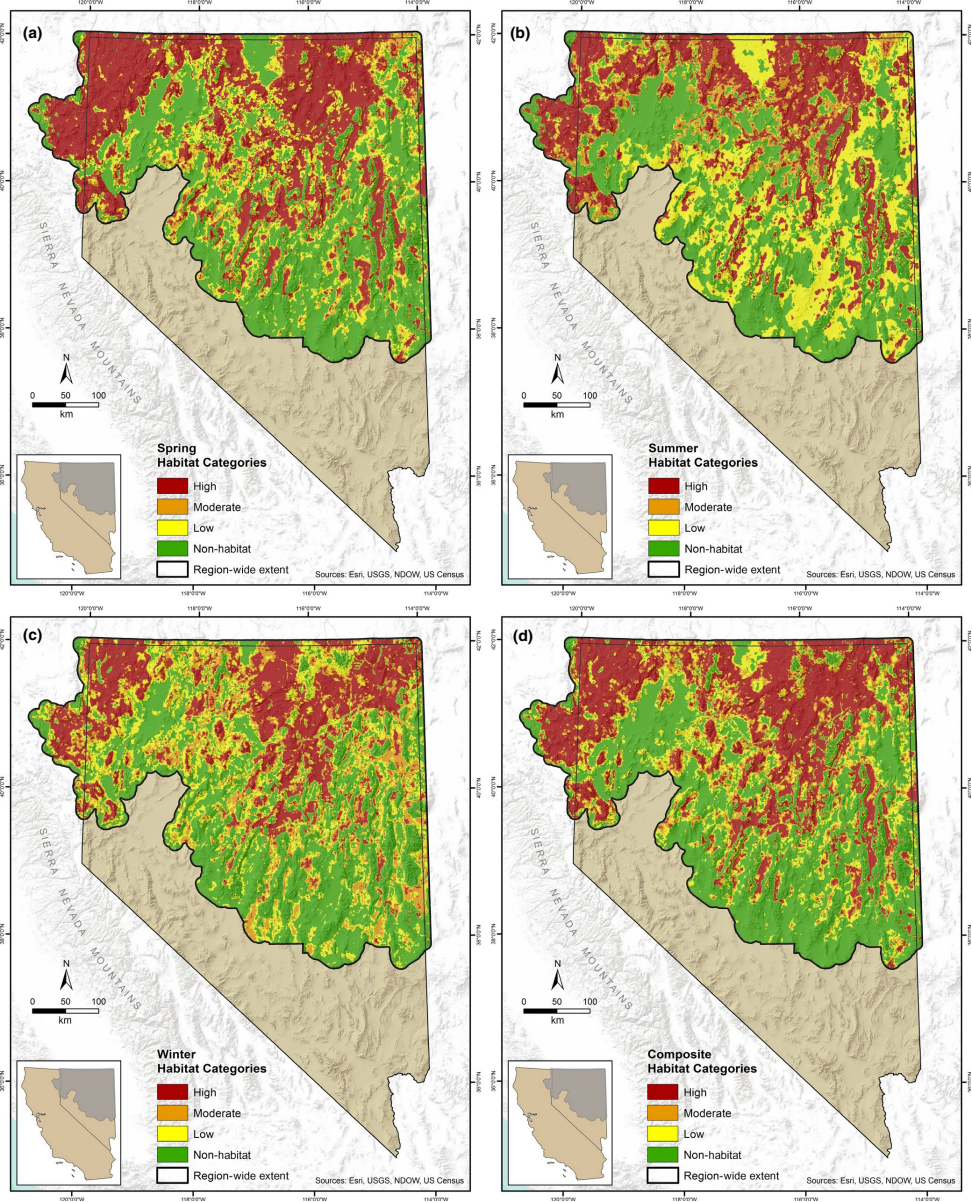
Region‐wide distribution of categorized habitat selection for greater sage‐grouse (*Centrocercus urophasianus*) within Nevada and northeastern California during: (a) spring; (b) summer/fall; (c) winter; and (d) all‐seasons composite

Water sources were generally important to sage‐grouse in mesic and xeric subregions. Specifically, we found stronger selection for wet meadows and upland springs (Figure 4h,i), where confidence intervals did not overlap zero, compared to other water sources such as agriculture and wooded riparian areas (Table S5). During summer/fall, and in the xeric subregion, sage‐grouse were most likely to select upland springs over wet meadows. We observed evidence of selection for higher elevations during summer/fall in both hydrographic subregions (Figure 4f), and consistent avoidance of topographically rugged areas (Figure 4g). For more detailed results of meta‐analysis see Table S5.

### 3.2 Mapping and validation

**Figure 5:**
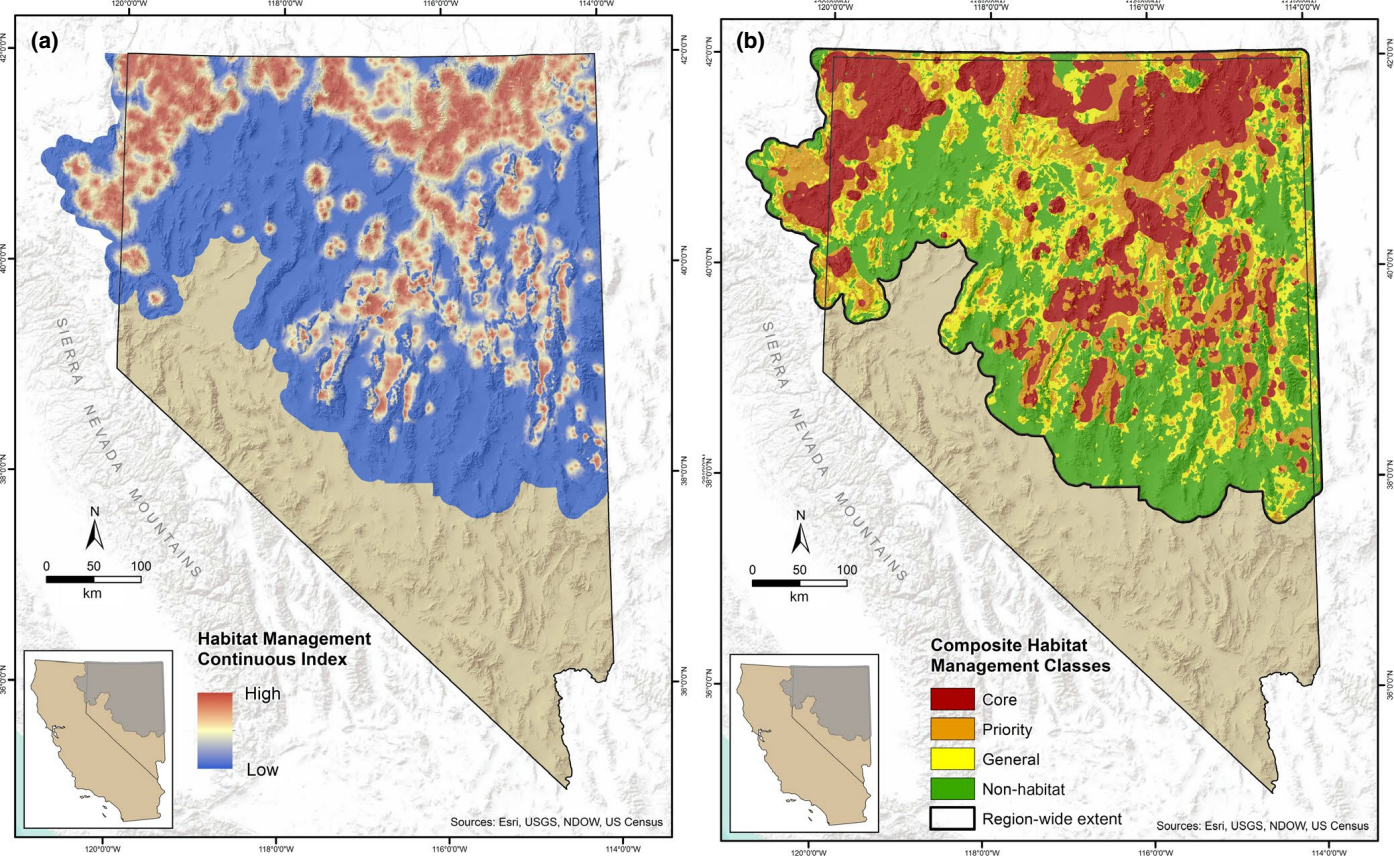
Integration of indices of seasonal habitat selection from fine‐resolution telemetry data and abundance and space use from course resolution lek survey data for greater sage‐grouse populations within Nevada and northeastern California presented as: (a) a habitat management continuous index; and (b) a habitat management categorization

We produced habitat category maps for each of the three seasons (Figure 5a–c), as well as a composite annual HSI (Figure 5d). We used 3,766 locations (460 independent sage‐grouse) for classification and 10,402 locations (1,087 sage‐grouse) for validation (4,354 within RSF training areas and 6,048 outside RSFs). For telemetry (RSF and non‐RSF) and lek validation data, percentages of locations falling within cumulative habitat were mostly considered acceptable or good (Table 3).

**Table 3:** Summary of habitat selection model validation tests and Cohen's Kappa coefficient (*κ*) used to evaluate habitat selection classes based on *SD* percentiles for seasonal maps for greater sage‐grouse (*Centrocercus urophasianus*), Nevada and northeastern California

Season	Habitat classification	Expected %	Validation sets		
			RSF subregions % (*κ*)	Non‐RSF subregions % (*κ*)	Active leks % (*κ*)
Spring	High	69	73 (0.92)	81 (0.97)	70 (0.90)
	Moderate	15	14 (0.99)	8 (0.99)	11 (0.69)
	Low	9	8 (0.91)	6 (0.96)	8 (0.90)
	Nonhabitat	7	5 (0.94)	4 (0.99)	7 (0.96)
Summer	High	69	74 (0.91)	62 (0.74)	N/A
	Moderate	15	10 (0.97)	22 (0.87)	N/A
	Low	9	7 (0.74)	14 (0.78)	N/A
	Nonhabitat	7	8 (0.76)	3 (0.83)	N/A
Winter	High	69	57 (0.28)	26 (0.70)	N/A
	Moderate	15	16 (0.81)	19 (0.98)	N/A
	Low	9	13 (0.31)	42 (0.82)	N/A
	Nonhabitat	7	13 (0.51)	13 (0.87)	N/A
Composite	High	69	68 (0.75)	72 (0.89)	79 (0.74)
	Moderate	15	15 (0.92)	17 (0.95)	9 (0.72)
	Low	9	9 (0.78)	6 (0.99)	8 (0.94)
	Nonhabitat	7	7 (0.41)	5 (0.93)	3 (0.67)

For conservation planning and analyses, we created a continuous HMI (Figure 4a) and map of composite habitat management categories (Figure 4b) based on seasonal HSI integrated with AUI. Management classifications of core, priority, general, and nonhabitat represented 25.2% (5,323,129 ha), 20.1% (4,245,741 ha), 18.5% (3,903,734 ha), and 36.3% (7,672,414 ha), respectively, of study extent within Nevada and northeastern California.

## 4 DISCUSSION

We present a spatially explicit model that integrates regional hydrographic and intra‐annual variation in habitat selection into a single, broad‐scale predictive habitat map for sage‐grouse occupying Nevada and northeastern California, which is the majority of the Great Basin. Accounting for variation among functional habitat responses increases accuracy and can aid conservation planning for sage‐grouse at local and region‐wide scales. We generated seasonal maps that delineate important regional variation in habitat use by sage‐grouse and likely reflect requirements specific to individual life history stages of sage‐grouse occupying environments that vary in precipitation and other habitat attributes. Our models corroborate general findings reported previously for sage‐grouse but also expand on sage‐grouse ecology by disentangling spatial and temporal variation in habitat selection patterns. This analysis also expands on previous studies that combine habitat quality indices (i.e., HSI) based on fine‐scale telemetry data with abundance and space use indices that rely on course‐scale lek survey data (Coates, Casazza, Ricca, Brussee, et al., 2016; Ricca et al., 2018) as management proxies (Stephens et al., 2015) to refine a unified distributional model and help guide management decisions.

Although our resource selection findings generally aligned with well‐known aspects of sage‐grouse ecology (Connelly, Knick, Schroeder, & Stiver, 2004), we provide evidence of clear differences in functional responses for specific habitat characteristics among seasons and hydrographic subregions. Many of these differences likely arise from changing life history needs of sage‐grouse and associated habitat attributes across the annual cycle, which need to be understood to facilitate effective conservation. For example, sage‐grouse were more likely to select areas with increased perennial herbaceous cover during months that correspond to their reproduction (spring and summer). In particular, the mesic subregion had greater availability (and stronger selection) of grasses and forbs during nesting and brood‐rearing phases of reproduction likely in response to greater annual precipitation relative to the xeric subregion. The concordant availability and selection of this seasonal habitat feature in mesic areas suggest that sage‐grouse are exploiting an advantage to occupying herbaceous cover at this life stage. Herbaceous cover primarily consisted of deep‐rooted perennial grasses and forbs which are known to provide critical concealment cover for nests and chicks (Barnett & Crawford, 1994; Gregg, Barnett, & Crawford, 2008; Gregg, Crawford, Drut, & DeLong, 1994). During the brood‐rearing stage, perennial forbs provide forage (Atamian et al., 2010; Casazza et al., 2011; Drut, Pyle, & Crawford, 1994; Hagen, Connelly, & Schroeder, 2007) and are associated with greater diversity of invertebrates that are an important dietary component for growing chicks (Gregg & Crawford, 2009; Klebenow & Gray, 1968; Peterson, 1970). However, the reduction in strength of selection for herbaceous cover from spring and summer to winter, especially at xeric sites, is associated with a shift in diet to primarily sagebrush plants and heavy use of shrubs as cover (Patterson, 1952; Wallestad et al., 1975). This switch likely occurs when herbaceous cover is desiccated while the presence and dietary needs for insects by juvenile grouse has diminished, and juvenile grouse are achieving adult‐like size and plumage.

Our seasonal modeling provides strong evidence of avoidance of pinyon‐juniper stands and forest cover types across all sites and seasons, although the degree of avoidance varied. Multiple studies have shown that pinyon‐juniper decreases habitat quality for sage‐grouse (Bates, Miller, & Svejcar, 2000; Miller, Svejcar, & Rose, 2000; Severson et al., 2017b). Additionally, areas dominated by sagebrush with dispersed conifers can increase avian predators (Howe, Coates, & Delehanty, 2014) and lead to decreased annual survival rates (Casazza et al., 2011; Coates, Prochazka, et al., 2017) and eventual extirpation of leks (Baruch‐Mordo et al., 2013). As conifer abundance increases, the area will be avoided, even if desirable sagebrush is still present (Coates, Prochazka, et al., 2017; Severson et al., 2017a). Strong evidence of conifer avoidance across every season and hydrographic area corroborates the findings of other studies (Doherty et al., 2008; Freese, 2009; Severson et al., 2017a) that were focused on fewer sites and specific seasons. Our findings give further weight to the deleterious effects of conifers on sage‐grouse and point to conifer management in areas of recent expansion into sagebrush to improve sage‐grouse habitat and increase sage‐grouse reproductive success (Severson et al., 2017b, 2017c; Severson, Hagen, Tack, et al., 2017).

Although sage‐grouse demonstrated preference for sagebrush across both subregions, we observed greater selection for dwarf sagebrush species (e.g., black and low sagebrush) than big sagebrush species (e.g., Wyoming and mountain sagebrush). Within the Great Basin, dwarf sagebrush species are increasingly recognized as important to sage‐grouse during winter (Hagen et al., 2011), spring (Musil, 2011), and summer (Freese, Petersen, Miller, Yost, & Robinson, 2016; Severson et al., 2017a). Low sagebrush is particularly important during winter months (Hagen et al., 2011) probably due to its palatability (Rosentreter, 2004). Also, while short in stature, low sagebrush often is found on relatively snow‐free, windswept ridges where it is available to sage‐grouse in winter. Selection for dwarf sagebrush and less herbaceous cover was greatest in winter and spring and may represent sage‐grouse moving to lower elevation to access sagebrush as forage during periods of adverse weather conditions.

Although our study demonstrated greater selection for dwarf sagebrush, we do not dismiss significant associations between sage‐grouse and big sagebrush species, as well as variation that exists in selection among hydrographic regions and seasons. Big sagebrush was most important within the xeric compared to mesic region, especially during reproduction. We emphasize the importance of big sagebrush for sage‐grouse nesting and brood rearing, especially in environments where grasses and forbs are naturally less abundant. Mountain big sagebrush is generally found in productive habitat at high elevation, whereas Wyoming big sagebrush is generally found in drier, less productive areas at lower elevation (Freese et al., 2016; Severson et al., 2017a). Sage‐grouse often prefer mountain big sagebrush over Wyoming big sagebrush as forage (Welch, Wagstaff, & Roberson, 1991). Differential selection among big sagebrush species is also supported in our study by evidence of sage‐grouse selecting areas with big sagebrush during the summer, coinciding with typical sage‐grouse movements to productive, mesic areas comprised of mountain big sagebrush during periods of low precipitation.

Our findings also corroborate studies that report selection of limited mesic resources within the semiarid sagebrush ecosystems (Casazza et al., 2011), particularly during summer/fall months when forbs and associated invertebrates are unavailable elsewhere. Specifically, in the xeric subregion, sage‐grouse often selected upland springs that are typically surrounded by big sagebrush, whereas in mesic subregions large wet meadows were selected more than springs. The commonality here is that sage‐grouse seek forbs and insects during reproduction, wet meadows if available, and springs if meadows are not available. Interestingly, sage‐grouse typically avoided woody riparian areas among sites (Table S3), which also provide surface water but are accompanied by woody vegetation (e.g., usually aspen trees [*Populus tremuloides*]). This is consistent with sage‐grouse avoiding trees and corresponding aerial predators despite the presence of surface water and forbs. Trees in riparian areas provide perching substrate for raptors in sagebrush ecosystems (Coates, Howe, Casazza, & Delehanty, 2014), and presumably sage‐grouse perceive this threat despite presence of water resources. In addition, the numerous mountain ranges and associated rain‐shadows of the northern Great Basin results in a mix of xeric and mesic habitats available to sage‐grouse (Miller & Eddleman, 2001), and some important mesic areas were associated with valley bottoms that contained nonsagebrush shrub (salt scrub flats). We surmise that sage‐grouse selected these areas specifically to benefit from associated water resources during late summer and fall, which helps explain the observed increase in the use of nonsagebrush shrub during this period for both hydrographic subregions. Many nonsagebrush shrub species are likely to provide concealment cover for sage‐grouse while seeking out important and limited water resources. We also observed variability in selection/avoidance patterns for agriculture among sites (Table S3), which provides water resources to sage‐grouse via surface irrigation. Selection for irrigated pastures (i.e., alfalfa) is likely a function of site‐level availability of natural water sources. Large‐scale studies using lek data have generally found negative effects of agriculture on sage‐grouse populations (Doherty, Evans, Coates, Juliusson, & Fedy, 2016; Wisdom, Meinke, Knick, & Schroeder, 2011). However, smaller‐scale studies based on telemetry data have yielded variable results (Aldridge & Boyce, 2007; Connelly, Browers, & Gates, 1988). Sage‐grouse often use alfalfa fields for summer/fall habitat, especially if upland wet meadow habitat is limited (Connelly, Rinkes, & Braun, 2011). Unfortunately, the effects on sage‐grouse demographic rates of selecting lower elevation agricultural fields as opposed to higher elevation wet meadow areas remain unclear.

Our study was not without limitations. Because our goal was to provide a relatively straightforward technique that resulted in broad‐scale habitat and management maps, we assigned specific dates as seasonal categories to telemetry locations across all years and individual sage‐grouse. The alternative would be to allow seasons to vary by year based on phenology and individual sage‐grouse behavior. This latter method would require an in‐depth prerequisite movement analysis for each sage‐grouse, which presents major challenges given the sparsity of data from VHF marked birds in our data set. Misclassification of locations into specific seasons should be minimal because few locations were associated with between‐season transitions. Additionally, seasonal inferences should not be biased because annual variation based on phenology is assumed to be a random process with normally distributed errors. We also recognize that the generalized seasons may not fully represent specific needs for reproductive life history phases for females (e.g., nesting and brood rearing) because males and nonreproducing females were pooled in our analysis. Research at similar spatial scales that extends our study by integrating indices of RSFs across reproductive life phases (e.g., nesting female only) with indices of demographic responses (e.g., nest survival) given underlying habitat covariates would further elucidate sage‐grouse habitat and refine large‐scale habitat management maps.

Another important limitation is our analysis could not characterize some potentially important microhabitat characteristics, which are typically measured using field techniques. Because recent studies have demonstrated the importance of microhabitat and similar variation in functional responses among life‐stages (Coates, Brussee, et al., 2017; Gibson, Blomberg, Atamian, & Sedinger, 2016), effective habitat management strategies might couple GIS‐derived habitat maps with finer resolution field measurements of microhabitat to fully assess sage‐grouse habitat at multiple spatial scales. Furthermore, GIS datasets used here were derived from imagery captured in summer/fall months and might not accurately depict grass and forb percentages during winter. However, these variables should correlate with residual overwinter vegetation that may be used by sage‐grouse as cover or occasionally as food, especially given our study areas within the Great Basin typically receive relatively little or no snow during winter.

Overall, the seasonal modeling framework presented here performed well, particularly for the critically important breeding season (Taylor, Walker, Naugle, & Mills, 2012). Our composite annual HSI should prove valuable for managers and conservationists as it reflects the year‐round relative importance of areas for sage‐grouse populations. However, coupling this composite HSI with single seasonal HSIs will be more useful for identifying specific seasonal effects. Using the seasonal HSIs, other calculations are possible that may be conducive to addressing specific management objectives. For example, indexing the maximum value across all seasons could represent a maximum selection potential for any given area. Considering that sage‐grouse move between seasonal areas, a spatial mosaic of high seasonal HSIs may also prove beneficial. Although limitations are inherent in any approach, our seasonal HSIs and habitat category maps (Supplementary Materials) allow users to target calculations that might be most informative for their specific management objective. Recognizing spatiotemporal variation in habitat indices is crucial to aligning sage‐grouse ecology with effective land management decisions. Predicting sage‐grouse responses to simulated management actions using spatially explicit support tools (Ricca et al., 2018) is an important next step for quantifying the effectiveness of various conservation action scenarios. The joint–index modeling framework and mapping products provided here support this effort for sage‐grouse and may be applied to other species that exhibit seasonal shifts in habitat requirements.

## CONFLICT OF INTEREST

None declared.

## AUTHORS' CONTRIBUTIONS

In alphabetical order: B.E.B., D.J.D., M.A.R., M.L.C., P.S.C. contributed to original idea, design, experiment; D.J.D., M.L.C., P.S.C., S.C.G. contributed to collecting data and conducting research; B.E.B., D.J.D., J.P.S., K.B.G., M.A.R., M.L.C., S.C.G., S.P.E., P.S.C. wrote or provided substantial edits; B.E.B., K.B.G., M.A.R., M.L.C., P.S.C. helped develop and design methodology; B.E.B., J.P.S., K.B.G., M.A.R., P.S.C. contributed to data analysis; D.J.D., M.L.C., S.C.G., S.P.E., P.S.C. contributed substantial materials, resources, or funding.

## Data Availability

Spatially explicit GIS files, including (a) study site locations, (b) abundance and space use index, (c) habitat selection index, (d) habitat categories, (e) habitat management continuous index, (f) management categories, and (g) model input layers, are available at ScienceBase repository at: http://dx.doi.org/10.5066/F7CC0XRV (Coates et al., 2016b). Lek and telemetry data quality assurance, storage and management are carried out by Nevada Department of Wildlife (S. P. Espinosa) and California Department of Fish and Wildlife (S. C. Gardner). Example script for all analyses is provided in Appendix S1.
