# Supplementary material for: Spatially explicit models of seasonal habitat for greater sage‐grouse at broad spatial scales: Informing areas for management in Nevada and northeastern California
Source: Ecol Evol. 2019 Nov 25;10(1):104–18. doi: 10.1002/ece3.5842 (PMC6972839; doi:10.1002/ece3.5842)
Supplement: Supplementary file 1 [file ECE3-10-104-s001.docx]

**SUPPLEMENTARY MATERIAL**

**Table S1.** All spatial variables, metrics, scales, and sources of data considered in the analyses of Greater Sage-Grouse resource selection function model development for each subregion, Nevada and northeastern California.

| Type | Metric | Scales (m) | Sources |
| --- | --- | --- | --- |
| Vegetation & Land Cover |  |  |  |
| Annual grass cover | 1 | 167, 439, 1451 | LANDFIRE 2010 |
| Agriculture cover | 1 | 167, 439, 1451 | LANDFIRE 2010 |
| Bare ground | 2 | 167, 439, 1451 | Xian et al. 2015 |
| Big sagebrush | 2 | 167, 439, 1451 | Xian et al. 2015 |
| Forests | 1 | 167, 439, 1451 | LANDFIRE 2010 |
| Herbaceous canopy cover | 2 | 167, 439, 1451 | Xian et al. 2015 |
| Non-sagebrush shrub canopy cover | 2 | 167, 439, 1451 | Xian et al. 2015 |
| Other sagebrush canopy cover | 2 | 167, 439, 1451 | Xian et al. 2015 |
| Pinyon-juniper canopy cover | 2 | 167, 439, 1451 | Gustafson et al. 2018 |
| Riparian areas | 1 | 167, 439, 1451 | LANDFIRE 2010 |
| Wet meadows | 1 | 167, 439, 1451 | LANDFIRE 2010 |
| Sagebrush shrub heights | 3 | 167, 439, 1451 | Xian et al. 2015 |
| Distance to cropland | 4, 5 | Linear, Exponential decay | LANDFIRE 2010 |
| Variety of edge types | 6 | 167, 439, 1451 | LANDFIRE 2010 |
| Variety of land cover types | 7 | 167, 439, 1451 | LANDFIRE 2010 |
| Water & Streams |  |  |  |
| Distance to Any Stream | 4, 5 | Linear, Exponential decay | U.S. Geological Survey 2017 |
| Distance to Perennial Stream | 4, 5 | Linear, Exponential decay | U.S. Geological Survey 2017 |
| Distance to Intermittent Stream | 4, 5 | Linear, Exponential decay | U.S. Geological Survey 2017 |
| Distance to Spring | 4, 5 | Linear, Exponential decay | U.S. Geological Survey 2017 |
| Distance to Waterbody | 4, 5 | Linear, Exponential decay | U.S. Geological Survey 2017 |
| Distance to Wet Meadow | 4, 5 | Linear, Exponential decay | LANDFIRE 2010 |
| Topography |  |  |  |
| Elevation | 3 | Linear (km) | U.S. Geological Survey 2009 |
| Roughness | 3 | 1 ha | U.S. Geological Survey 2009, Evans et al. 2014 |
| Topographic position index | 3 | 510 m, 2,010 m | U.S. Geological Survey 2009, Evans et al. 2014 |

Metric Legend:

1 = For each cover type, pixels were classified as either dominant (1) or not (0). Then for each scale, we calculated proportions of each cover type.

2 = For each cover type, calculated the mean value of continuous percent cover of that vegetation type at each spatial scale.

3 = Calculated the mean value of a continuous variable at each spatial scale

4 = Calculated Euclidean distance to linear or point features

5 = For each study region and season, transformed distances to linear or point features using exponential decay function using using $\exp(-d/\alpha)$ where α is the mean distance of used points to the point or linear feature.

6 = Defined edges as boundaries between dominant cover types. Count of number of different edge types within each spatial scale.

7 = Count of number of different dominant cover types within each spatial scale

References

Evans, J. S., J. Oakleaf, S. A. Cushman, and D. Theobald. 2014. An ArcGIS Toolbox for Surface Gradient and Geomorphometric Modeling, version 2.0-0.

Homer, C.G., J. Dewitz, L. Yang, S. Jin, P. Danielson, P., Xian, J. Coulston, N. Herold,

J. Wickham and K. Megown. 2015. Completion of the 2011 National Land Cover

Database for the conterminous United States – representing a decade of land cover

change information. Photogrammetric Engineering and Remote Sensing, Vol. 81, 345

353.

Gustafson, K. B., P. S. Coates, C. L. Roth, M. P. Chenaille, M. A. Ricca, E. Sanchez-Chopitea,

and M. L. Casazza. 2018. Using object-based image analysis to conduct high-resolution

conifer extraction at regional spatial scales. International Journal of Applied Earth

Observation and Geoinformation 73:148–155.

LANDFIRE. 2010. Existing Vegetation Type Layer, LANDFIRE 1.2.0, U.S. Department of the

Interior, Geological Survey. Available at <https://www.landfire.gov>.

U.S. Geological Survey. 2009. National Elevation Dataset.

U.S. Geological Survey. 2017b. National Hydrography Dataset. <https://nhd.usgs.gov>.

Xian, G., C. Homer, M. Rigge, H. Shi, and D. Meyer. 2015. Characterization of shrubland ecosystem components as continuous fields in the northwest United States. Remote Sensing of Environment 168:286–300.

**Table S2.** Variable selection results from the “proposal set” of variables sage-grouse seasonal resource selection function (RSF) models at 10 sites within in Nevada and northeastern California. The top-ranked variable in each set was retained in the suite of candidate variables for RSF modeling if it performed better than the null model and if confidence intervals around estimated mean effects did not overlap zero.

Site Season Group Covariate Scale K LL ΔAICc w

Buffalo-Skedaddle Spring Land cover Annual grass Null 3 -8934.7 0.0 0.41

8.7 ha 4 -8934.0 0.7 0.29

61.5 ha 4 -8934.6 1.9 0.16

661.4 ha 4 -8934.7 2.0 0.15

Bare ground 61.5 ha 4 -8909.4 0.0 0.99

8.7 ha 4 -8914.3 9.8 0.01

661.4 ha 4 -8922.5 26.1 0.00

Null 3 -8934.7 48.4 0.00

Big sagebrush 661.4 ha 4 -8829.8 0.0 1.00

61.5 ha 4 -8887.0 114.4 0.00

8.7 ha 4 -8911.0 162.3 0.00

Null 3 -8934.7 207.7 0.00

Cropland 661.4 ha 4 -8753.1 0.0 1.00

61.5 ha 4 -8828.3 150.4 0.00

8.7 ha 4 -8868.7 231.3 0.00

Null 3 -8934.7 361.2 0.00

Forest 661.4 ha 4 -8669.0 0.0 1.00

61.5 ha 4 -8711.3 84.7 0.00

8.7 ha 4 -8768.8 199.7 0.00

Null 3 -8934.7 529.4 0.00

Herbaceous 661.4 ha 4 -8749.9 0.0 1.00

61.5 ha 4 -8783.8 67.8 0.00

8.7 ha 4 -8798.8 97.9 0.00

Null 3 -8934.7 367.6 0.00

Non-sagebrush shrub 61.5 ha 4 -8803.4 0.0 1.00

661.4 ha 4 -8824.4 41.9 0.00

8.7 ha 4 -8837.1 67.3 0.00

Null 3 -8934.7 260.5 0.00

Other sagebrush 661.4 ha 4 -8911.8 0.0 1.00

61.5 ha 4 -8917.2 10.8 0.00

8.7 ha 4 -8917.7 11.9 0.00

Null 3 -8934.7 43.8 0.00

Riparian 661.4 ha 4 -8600.1 0.0 1.00

61.5 ha 4 -8733.0 265.8 0.00

8.7 ha 4 -8817.9 435.6 0.00

Null 3 -8934.7 667.1 0.00

Wet meadow 661.4 ha 4 -8841.5 0.0 1.00

61.5 ha 4 -8889.6 96.2 0.00

8.7 ha 4 -8914.5 146.0 0.00

Null 3 -8934.7 184.3 0.00

Sagebrush height Sagebrush height 661.4 ha 4 -8900.2 0.0 1.00

61.5 ha 4 -8924.1 47.9 0.00

8.7 ha 4 -8930.0 59.6 0.00

Null 3 -8934.7 67.0 0.00

Agriculture Distance to cropland Expon. decay 4 -8732.0 0.0 1.00

Linear 4 -8778.3 92.6 0.00

Null 3 -8934.7 403.4 0.00

Edge Variety of edge cover types 61.5 ha 4 -8184.6 0.0 1.00

661.4 ha 4 -8259.5 149.9 0.00

8.7 ha 4 -8363.6 358.2 0.00

Null 3 -8934.7 1498.2 0.00

Landscape variation Variety of land cover types 61.5 ha 4 -8135.4 0.0 1.00

8.7 ha 4 -8298.7 326.6 0.00

661.4 ha 4 -8605.6 940.4 0.00

Null 3 -8934.7 1596.6 0.00

Water sources Distance to perennial stream Expon. decay 4 -8717.2 0.0 1.00

Distance to spring Expon. decay 4 -8760.9 87.3 0.00

Distance to spring Linear 4 -8800.8 167.1 0.00

Distance to perennial stream Linear 4 -8828.7 223.0 0.00

Distance to water body Linear 4 -8879.3 324.2 0.00

Distance to water body Expon. decay 4 -8923.2 412.0 0.00

Distance to nearest stream Linear 4 -8925.2 416.0 0.00

Distance to wet meadow Expon. decay 4 -8927.5 420.5 0.00

Distance to intermittent stream Linear 4 -8931.3 428.2 0.00

Distance to wet meadow Linear 4 -8931.9 429.4 0.00

Distance to nearest stream Expon. decay 4 -8932.0 429.6 0.00

Null Null 3 -8934.7 432.9 0.00

Distance to intermittent stream Expon. decay 4 -8933.7 433.0 0.00

Topography Roughness index* 1 ha 4 -8409.9 0.0 1.00

Elevation Linear 4 -8801.9 784.0 0.00

Topographic position index 510 m 4 -8920.8 1021.7 0.00

Topographic position index 2010 m 4 -8931.5 1043.1 0.00

Null 3 -8934.7 1047.5 0.00

Summer Land cover Annual grass 661.4 ha 4 -10184.2 0.0 1.00

61.5 ha 4 -10224.1 79.8 0.00

8.7 ha 4 -10235.7 103.0 0.00

Null 3 -10237.8 105.2 0.00

Bare ground 661.4 ha 4 -9436.9 0.0 1.00

61.5 ha 4 -9551.3 228.9 0.00

8.7 ha 4 -9656.5 439.2 0.00

Null 3 -10237.8 1599.8 0.00

Big sagebrush 661.4 ha 4 -9993.4 0.0 1.00

61.5 ha 4 -10095.8 204.9 0.00

8.7 ha 4 -10109.9 233.1 0.00

Null 3 -10237.8 486.8 0.00

Cropland 661.4 ha 4 -10131.6 0.0 1.00

61.5 ha 4 -10229.6 196.1 0.00

8.7 ha 4 -10235.7 208.2 0.00

Null 3 -10237.8 210.4 0.00

Forest 661.4 ha 4 -10035.0 0.0 1.00

61.5 ha 4 -10068.8 67.6 0.00

8.7 ha 4 -10107.0 144.1 0.00

Null 3 -10237.8 403.6 0.00

Herbaceous 61.5 ha 4 -9887.0 0.0 1.00

8.7 ha 4 -9925.8 77.4 0.00

661.4 ha 4 -9948.6 123.1 0.00

Null 3 -10237.8 699.5 0.00

Non-sagebrush shrub Null 3 -10237.8 0.0 0.46

8.7 ha 4 -10237.7 1.8 0.19

61.5 ha 4 -10237.7 1.9 0.18

661.4 ha 4 -10237.8 1.9 0.17

Other sagebrush 661.4 ha 4 -10231.9 0.0 0.99

Null 3 -10237.8 9.8 0.01

61.5 ha 4 -10237.4 11.1 0.00

8.7 ha 4 -10237.6 11.4 0.00

Riparian 661.4 ha 4 -9964.1 0.0 1.00

61.5 ha 4 -10108.9 289.5 0.00

8.7 ha 4 -10171.1 414.1 0.00

Null 3 -10237.8 545.4 0.00

Land cover Wet meadow 661.4 ha 4 -10209.1 0.0 1.00

61.5 ha 4 -10226.7 35.2 0.00

8.7 ha 4 -10234.4 50.5 0.00

Null 3 -10237.8 55.3 0.00

Sagebrush height Sagebrush height 61.5 ha 4 -10152.3 0.0 1.00

661.4 ha 4 -10171.6 38.5 0.00

8.7 ha 4 -10234.5 164.3 0.00

Null 3 -10237.8 168.9 0.00

Agriculture Distance to cropland Expon. decay 4 -10219.1 0.0 1.00

Null 3 -10237.8 35.4 0.00

Linear 4 -10236.8 35.4 0.00

Edge Variety of edge types 61.5 ha 4 -9857.7 0.0 1.00

661.4 ha 4 -9876.6 37.9 0.00

8.7 ha 4 -9897.8 80.3 0.00

Null 3 -10237.8 758.2 0.00

Landscape variation Variety of land cover types 61.5 ha 4 -9774.3 0.0 1.00

8.7 ha 4 -9855.0 161.5 0.00

661.4 ha 4 -10054.3 560.1 0.00

Null 3 -10237.8 925.0 0.00

Water sources Distance to spring Expon. decay 4 -8869.7 0.0 1.00

Distance to spring Linear 4 -8908.6 77.8 0.00

Distance to perennial stream Expon. decay 4 -9760.9 1782.5 0.00

Distance to intermittent stream Expon. decay 4 -9778.3 1817.2 0.00

Distance to water body Linear 4 -9826.2 1913.1 0.00

Distance to intermittent stream Linear 4 -9846.2 1953.0 0.00

Distance to perennial stream Linear 4 -9884.4 2029.4 0.00

Distance to water body Expon. decay 4 -10055.7 2372.1 0.00

Distance to nearest stream Expon. decay 4 -10102.9 2466.3 0.00

Distance to nearest stream Linear 4 -10149.0 2558.6 0.00

Distance wet meadow Linear 4 -10228.6 2717.8 0.00

Null Null 3 -10237.8 2734.2 0.00

Distance to wet meadow Expon. decay 4 -10237.5 2735.6 0.00

Topography Roughness index 1 ha 4 -8222.9 0.0 1.00

Elevation Linear 4 -9926.6 3407.3 0.00

Topographic position index 2010 m 4 -10228.2 4010.5 0.00

Null 3 -10237.8 4027.7 0.00

Topographic position index 510 m 4 -10237.3 4028.7 0.00

Winter Land cover Annual grass 661.4 ha 4 -2415.8 0.0 0.97

61.5 ha 4 -2419.5 7.3 0.02

8.7 ha 4 -2422.3 13.0 0.00

Null 3 -2426.0 18.4 0.00

Bare ground 8.7 ha 4 -2422.1 0.0 0.89

Null 3 -2426.0 5.9 0.05

61.5 ha 4 -2425.1 6.1 0.04

661.4 ha 4 -2425.7 7.2 0.02

Big sagebrush 661.4 ha 4 -2355.3 0.0 1.00

61.5 ha 4 -2406.9 103.3 0.00

8.7 ha 4 -2415.9 121.2 0.00

Null 3 -2426.0 139.5 0.00

Cropland 661.4 ha 4 -2390.7 0.0 1.00

61.5 ha 4 -2420.2 59.0 0.00

Null 3 -2426.0 68.6 0.00

8.7 ha 4 -2425.7 70.0 0.00

Herbaceous 8.7 ha 4 -2419.6 0.0 0.98

661.4 ha 4 -2424.4 9.7 0.01

61.5 ha 4 -2424.8 10.4 0.01

Null 3 -2426.0 10.9 0.00

Non-sagebrush shrub 661.4 ha 4 -2315.7 0.0 1.00

61.5 ha 4 -2373.1 114.8 0.00

8.7 ha 4 -2400.9 170.4 0.00

Null 3 -2426.0 218.6 0.00

Other sagebrush 661.4 ha 4 -2416.6 0.0 1.00

Null 3 -2426.0 16.8 0.00

61.5 ha 4 -2425.4 17.5 0.00

8.7 ha 4 -2425.8 18.4 0.00

Sagebrush height Sagebrush height Null 3 -2426.0 0.0 0.36

661.4 ha 4 -2425.3 0.5 0.27

8.7 ha 4 -2425.5 0.9 0.23

61.5 ha 4 -2425.9 1.8 0.14

Agriculture Distance to cropland Linear 4 -2417.6 0.0 1.00

Expon. Decay 4 -2424.2 13.2 0.00

Null 3 -2426.0 14.9 0.00

Edge Variety of edge types 61.5 ha 4 -2336.6 0.0 0.95

8.7 ha 4 -2339.5 5.8 0.05

661.4 ha 4 -2354.4 35.6 0.00

Null 3 -2426.0 176.9 0.00

Landscape variation Variety of land cover types 61.5 ha 4 -2254.0 0.0 1.00

8.7 ha 4 -2319.8 131.6 0.00

661.4 ha 4 -2349.8 191.7 0.00

Null 3 -2426.0 342.1 0.00

Water sources Distance to spring Expon. decay 4 -2321.4 0.0 0.96

Distance to spring Linear 4 -2324.7 6.6 0.04

Distance to perennial stream Expon. decay 4 -2326.8 10.8 0.00

Distance to perennial stream Linear 4 -2376.0 109.2 0.00

Distance to intermittent stream Expon. decay 4 -2416.5 190.2 0.00

Distance to intermittent stream Linear 4 -2421.7 200.6 0.00

Distance to nearest stream Expon. decay 4 -2422.5 202.1 0.00

Distance to nearest stream Linear 4 -2424.3 205.7 0.00

Distance to water body Expon. decay 4 -2424.9 206.9 0.00

Null Null 3 -2426.0 207.2 0.00

Distance to wet meadow Expon. decay 4 -2425.1 207.3 0.00

Distance to water body Linear 4 -2425.9 208.8 0.00

Distance to wet meadow Linear 4 -2426.0 209.1 0.00

Topography Roughness index 1 ha 4 -2226.6 0.0 1.00

Elevation Linear 4 -2421.9 390.6 0.00

Topographic position index 510 m 4 -2422.9 392.6 0.00

Null 3 -2426.0 396.9 0.00

Topographic position index 2010 m 4 -2426.0 398.9 0.00

Cortez Spring Land cover Annual grass 8.7 ha 4 -22460.3 0.0 1.00

61.5 ha 4 -22470.3 19.9 0.00

661.4 ha 4 -22476.3 32.0 0.00

Null 3 -22478.8 34.9 0.00

Bare ground 8.7 ha 4 -21898.1 0.0 1.00

61.5 ha 4 -21911.0 25.9 0.00

661.4 ha 4 -22043.7 291.3 0.00

Null 3 -22478.8 1159.4 0.00

Big sagebrush 661.4 ha 4 -21933.3 0.0 1.00

61.5 ha 4 -21974.0 81.4 0.00

8.7 ha 4 -22069.0 271.4 0.00

Null 3 -22478.8 1088.9 0.00

Cropland 661.4 ha 4 -22101.3 0.0 1.00

61.5 ha 4 -22227.3 251.9 0.00

8.7 ha 4 -22258.4 314.2 0.00

Null 3 -22478.8 752.9 0.00

Herbaceous 8.7 ha 4 -22365.0 0.0 0.96

661.4 ha 4 -22368.3 6.5 0.04

61.5 ha 4 -22372.0 14.0 0.00

Null 3 -22478.8 225.5 0.00

Non-sagebrush shrub 661.4 ha 4 -22455.3 0.0 0.90

8.7 ha 4 -22458.1 5.8 0.05

61.5 ha 4 -22458.2 5.9 0.05

Null 3 -22478.8 45.0 0.00

Other sagebrush 661.4 ha 4 -20183.6 0.0 1.00

61.5 ha 4 -20427.4 487.6 0.00

8.7 ha 4 -20748.2 1129.2 0.00

Null 3 -22478.8 4588.4 0.00

Pinyon-juniper 8.7 ha 4 -21444.5 0.0 1.00

61.5 ha 4 -21485.4 81.8 0.00

661.4 ha 4 -21910.2 931.3 0.00

Null 3 -22478.8 2066.5 0.00

Riparian 661.4 ha 4 -21914.3 0.0 1.00

61.5 ha 4 -22111.0 393.4 0.00

8.7 ha 4 -22215.3 602.0 0.00

Null 3 -22478.8 1126.9 0.00

Sagebrush height Sagebrush height 661.4 ha 4 -22470.6 0.0 0.88

61.5 ha 4 -22472.6 4.0 0.12

Null 3 -22478.8 14.4 0.00

8.7 ha 4 -22477.8 14.4 0.00

Agriculture Distance to cropland Expon. decay 4 -22093.6 0.0 1.00

Linear 4 -22264.2 341.1 0.00

Null 3 -22478.8 768.3 0.00

Edge Variety of edge types 8.7 ha 4 -22340.8 0.0 1.00

61.5 ha 4 -22371.8 61.9 0.00

661.4 ha 4 -22419.2 156.9 0.00

Null 3 -22478.8 273.9 0.00

Landscape variation Variety of land cover types 61.5 ha 4 -22221.6 0.0 1.00

8.7 ha 4 -22227.6 12.0 0.00

661.4 ha 4 -22443.3 443.5 0.00

Null 3 -22478.8 512.4 0.00

Water sources Distance to intermittent stream Linear 4 -22147.6 0.0 1.00

Distance to perennial stream Linear 4 -22183.2 71.2 0.00

Distance to perennial stream Expon. decay 4 -22229.5 163.8 0.00

Distance to nearest stream Linear 4 -22247.4 199.6 0.00

Distance to water body Expon. decay 4 -22302.9 310.6 0.00

Distance to water body Linear 4 -22371.0 446.9 0.00

Distance to spring Linear 4 -22398.0 500.9 0.00

Distance to intermittent stream Expon. decay 4 -22402.8 510.4 0.00

Distance to nearest stream Expon. decay 4 -22411.1 527.0 0.00

Distance to spring Expon. decay 4 -22430.3 565.4 0.00

Distance to wet meadow Expon. decay 4 -22453.8 612.4 0.00

Distance to wet meadow Linear 4 -22476.9 658.7 0.00

Null Null 3 -22478.8 660.4 0.00

Topography Topographic position index 510 m 4 -22292.3 0.0 1.00

Elevation Linear 4 -22367.7 150.7 0.00

Roughness index 1 ha 4 -22469.8 355.0 0.00

Topographic position index 2010 m 4 -22476.8 369.1 0.00

Null 3 -22478.8 370.9 0.00

Summer Land cover Annual grass 661.4 ha 4 -4439.3 0.0 1.00

61.5 ha 4 -4514.0 149.4 0.00

8.7 ha 4 -4539.3 200.1 0.00

Null 3 -4567.8 255.2 0.00

Bare ground 661.4 ha 4 -4409.7 0.0 1.00

61.5 ha 4 -4442.4 65.3 0.00

8.7 ha 4 -4446.7 74.0 0.00

Null 3 -4567.8 314.2 0.00

Big sagebrush 661.4 ha 4 -3738.5 0.0 1.00

61.5 ha 4 -3905.7 334.5 0.00

8.7 ha 4 -3915.8 354.7 0.00

Null 3 -4567.8 1656.8 0.00

Cropland 661.4 ha 4 -4532.2 0.0 1.00

61.5 ha 4 -4560.0 55.7 0.00

8.7 ha 4 -4564.6 64.9 0.00

Null 3 -4567.8 69.4 0.00

Forest 61.5 ha 4 -4563.1 0.0 0.64

8.7 ha 4 -4564.1 2.1 0.22

661.4 ha 4 -4564.7 3.2 0.13

Null 3 -4567.8 7.5 0.01

Herbaceous 661.4 ha 4 -3468.7 0.0 1.00

61.5 ha 4 -3640.0 342.6 0.00

8.7 ha 4 -3767.5 597.6 0.00

Null 3 -4567.8 2196.2 0.00

Non-sagebrush shrub 661.4 ha* 4 -4234.4 0.0 1.00

61.5 ha 4 -4286.8 104.9 0.00

8.7 ha 4 -4314.9 160.9 0.00

Null 3 -4567.8 664.8 0.00

Other sagebrush 661.4 ha 4 -3782.7 0.0 1.00

61.5 ha 4 -4028.5 491.6 0.00

8.7 ha 4 -4182.2 798.9 0.00

Null 3 -4567.8 1568.3 0.00

Pinyon-juniper 61.5 ha 4 -4463.0 0.0 0.90

8.7 ha 4 -4465.2 4.3 0.10

661.4 ha 4 -4496.2 66.2 0.00

Null 3 -4567.8 207.6 0.00

Land cover Riparian 661.4 ha 4 -4544.7 0.0 1.00

61.5 ha 4 -4563.8 38.2 0.00

Null 3 -4567.8 44.2 0.00

8.7 ha 4 -4567.6 45.7 0.00

Sagebrush height Sagebrush height 8.7 ha 4 -4532.1 0.0 1.00

661.4 ha 4 -4546.8 29.5 0.00

61.5 ha 4 -4551.9 39.7 0.00

Null 3 -4567.8 69.6 0.00

Agriculture Distance to cropland Linear 4 -4520.1 0.0 1.00

Expon. decay 4 -4539.5 38.6 0.00

Null 3 -4567.8 93.4 0.00

Edge Variety of edge types 661.4 ha* 4 -3872.0 0.0 1.00

61.5 ha 4 -4282.1 820.3 0.00

8.7 ha 4 -4386.9 1029.9 0.00

Null 3 -4567.8 1389.7 0.00

Landscape Variation Variety of land cover types 661.4 ha 4 -4093.7 0.0 1.00

8.7 ha 4 -4424.5 661.6 0.00

61.5 ha 4 -4429.3 671.2 0.00

Null 3 -4567.8 946.2 0.00

Water sources Distance to spring Expon. decay 4 -3511.7 0.0 1.00

Distance to spring Linear 4 -3729.8 436.3 0.00

Distance to perennial stream Expon. decay 4 -4045.7 1067.9 0.00

Distance to perennial stream Linear 4 -4062.1 1100.7 0.00

Distance to nearest stream Linear 4 -4357.4 1691.3 0.00

Distance to nearest stream Expon. decay 4 -4452.7 1882.0 0.00

Distance to intermittent stream Linear 4 -4471.1 1918.8 0.00

Distance to water body Linear 4 -4487.3 1951.2 0.00

Distance to wet meadow Expon. decay 4 -4506.6 1989.7 0.00

Distance to intermittent stream Expon. decay 4 -4516.0 2008.5 0.00

Distance to water body Expon. decay 4 -4518.1 2012.9 0.00

Distance to wet meadow Linear 4 -4533.3 2043.2 0.00

Null Null 3 -4567.8 2110.3 0.00

Topography Elevation Linear 4 -3599.0 0.0 1.00

Roughness index 1 ha 4 -4224.6 1251.1 0.00

Topographic position index 510 m 4 -4565.1 1932.1 0.00

Null 3 -4567.8 1935.6 0.00

Topographic position index 2010 m 4 -4567.3 1936.5 0.00

Winter Land cover Annual grass 661.4 ha 4 -2050.9 0.0 1.00

61.5 ha 4 -2057.5 13.3 0.00

8.7 ha 4 -2058.2 14.7 0.00

Null 3 -2065.6 27.4 0.00

Bare ground 8.7 ha 4 -2048.6 0.0 0.95

61.5 ha 4 -2051.5 5.7 0.05

661.4 ha 4 -2056.4 15.6 0.00

Null 3 -2065.6 31.9 0.00

Big sagebrush 661.4 ha 4 -2029.5 0.0 1.00

61.5 ha 4 -2040.2 21.4 0.00

8.7 ha 4 -2046.0 33.0 0.00

Null 3 -2065.6 70.2 0.00

Cropland 61.5 ha 4 -2033.8 0.0 1.00

8.7 ha 4 -2042.4 17.3 0.00

661.4 ha 4 -2044.9 22.2 0.00

Null 3 -2065.6 61.6 0.00

Herbaceous 661.4 ha 4 -2039.3 0.0 1.00

61.5 ha 4 -2049.1 19.7 0.00

8.7 ha 4 -2050.3 22.0 0.00

Null 3 -2065.6 50.5 0.00

Non-sagebrush shrub 661.4 ha 4 -2032.8 0.0 1.00

61.5 ha 4 -2044.5 23.3 0.00

8.7 ha 4 -2044.7 23.8 0.00

Null 3 -2065.6 63.5 0.00

Other sagebrush 61.5 ha 4 -1970.5 0.0 1.00

661.4 ha 4 -1976.5 11.9 0.00

8.7 ha 4 -1977.3 13.4 0.00

Null 3 -2065.6 188.1 0.00

Pinyon-juniper 61.5 ha 4 -2049.5 0.0 0.49

8.7 ha 4 -2049.6 0.1 0.48

661.4 ha 4 -2052.3 5.6 0.03

Null 3 -2065.6 30.1 0.00

Riparian 661.4 ha 4 -2038.6 0.0 1.00

8.7 ha 4 -2047.1 17.0 0.00

61.5 ha 4 -2048.3 19.5 0.00

Null 3 -2065.6 52.0 0.00

Sagebrush height Sagebrush height Null 3 -2065.6 0.0 0.33

61.5 ha 4 -2064.7 0.3 0.29

661.4 ha 4 -2064.8 0.5 0.26

8.7 ha 4 -2065.6 2.0 0.13

Agriculture Distance to cropland Expon. decay 4 -2063.7 0.0 0.64

Null 3 -2065.6 1.8 0.26

Linear 4 -2065.6 3.8 0.10

Edge Variety of edge types 8.7 ha 4 -2060.5 0.0 0.95

661.4 ha 4 -2064.5 8.0 0.02

Null 3 -2065.6 8.2 0.02

61.5 ha 4 -2064.7 8.5 0.01

Landscape variation Variety of land cover types 8.7 ha 4 -2050.6 0.0 0.95

61.5 ha 4 -2053.6 5.9 0.05

661.4 ha 4 -2060.9 20.6 0.00

Null 3 -2065.6 27.9 0.00

Water sources Distance to perennial stream Linear 4 -2003.3 0.0 1.00

Distance to perennial stream Expon. decay 4 -2014.2 21.7 0.00

Distance to nearest stream Linear 4 -2028.1 49.6 0.00

Distance to intermittent stream Linear 4 -2032.4 58.1 0.00

Distance to spring Expon. decay 4 -2042.4 78.1 0.00

Distance to spring Linear 4 -2047.1 87.5 0.00

Distance to nearest stream Expon. decay 4 -2053.5 100.5 0.00

Distance to intermittent stream Expon. decay 4 -2058.8 110.9 0.00

Distance to water body Linear 4 -2059.3 112.0 0.00

Distance to wet meadow Expon. decay 4 -2062.6 118.5 0.00

Distance to water body Expon. decay 4 -2064.1 121.6 0.00

Null Null 3 -2065.6 122.5 0.00

Distance to wet meadow Linear 4 -2064.9 123.2 0.00

Topography Elevation Linear 4 -2035.9 0.0 1.00

Roughness index 1 ha 4 -2055.4 38.9 0.00

Topographic position index 510 m 4 -2063.0 54.2 0.00

Null 3 -2065.6 57.3 0.00

Topographic position index 2010 m 4 -2065.1 58.4 0.00

Gollaher Spring Land cover Big sagebrush 661.4 ha 4 -7584.8 0.0 1.00

61.5 ha 4 -7697.3 225.1 0.00

8.7 ha 4 -7769.6 369.6 0.00

Null 3 -8234.6 1297.7 0.00

Cropland 661.4 ha 4 -7929.3 0.0 1.00

61.5 ha 4 -8070.8 283.1 0.00

8.7 ha 4 -8118.5 378.5 0.00

Null 3 -8234.6 608.6 0.00

Forest 661.4 ha 4 -7109.6 0.0 1.00

61.5 ha 4 -7546.4 873.6 0.00

8.7 ha 4 -7842.1 1465.1 0.00

Null 3 -8234.6 2248.0 0.00

Herbaceous 661.4 ha 4 -7796.9 0.0 1.00

61.5 ha 4 -7857.3 120.9 0.00

8.7 ha 4 -7906.6 219.4 0.00

Null 3 -8234.6 873.4 0.00

Non-sagebrush shrub 661.4 ha 4 -6354.1 0.0 1.00

61.5 ha 4 -6493.9 279.7 0.00

8.7 ha 4 -6809.7 911.2 0.00

Null 3 -8234.6 3759.0 0.00

Other sagebrush 661.4 ha 4 -6403.6 0.0 1.00

61.5 ha 4 -6648.7 490.1 0.00

8.7 ha 4 -7012.0 1216.8 0.00

Null 3 -8234.6 3659.9 0.00

Riparian 61.5 ha 4 -7675.7 0.0 1.00

661.4 ha 4 -7700.8 50.2 0.00

8.7 ha 4 -7891.1 430.7 0.00

Null 3 -8234.6 1115.7 0.00

Sagebrush height Sagebrush height 661.4 ha 4 -8049.3 0.0 1.00

61.5 ha 4 -8092.5 86.3 0.00

8.7 ha 4 -8118.8 138.9 0.00

Null 3 -8234.6 368.6 0.00

Agriculture Distance to cropland Linear 4 -7535.9 0.0 1.00

Expon. decay 4 -8067.8 1064.0 0.00

Null 3 -8234.6 1395.5 0.00

Edge Variety of edge types 661.4 ha 4 -7079.4 0.0 1.00

61.5 ha 4 -7223.1 287.5 0.00

8.7 ha 4 -7521.0 883.3 0.00

Null 3 -8234.6 2308.4 0.00

Landscape variation Variety of land cover types 61.5 ha 4 -7253.4 0.0 1.00

8.7 ha 4 -7391.1 275.5 0.00

661.4 ha 4 -7767.7 1028.8 0.00

Null 3 -8234.6 1960.5 0.00

Water sources Distance to spring Expon. decay 4 -7805.0 0.0 1.00

Distance to spring Linear 4 -7863.2 116.3 0.00

Distance to intermittent stream Linear 4 -7870.1 130.1 0.00

Distance to intermittent stream Expon. decay 4 -8029.5 448.9 0.00

Distance to water body Expon. decay 4 -8066.1 522.0 0.00

Distance to nearest stream Linear 4 -8155.5 700.8 0.00

Distance to nearest stream Expon. decay 4 -8164.3 718.6 0.00

Distance to water body Linear 4 -8186.4 762.7 0.00

Distance to perennial stream Linear 4 -8220.4 830.7 0.00

Null Null 3 -8234.6 857.1 0.00

Distance to perennial stream Expon. decay 4 -8233.8 857.4 0.00

Topography Elevation Linear 4 -6884.0 0.0 1.00

Roughness index 1 ha 4 -8070.3 2372.6 0.00

Topographic position index 2010 m 4 -8197.2 2626.5 0.00

Topographic position index 510 m 4 -8218.8 2669.5 0.00

Null 3 -8234.6 2699.2 0.00

Summer Land cover Big sagebrush 661.4 ha 4 -4772.4 0.0 0.85

Null 3 -4775.8 4.7 0.08

61.5 ha 4 -4775.5 6.2 0.04

8.7 ha 4 -4775.7 6.5 0.03

Cropland 661.4 ha 4 -4724.3 0.0 1.00

61.5 ha 4 -4763.7 78.9 0.00

8.7 ha 4 -4766.3 84.0 0.00

Null 3 -4775.8 101.0 0.00

Forest 661.4 ha 4 -4690.5 0.0 1.00

61.5 ha 4 -4726.6 72.1 0.00

8.7 ha 4 -4746.3 111.5 0.00

Null 3 -4775.8 168.5 0.00

Herbaceous 661.4 ha 4 -4678.9 0.0 1.00

8.7 ha 4 -4694.3 30.9 0.00

61.5 ha 4 -4696.8 35.9 0.00

Null 3 -4775.8 191.8 0.00

Non-sagebrush shrub 661.4 ha 4 -4747.4 0.0 1.00

61.5 ha 4 -4759.1 23.4 0.00

8.7 ha 4 -4769.8 44.8 0.00

Null 3 -4775.8 54.8 0.00

Other sagebrush 661.4 ha 4 -4707.3 0.0 1.00

61.5 ha 4 -4739.8 65.0 0.00

8.7 ha 4 -4767.9 121.3 0.00

Null 3 -4775.8 135.0 0.00

Riparian 661.4 ha 4 -4670.3 0.0 1.00

61.5 ha 4 -4733.3 126.0 0.00

8.7 ha 4 -4757.7 174.7 0.00

Null 3 -4775.8 208.9 0.00

Sagebrush height Sagebrush height 61.5 ha 4 -4773.2 0.0 0.62

8.7 ha 4 -4774.3 2.2 0.21

Null 3 -4775.8 3.2 0.13

661.4 ha 4 -4775.7 5.0 0.05

Agriculture Distance to cropland Linear 4 -4552.6 0.0 1.00

Expon. decay 4 -4622.0 138.7 0.00

Null 3 -4775.8 444.3 0.00

Edge Variety of edge types 661.4 ha 4 -4771.0 0.0 0.71

61.5 ha 4 -4772.0 2.0 0.27

Null 3 -4775.8 7.5 0.02

8.7 ha 4 -4775.7 9.3 0.01

Landscape variation Variety of land cover types 661.4 ha 4 -4742.2 0.0 1.00

61.5 ha 4 -4772.9 61.4 0.00

Null 3 -4775.8 65.2 0.00

8.7 ha 4 -4775.6 66.7 0.00

Water sources Distance to spring Linear 4 -4484.9 0.0 1.00

Distance to spring Expon. decay 4 -4635.1 300.4 0.00

Distance to water body Linear 4 -4656.5 343.2 0.00

Distance to water body Expon. decay 4 -4678.7 387.6 0.00

Distance to intermittent stream Expon. decay 4 -4733.8 497.9 0.00

Distance to nearest stream Linear 4 -4746.9 524.1 0.00

Distance to intermittent stream Linear 4 -4748.0 526.2 0.00

Distance to nearest stream Expon. decay 4 -4748.6 527.4 0.00

Distance to perennial stream Linear 4 -4759.0 548.3 0.00

Distance to perennial stream Expon. decay 4 -4774.3 579.0 0.00

Null Null 3 -4775.8 579.9 0.00

Topography Roughness index 1 ha 4 -4707.3 0.0 1.00

Topographic position index 2010 m 4 -4726.1 37.7 0.00

Elevation Linear 4 -4761.6 108.6 0.00

Topographic position index 510 m 4 -4766.5 118.3 0.00

Null 3 -4775.8 135.0 0.00

Winter Land cover Big sagebrush 661.4 ha 4 -1350.9 0.0 1.00

61.5 ha 4 -1375.0 48.1 0.00

8.7 ha 4 -1378.1 54.2 0.00

Null 3 -1427.9 151.9 0.00

Cropland 661.4 ha 4 -1409.9 0.0 1.00

61.5 ha 4 -1417.8 15.8 0.00

8.7 ha 4 -1419.8 19.8 0.00

Null 3 -1427.9 34.0 0.00

Herbaceous 61.5 ha 4 -1253.9 0.0 1.00

661.4 ha 4 -1262.6 17.5 0.00

8.7 ha 4 -1278.2 48.8 0.00

Null 3 -1427.9 346.0 0.00

Non-sagebrush shrub 661.4 ha 4 -1132.0 0.0 1.00

61.5 ha 4 -1139.5 15.0 0.00

8.7 ha 4 -1185.0 106.0 0.00

Null 3 -1427.9 589.7 0.00

Other sagebrush 661.4 ha 4 -876.5 0.0 1.00

61.5 ha 4 -990.0 227.1 0.00

8.7 ha 4 -1104.2 455.5 0.00

Null 3 -1427.9 1100.8 0.00

Forest 661.4 ha 4 -1321.1 0.0 1.00

61.5 ha 4 -1342.4 42.7 0.00

8.7 ha 4 -1368.8 95.5 0.00

Null 3 -1427.9 211.6 0.00

Riparian 61.5 ha 4 -1381.0 0.0 0.91

8.7 ha 4 -1383.5 4.8 0.08

661.4 ha 4 -1385.4 8.8 0.01

Null 3 -1427.9 91.7 0.00

Sagebrush height Sagebrush height 661.4 ha 4 -1421.8 0.0 0.92

8.7 ha 4 -1424.5 5.5 0.06

61.5 ha 4 -1426.0 8.4 0.01

Null 3 -1427.9 10.2 0.01

Agriculture Distance to cropland Linear 4 -1315.4 0.0 1.00

Expon. decay 4 -1389.8 148.6 0.00

Null 3 -1427.9 222.9 0.00

Edge Variety of edge types 8.7 ha 4 -1364.4 0.0 1.00

61.5 ha 4 -1378.6 28.4 0.00

661.4 ha 4 -1397.8 66.9 0.00

Null 3 -1427.9 125.0 0.00

Landscape variation Variety of land cover types 8.7 ha 4 -1344.6 0.0 1.00

61.5 ha 4 -1366.3 43.4 0.00

661.4 ha 4 -1425.8 162.4 0.00

Null 3 -1427.9 164.5 0.00

Water sources Distance to intermittent stream Expon. decay 4 -1197.1 0.0 0.93

Distance to intermittent stream Linear 4 -1199.7 5.2 0.07

Distance to nearest stream Linear 4 -1246.1 97.8 0.00

Distance to nearest stream Expon. decay 4 -1265.4 136.6 0.00

Distance to spring Expon. decay 4 -1365.1 336.0 0.00

Distance to water body Linear 4 -1391.4 388.5 0.00

Distance to spring Linear 4 -1399.3 404.4 0.00

Distance to water body Expon. decay 4 -1409.3 424.3 0.00

Distance to perennial stream Linear 4 -1426.7 459.2 0.00

Null Null 3 -1427.9 459.5 0.00

Distance to perennial stream Expon. decay 4 -1427.5 460.7 0.00

Topography Elevation Linear 4 -1286.3 0.0 1.00

Roughness index 1 ha 4 -1360.4 148.2 0.00

Topographic position index 2010 m 4 -1423.1 273.6 0.00

Null 3 -1427.9 281.2 0.00

Topographic position index 510 m 4 -1427.6 282.7 0.00

Lincoln Spring Land cover Bare ground 661.4 ha 4 -1967.0 0.0 1.00

8.7 ha 4 -1988.8 43.6 0.00

61.5 ha 4 -1990.5 46.9 0.00

Null 3 -2010.1 84.2 0.00

Big sagebrush 661.4 ha 4 -1947.9 0.0 0.98

8.7 ha 4 -1952.1 8.4 0.01

61.5 ha 4 -1954.1 12.3 0.00

Null 3 -2010.1 122.4 0.00

Cropland 661.4 ha 4 -1769.5 0.0 1.00

61.5 ha 4 -1833.6 128.1 0.00

8.7 ha 4 -1856.8 174.5 0.00

Null 3 -2010.1 479.2 0.00

Forest 8.7 ha 4 -1876.2 0.0 1.00

61.5 ha 4 -1891.1 29.8 0.00

661.4 ha 4 -1896.9 41.5 0.00

Null 3 -2010.1 265.9 0.00

Herbaceous 661.4 ha 4 -1934.3 0.0 1.00

8.7 ha 4 -1967.4 66.2 0.00

61.5 ha 4 -1970.1 71.5 0.00

Null 3 -2010.1 149.6 0.00

Non-sagebrush shrub 61.5 ha 4 -2002.0 0.0 0.96

8.7 ha 4 -2005.3 6.5 0.04

661.4 ha 4 -2008.9 13.7 0.00

Null 3 -2010.1 14.2 0.00

Other sagebrush 661.4 ha 4 -1947.5 0.0 1.00

8.7 ha 4 -1953.2 11.3 0.00

61.5 ha 4 -1965.4 35.7 0.00

Null 3 -2010.1 123.2 0.00

Pinyon-juniper 61.5 ha 4 -1596.1 0.0 0.99

8.7 ha 4 -1600.4 8.6 0.01

661.4 ha 4 -1628.0 63.8 0.00

Null 3 -2010.1 826.0 0.00

Riparian 8.7 ha 4 -1992.3 0.0 1.00

Null 3 -2010.1 33.7 0.00

61.5 ha 4 -2009.7 34.7 0.00

661.4 ha 4 -2010.1 35.7 0.00

Sagebrush height Sagebrush height Null 3 -2010.1 0.0 0.37

661.4 ha 4 -2009.2 0.1 0.35

61.5 ha 4 -2010.0 1.8 0.15

8.7 ha 4 -2010.1 2.0 0.14

Agriculture Distance to cropland Linear 4 -1627.2 0.0 1.00

Expon. decay 4 -1688.5 122.6 0.00

Null 3 -2010.1 763.9 0.00

Edge Variety of edge types 661.4 ha 4 -1900.4 0.0 1.00

61.5 ha 4 -1967.5 134.3 0.00

8.7 ha 4 -1998.5 196.2 0.00

Null 3 -2010.1 217.5 0.00

Land cover variation Variety of land cover types 661.4 ha 4 -1875.4 0.0 1.00

61.5 ha 4 -1988.8 226.8 0.00

8.7 ha 4 -1994.2 237.5 0.00

Null 3 -2010.1 267.4 0.00

Water sources Distance to water body Linear 4 -1643.0 0.0 1.00

Distance to water body Expon. decay 4 -1693.9 102.0 0.00

Distance to wet meadow Expon. decay 4 -1749.4 212.8 0.00

Distance to wet meadow Linear 4 -1804.1 322.2 0.00

Distance to perennial stream Linear 4 -1890.7 495.6 0.00

Distance to perennial stream Expon. decay 4 -1926.0 566.2 0.00

Distance to spring Linear 4 -1932.4 578.8 0.00

Distance to spring Expon. decay 4 -1974.5 663.1 0.00

Distance to nearest stream Linear 4 -2008.5 731.0 0.00

Distance to intermittent stream Expon. decay 4 -2008.7 731.5 0.00

Null Null 3 -2010.1 732.3 0.00

Distance to intermittent stream Linear 4 -2009.7 733.5 0.00

Distance to nearest stream Expon. decay 4 -2009.9 733.8 0.00

Topography Roughness index 1 ha 4 -1806.6 0.0 1.00

Elevation Linear 4 -1879.8 146.4 0.00

Topographic position index 2010 m 4 -2005.2 397.2 0.00

Topographic position index 510 m 4 -2006.8 400.5 0.00

Null 3 -2010.1 405.1 0.00

Summer Land cover Bare ground 61.5 ha 4 -2526.3 0.0 1.00

8.7 ha 4 -2532.6 12.6 0.00

661.5 ha 4 -2588.9 125.3 0.00

Null 3 -2634.0 213.3 0.00

Big sagebrush 661.4 ha 4 -2576.4 0.0 1.00

61.5 ha 4 -2583.1 13.3 0.00

8.7 ha 4 -2589.8 26.8 0.00

Null 3 -2634.0 113.0 0.00

Cropland 661.4 ha 4 -1967.1 0.0 1.00

61.5 ha 4 -2068.6 202.9 0.00

8.7 ha 4 -2145.4 356.6 0.00

Null 3 -2634.0 1331.7 0.00

Forest 8.7 ha 4 -2564.8 0.0 0.67

61.5 ha 4 -2565.5 1.4 0.33

661.4 ha 4 -2603.2 76.7 0.00

Null 3 -2634.0 136.2 0.00

Herbaceous 661.4 ha 4 -2328.0 0.0 1.00

61.5 ha 4 -2391.8 127.6 0.00

8.7 ha 4 -2401.3 146.6 0.00

Null 3 -2634.0 609.9 0.00

Non-sagebrush shrub 661.4 ha 4 -2410.0 0.0 1.00

61.5 ha 4 -2458.0 96.0 0.00

8.7 ha 4 -2492.6 165.2 0.00

Null 3 -2634.0 445.9 0.00

Other sagebrush 661.4 ha 4 -2585.9 0.0 1.00

8.7 ha 4 -2607.8 43.7 0.00

61.5 ha 4 -2610.5 49.1 0.00

Null 3 -2634.0 94.1 0.00

Pinyon-juniper 61.5 ha 4 -2079.3 0.0 1.00

8.7 ha 4 -2123.5 88.5 0.00

661.4 ha 4 -2147.9 137.2 0.00

Null 3 -2634.0 1107.4 0.00

Riparian 661.4 ha 4 -2626.6 0.0 0.98

61.5 ha 4 -2630.6 8.1 0.02

Null 3 -2634.0 12.8 0.00

8.7 ha 4 -2633.6 14.0 0.00

Sagebrush Height Sagebrush Height 61.5 ha 4 -2616.8 0.0 1.00

8.7 ha 4 -2624.8 15.8 0.00

661.4 ha 4 -2626.4 19.1 0.00

Null 3 -2634.0 32.2 0.00

Agriculture Distance to cropland Linear 4 -1647.3 0.0 1.00

Expon. decay 4 -1803.5 312.5 0.00

Null 3 -2634.0 1971.3 0.00

Edge Variety of edge types 661.4 ha* 4 -1828.0 0.0 1.00

61.5 ha 4 -2317.9 979.9 0.00

8.7 ha 4 -2491.5 1327.0 0.00

Null 3 -2634.0 1610.0 0.00

Landscape variation Variety of land cover types 661.4 ha 4 -1757.2 0.0 1.00

61.5 ha 4 -2246.2 977.9 0.00

8.7 ha 4 -2460.2 1406.0 0.00

Null 3 -2634.0 1751.5 0.00

Water sources Distance to water body Linear 4 -1619.5 0.0 1.00

Distance to water body Expon. decay 4 -1741.7 244.3 0.00

Distance to spring Linear 4 -2173.6 1108.1 0.00

Distance to spring Expon. decay 4 -2307.3 1375.5 0.00

Distance to perennial stream Linear 4 -2338.2 1437.3 0.00

Distance to wet meadow Expon. decay 4 -2412.9 1586.7 0.00

Distance to wet meadow Linear 4 -2470.8 1702.5 0.00

Distance to perennial stream Expon. decay 4 -2473.0 1706.8 0.00

Distance to intermittent stream Expon. decay 4 -2571.5 1904.0 0.00

Distance to nearest stream Expon. decay 4 -2601.5 1963.9 0.00

Distance to intermittent stream Linear 4 -2617.0 1994.9 0.00

Distance to nearest stream Linear 4 -2627.9 2016.7 0.00

Null Null 3 -2634.0 2026.8 0.00

Topography Roughness index 1 ha 4 -2538.7 0.0 1.00

Topographic position index 510 m 4 -2626.4 175.5 0.00

Topographic position index 2010 m 4 -2630.3 183.4 0.00

Null 3 -2634.0 188.6 0.00

Elevation Linear 4 -2633.2 189.1 0.00

Winter Land cover Bare ground 8.7 ha 4 -1563.8 0.0 0.99

61.5 ha 4 -1568.4 9.3 0.01

661.4 ha 4 -1612.6 97.6 0.00

Null 3 -1892.3 655.0 0.00

Big sagebrush 661.4 ha 4 -1808.0 0.0 1.00

61.5 ha 4 -1833.3 50.6 0.00

8.7 ha 4 -1839.6 63.2 0.00

Null 3 -1892.3 166.7 0.00

Cropland 8.7 ha 4 -1875.3 0.0 1.00

661.4 ha 4 -1886.5 22.4 0.00

61.5 ha 4 -1888.0 25.4 0.00

Null 3 -1892.3 31.9 0.00

Forest 661.4 ha 4 -1810.7 0.0 1.00

61.5 ha 4 -1823.3 25.2 0.00

8.7 ha 4 -1836.5 51.5 0.00

Null 3 -1892.3 161.1 0.00

Herbaceous 661.4 ha 4 -1720.2 0.0 1.00

61.5 ha 4 -1753.8 67.1 0.00

8.7 ha 4 -1772.5 104.5 0.00

Null 3 -1892.3 342.2 0.00

Non-sagebrush shrub 61.5 ha 4 -1888.0 0.0 0.56

8.7 ha 4 -1888.7 1.4 0.28

661.4 ha 4 -1889.4 2.7 0.14

Null 3 -1892.3 6.6 0.02

Other sagebrush 661.4 ha 4 -1614.6 0.0 1.00

61.5 ha 4 -1696.0 162.8 0.00

8.7 ha 4 -1735.1 241.0 0.00

Null 3 -1892.3 553.3 0.00

Pinyon-juniper 661.4 ha 4 -1443.2 0.0 1.00

61.5 ha 4 -1463.2 40.0 0.00

8.7 ha 4 -1498.0 109.5 0.00

Null 3 -1892.3 896.2 0.00

Riparian 61.5 ha 4 -1736.4 0.0 1.00

661.4 ha 4 -1745.1 17.4 0.00

8.7 ha 4 -1793.4 114.0 0.00

Null 3 -1892.3 309.9 0.00

Sagebrush height Sagebrush height 661.4 ha 4 -1842.3 0.0 0.82

61.5 ha 4 -1843.8 3.0 0.18

8.7 ha 4 -1874.2 63.7 0.00

Null 3 -1892.3 97.9 0.00

Agriculture Distance to cropland Linear 4 -1786.7 0.0 1.00

Expon. decay 4 -1824.7 75.9 0.00

Null 3 -1892.3 209.2 0.00

Edge Variety of edge types 8.7 ha 4 -1840.1 0.0 1.00

61.5 ha 4 -1867.6 55.0 0.00

Null 3 -1892.3 102.5 0.00

661.4 ha 4 -1892.3 104.4 0.00

Landscape variation Variety of land cover types 8.7 ha 4 -1810.6 0.0 1.00

61.5 ha 4 -1834.8 48.4 0.00

Null 3 -1892.3 161.5 0.00

661.4 ha 4 -1891.3 161.6 0.00

Water sources Distance to water body Linear 4 -1811.4 0.0 1.00

Distance to nearest stream Linear 4 -1841.9 60.9 0.00

Distance to water body Expon. decay 4 -1844.2 65.6 0.00

Distance to intermittent stream Linear 4 -1854.7 86.6 0.00

Distance to nearest stream Expon. decay 4 -1865.6 108.4 0.00

Distance to wet meadow Linear 4 -1873.0 123.2 0.00

Distance to intermittent stream Expon. decay 4 -1873.1 123.5 0.00

Distance to wet meadow Expon. decay 4 -1875.4 128.0 0.00

Distance to perennial stream Linear 4 -1877.1 131.4 0.00

Distance to perennial stream Expon. decay 4 -1879.7 136.7 0.00

Distance to spring Expon. decay 4 -1883.4 144.1 0.00

Distance to spring Linear 4 -1889.6 156.4 0.00

Null Null 3 -1892.3 159.8 0.00

Topography Roughness index 1 ha 4 -1676.0 0.0 1.00

Elevation Linear 4 -1791.8 231.5 0.00

Topographic position index 510 m 4 -1885.0 418.1 0.00

Null 3 -1892.3 430.6 0.00

Topographic position index 2010 m 4 -1892.3 432.6 0.00

Midway Spring Land cover Bare ground 661.4 ha 4 -8910.9 0.0 1.00

61.5 ha 4 -9023.2 224.5 0.00

8.7 ha 4 -9030.3 238.8 0.00

Null 3 -9031.7 239.6 0.00

Big sagebrush 661.4 ha 4 -9020.7 0.0 1.00

61.5 ha 4 -9029.9 18.4 0.00

Null 3 -9031.7 20.0 0.00

8.7 ha 4 -9031.7 22.0 0.00

Cropland 61.5 ha 4 -8203.2 0.0 1.00

661.4 ha 4 -8270.0 133.7 0.00

8.7 ha 4 -8424.5 442.6 0.00

Null 3 -9031.7 1655.0 0.00

Forest 61.5 ha 4 -8931.7 0.0 1.00

661.4 ha 4 -8939.5 15.5 0.00

8.7 ha 4 -8981.9 100.3 0.00

Null 3 -9031.7 198.0 0.00

Herbaceous 8.7 ha 4 -8898.8 0.0 1.00

61.5 ha 4 -8918.0 38.4 0.00

661.4 ha 4 -8931.3 65.0 0.00

Null 3 -9031.7 263.8 0.00

Non-sagebrush shrub 8.7 ha 4 -8974.7 0.0 1.00

61.5 ha 4 -8994.0 38.7 0.00

661.4 ha 4 -9030.3 111.2 0.00

Null 3 -9031.7 112.1 0.00

Other sagebrush 661.4 ha* 4 -8945.3 0.0 0.96

61.5 ha 4 -8948.6 6.6 0.04

8.7 ha 4 -8965.8 40.9 0.00

Null 3 -9031.7 170.8 0.00

Pinyon-juniper 61.5 ha 4 -7427.7 0.0 1.00

8.7 ha 4 -7471.5 87.6 0.00

661.5 ha 4 -7601.4 347.3 0.00

Null 3 -9031.7 3206.0 0.00

Sagebrush height Sagebrush height 661.4 ha 4 -8864.9 0.0 1.00

61.5 ha 4 -8964.0 198.3 0.00

8.7 ha 4 -8989.4 249.0 0.00

Null 3 -9031.7 331.6 0.00

Agriculture Distance to cropland Expon. decay 4 -8314.8 0.0 1.00

Linear 4 -8687.5 745.5 0.00

Null 3 -9031.7 1431.8 0.00

Edge Variety of edge types 8.7 ha 4 -8956.8 0.0 1.00

61.5 ha 4 -8987.0 60.3 0.00

Null 3 -9031.7 147.7 0.00

661.5 ha 4 -9031.7 149.7 0.00

Landscape variation Variety of land cover types 661.5 ha 4 -8787.1 0.0 1.00

8.7 ha 4 -8923.7 273.3 0.00

61.5 ha 4 -8982.0 389.8 0.00

Null 3 -9031.7 487.2 0.00

Water sources Distance to wet meadow Expon. decay 4 -8377.5 0.0 1.00

Distance to wet meadow Linear 4 -8466.6 178.2 0.00

Distance to nearest stream Linear 4 -8788.6 822.2 0.00

Distance to perennial stream Expon. decay 4 -8805.8 856.7 0.00

Distance to nearest stream Expon. decay 4 -8811.2 867.3 0.00

Distance to water body Linear 4 -8853.1 951.1 0.00

Distance to perennial stream Linear 4 -8858.2 961.5 0.00

Distance to intermittent stream Expon. decay 4 -8944.8 1134.6 0.00

Distance to intermittent stream Linear 4 -8947.0 1139.1 0.00

Distance to spring Linear 4 -8987.1 1219.3 0.00

Distance to spring Expon. decay 4 -8994.1 1233.2 0.00

Distance to water body Expon. decay 4 -8997.0 1238.9 0.00

Null Null 3 -9031.7 1306.4 0.00

Topography Roughness index 1 ha 4 -8402.1 0.0 1.00

Elevation Linear 4 -8722.7 641.3 0.00

Topographic position index 2010 m 4 -9005.5 1206.8 0.00

Null 3 -9031.7 1257.3 0.00

Topographic position index 510 m 4 -9031.7 1259.3 0.00

Summer Land cover Bare ground 8.7 ha 4 -9440.9 0.0 0.71

61.5 ha 4 -9441.8 1.8 0.29

661.4 ha 4 -9500.9 120.0 0.00

Null 3 -9503.0 122.3 0.00

Big sagebrush 661.4 ha 4 -9472.9 0.0 1.00

61.5 ha 4 -9499.2 52.6 0.00

8.7 ha 4 -9501.4 56.9 0.00

Null 3 -9503.0 58.3 0.00

Cropland 661.4 ha 4 -8125.4 0.0 1.00

61.5 ha 4 -8444.4 637.9 0.00

Null 3 -9503.0 2753.3 0.00

8.7 ha 4 -8880.8 1510.8 0.00

Forest 8.7 ha 4 -9443.8 0.0 1.00

61.5 ha 4 -9451.3 14.9 0.00

661.4 ha 4 -9500.6 113.5 0.00

Null 3 -9503.0 116.4 0.00

Herbaceous 661.4 ha 4 -8710.9 0.0 1.00

61.5 ha 4 -9033.3 644.8 0.00

8.7 ha 4 -9053.1 684.5 0.00

Null 3 -9503.0 1582.3 0.00

Non-sagebrush shrub 661.4 ha 4 -7623.2 0.0 1.00

Null 3 -9503.0 3757.7 0.00

61.5 ha 4 -8408.9 1571.5 0.00

8.7 ha 4 -8758.0 2269.5 0.00

Other sagebrush 61.5 ha 4 -9498.8 0.0 0.53

661.4 ha 4 -9499.0 0.5 0.41

8.7 ha 4 -9501.4 5.4 0.04

null 3 -9503.0 6.6 0.02

Pinyon-juniper 661.4 ha 4 -7979.3 0.0 1.00

8.7 ha 4 -7988.2 17.8 0.00

61.5 ha 4 -7995.7 32.8 0.00

Null 3 -9503.0 3045.4 0.00

Riparian 61.5 ha 4 -9312.4 0.0 1.00

661.4 ha 4 -9336.8 48.8 0.00

8.7 ha 4 -9406.2 187.6 0.00

Null 3 -9503.0 379.3 0.00

Sagebrush height Sagebrush Height 661.4 ha 4 -8665.1 0.0 1.00

61.5 ha 4 -8921.2 512.1 0.00

8.7 ha 4 -9029.2 728.1 0.00

Null 3 -9503.0 1673.9 0.00

Agriculture Distance to cropland Expon. decay 4 -7145.6 0.0 1.00

Null 3 -9503.0 4712.8 0.00

Linear 4 -8210.5 2129.7 0.00

Edge Variety of edge types 661.4 ha 4 -8010.7 0.0 1.00

Null 3 -9503.0 2982.7 0.00

61.5 ha 4 -8962.0 1902.6 0.00

8.7 ha 4 -9041.6 2061.7 0.00

Landscape variation Variety of land cover types 661.4 ha 4 -7793.1 0.0 1.00

Null 3 -9503.0 3417.8 0.00

61.5 ha 4 -8788.3 1990.3 0.00

8.7 ha 4 -8852.1 2117.9 0.00

Water sources Distance to wet meadow Linear 4 -4909.1 0.0 1.00

Distance to perennial stream Linear 4 -4942.8 67.4 0.00

Distance to wet meadow Expon. decay 4 -5065.5 312.7 0.00

Distance to perennial stream Expon. decay 4 -5874.3 1930.3 0.00

Distance to water body Expon. decay 4 -7312.5 4806.8 0.00

Distance to water body Linear 4 -7457.1 5095.9 0.00

Distance to spring Expon. decay 4 -8213.7 6609.2 0.00

Distance to spring Linear 4 -8400.7 6983.2 0.00

Distance to nearest stream Linear 4 -9400.9 8983.7 0.00

Distance to nearest stream Expon. decay 4 -9443.9 9069.6 0.00

Distance to intermittent stream Expon. decay 4 -9501.8 9185.3 0.00

Null Null 3 -9503.0 9185.9 0.00

Distance to intermittent stream Linear 4 -9503.0 9187.8 0.00

Topography Roughness index 1 ha 4 -8924.9 0.0 1.00

Elevation Linear 4 -9286.5 723.1 0.00

Topographic position index 2010 m 4 -9315.0 780.0 0.00

Topographic position index 510 m 4 -9478.7 1107.5 0.00

Null 3 -9503.0 1154.2 0.00

North SWIP Spring Land cover Annual grass 661.4 ha 4 -7322.2 0.0 1.00

61.5 ha 4 -7346.0 47.6 0.00

8.7 ha 4 -7356.6 68.9 0.00

Null 3 -7368.2 89.9 0.00

Bare ground 661.4 ha 4 -7340.5 0.0 1.00

61.5 ha 4 -7359.1 37.1 0.00

8.7 ha 4 -7362.6 44.1 0.00

Null 3 -7368.2 53.3 0.00

Big sagebrush 661.4 ha 4 -6047.1 0.0 1.00

61.5 ha 4 -6165.6 237.0 0.00

8.7 ha 4 -6298.2 502.2 0.00

Null 3 -7368.2 2640.1 0.00

Cropland 661.4 ha 4 -7282.0 0.0 0.84

61.5 ha 4 -7283.6 3.3 0.16

8.7 ha 4 -7320.3 76.6 0.00

Null 3 -7368.2 170.3 0.00

Forest 661.4 ha 4 -7321.9 0.0 1.00

61.5 ha 4 -7359.4 75.1 0.00

Null 3 -7368.2 90.6 0.00

8.7 ha 4 -7367.4 91.1 0.00

Herbaceous 661.4 ha 4 -5760.1 0.0 1.00

61.5 ha 4 -5909.7 299.3 0.00

8.7 ha 4 -6115.2 710.3 0.00

Null 3 -7368.2 3214.2 0.00

Non-sagebrush shrub 661.4 ha 4 -7083.5 0.0 1.00

61.5 ha 4 -7168.6 170.2 0.00

8.7 ha 4 -7208.3 249.6 0.00

Null 3 -7368.2 567.3 0.00

Other sagebrush 661.4 ha 4 -6330.0 0.0 1.00

61.5 ha 4 -6374.0 87.9 0.00

8.7 ha 4 -6484.2 308.3 0.00

Null 3 -7368.2 2074.2 0.00

Pinyon-juniper 8.7 ha 4 -6851.0 0.0 1.00

61.5 ha 4 -6928.2 154.3 0.00

661.4 ha 4 -7157.8 613.5 0.00

Null 3 -7368.2 1032.3 0.00

Riparian 661.4 ha 4 -7348.0 0.0 1.00

61.5 ha 4 -7357.1 18.3 0.00

8.7 ha 4 -7366.5 37.1 0.00

Null 3 -7368.2 38.4 0.00

Wet meadow 661.4 ha 4 -7294.5 0.0 1.00

61.5 ha 4 -7339.3 89.6 0.00

8.7 ha 4 -7355.4 121.9 0.00

Null 3 -7368.2 145.4 0.00

Sagebrush height Sagebrush height 661.4 ha 4 -7235.8 0.0 1.00

8.7 ha 4 -7263.9 56.3 0.00

61.5 ha 4 -7278.4 85.3 0.00

Null 3 -7368.2 262.7 0.00

Agriculture Distance to cropland Linear 4 -7345.7 0.0 1.00

Expon. decay 4 -7356.9 22.4 0.00

Null 3 -7368.2 42.9 0.00

Edge Variety of edge types 661.4 ha 4 -7042.6 0.0 1.00

61.5 ha 4 -7089.5 93.8 0.00

8.7 ha 4 -7237.7 390.1 0.00

Null 3 -7368.2 649.0 0.00

Landscape variation Variety of land cover types 8.7 ha 4 -7247.5 0.0 1.00

61.5 ha 4 -7294.9 94.7 0.00

661.4 ha 4 -7300.8 106.5 0.00

Null 3 -7368.2 239.2 0.00

Water sources Distance to spring Expon. decay 4 -7029.3 0.0 1.00

Distance to nearest stream Linear 4 -7135.0 211.5 0.00

Distance to perennial stream Expon. decay 4 -7156.3 254.1 0.00

Distance to intermittent stream Linear 4 -7163.2 267.9 0.00

Distance to wet meadow Linear 4 -7182.6 306.6 0.00

Distance to water body Expon. decay 4 -7198.0 337.4 0.00

Distance to perennial stream Linear 4 -7250.9 443.3 0.00

Distance to spring Linear 4 -7283.5 508.5 0.00

Distance to nearest stream Expon. decay 4 -7321.2 583.9 0.00

Distance to intermittent stream Expon. decay 4 -7325.9 593.2 0.00

Distance to water body Linear 4 -7331.8 605.1 0.00

Distance to water body Expon. decay 4 -7360.5 662.5 0.00

Null Null 3 -7368.2 675.8 0.00

Topography Elevation linear 4 -6724.1 0.0 1.00

Topographic position index 2010 m 4 -7216.5 984.8 0.00

Topographic position index 510 m 4 -7329.8 1211.5 0.00

Roughness index 1 ha 4 -7356.3 1264.5 0.00

Null 3 -7368.2 1286.2 0.00

Summer Land cover Annual grass 661.4 ha* 4 -8570.6 0.0 1.00

61.5 ha 4 -8678.7 216.3 0.00

8.7 ha 4 -8723.7 306.2 0.00

Null 3 -8747.5 351.9 0.00

Bare ground 661.4 ha 4 -8210.7 0.0 1.00

8.7 ha 4 -8250.3 79.1 0.00

61.5 ha 4 -8251.1 80.6 0.00

Null 3 -8747.5 1071.6 0.00

Big sagebrush 661.4 ha 4 -5815.5 0.0 1.00

61.5 ha 4 -5993.3 355.7 0.00

8.7 ha 4 -6448.1 1265.1 0.00

Null 3 -8747.5 5862.1 0.00

Cropland 61.5 ha 4 -8634.9 0.0 1.00

8.7 ha 4 -8673.0 76.1 0.00

661.4 ha 4 -8675.4 81.1 0.00

Null 3 -8747.5 223.2 0.00

Forest 661.4 ha 4 -8319.8 0.0 1.00

61.5 ha 4 -8699.2 759.0 0.00

8.7 ha 4 -8741.6 843.8 0.00

Null 3 -8747.5 853.5 0.00

Herbaceous 661.4 ha 4 -4170.6 0.0 1.00

61.5 ha 4 -4899.8 1458.5 0.00

Null 3 -8747.5 9151.9 0.00

8.7 ha 4 -5727.5 3113.8 0.00

Non-sagebrush shrub 661.4 ha 4 -6271.5 0.0 1.00

Null 3 -8747.5 4950.1 0.00

61.5 ha 4 -7165.6 1788.3 0.00

8.7 ha 4 -7691.8 2840.6 0.00

Other sagebrush 661.4 ha* 4 -6087.3 0.0 1.00

61.5 ha 4 -6122.0 69.4 0.00

8.7 ha 4 -6641.4 1108.2 0.00

Null 3 -8747.5 5318.4 0.00

Pinyon-juniper 8.7 ha 4 -7858.9 0.0 1.00

61.5 ha 4 -7932.4 147.1 0.00

661.4 ha 4 -8445.4 1173.0 0.00

Null 3 -8747.5 1775.3 0.00

Riparian 8.7 ha 4 -8655.1 0.0 1.00

61.5 ha 4 -8677.3 44.4 0.00

Null 3 -8747.5 182.8 0.00

661.4 ha 4 -8747.5 184.8 0.00

Wet meadow 661.4 ha 4 -8694.0 0.0 1.00

61.5 ha 4 -8729.6 71.2 0.00

8.7 ha 4 -8734.1 80.1 0.00

Null 3 -8747.5 105.0 0.00

Sagebrush height Sagebrush height 661.4 ha 4 -8448.3 0.0 1.00

8.7 ha 4 -8512.0 127.3 0.00

61.5 ha 4 -8542.1 187.6 0.00

Null 3 -8747.5 596.3 0.00

Agriculture Distance to cropland Linear 4 -7782.1 0.0 1.00

Expon. decay 4 -8033.5 502.8 0.00

Null 3 -8747.5 1928.9 0.00

Edge Variety of edge types 661.4 ha 4 -6752.0 0.0 1.00

Null 3 -8747.5 3989.0 0.00

61.5 ha 4 -7809.0 2114.0 0.00

8.7 ha 4 -8155.9 2807.8 0.00

Landscape variation Variety of land cover types 8.7 ha 4 -7997.5 0.0 1.00

61.5 ha 4 -8151.2 307.5 0.00

661.4 ha 4 -8328.6 662.1 0.00

Null 3 -8747.5 1498.0 0.00

Water sources Distance to spring Expon. decay 4 -5350.3 0.0 1.00

Distance to perennial stream Expon. decay 4 -6088.3 1476.1 0.00

Distance to spring Linear 4 -6692.7 2684.8 0.00

Distance to perennial stream Linear 4 -7344.7 3989.0 0.00

Distance to water body Linear 4 -8408.8 6117.0 0.00

Distance to water body Expon. decay 4 -8542.0 6383.5 0.00

Distance to nearest stream Linear 4 -8602.8 6505.0 0.00

Distance to intermittent stream Linear 4 -8637.5 6574.5 0.00

Distance to wet meadow Expon. decay 4 -8674.9 6649.2 0.00

Distance to nearest stream Expon. decay 4 -8732.8 6765.1 0.00

Distance to wet meadow Linear 4 -8743.4 6786.3 0.00

Distance to intermittent stream Expon. decay 4 -8746.4 6792.2 0.00

Null Null 3 -8747.5 6792.5 0.00

Topography Elevation Linear 4 -5993.8 0.0 1.00

Roughness Index 1 ha 4 -8450.1 4912.6 0.00

Topographic position index 2010 m 4 -8705.5 5423.3 0.00

Topographic position index 510 m 4 -8746.3 5504.9 0.00

Null 3 -8747.5 5505.4 0.00

Winter Land cover Bare ground 61.5 ha 4 -5905.1 0.0 1.00

661.4 ha 4 -5971.6 132.8 0.00

8.7 ha 4 -5974.3 138.4 0.00

Null 3 -6986.9 2161.6 0.00

Big sagebrush 661.4 ha 4 -6889.3 0.0 1.00

61.5 ha 4 -6905.8 33.0 0.00

8.7 ha 4 -6918.2 57.8 0.00

Null 3 -6986.9 193.2 0.00

Cropland 661.4 ha 4 -6920.2 0.0 1.00

61.5 ha 4 -6928.1 15.8 0.00

8.7 ha 4 -6945.1 49.7 0.00

Null 3 -6986.9 131.4 0.00

Forest 661.4 ha 4 -6866.1 0.0 1.00

8.7 ha 4 -6916.9 101.8 0.00

61.5 ha 4 -6919.1 106.0 0.00

Null 3 -6986.9 239.7 0.00

Herbaceous 661.4 ha 4 -6848.2 0.0 1.00

61.5 ha 4 -6900.5 104.6 0.00

8.7 ha 4 -6934.1 171.8 0.00

Null 3 -6986.9 275.5 0.00

Non-sagebrush shrub 661.4 ha 4 -6755.9 0.0 1.00

61.5 ha 4 -6842.9 174.0 0.00

8.7 ha 4 -6885.1 258.3 0.00

Null 3 -6986.9 460.0 0.00

Other sagebrush 661.4 ha 4 -5888.1 0.0 1.00

61.5 ha 4 -6215.4 654.6 0.00

8.7 ha 4 -6371.6 967.1 0.00

Null 3 -6986.9 2195.7 0.00

Pinyon-juniper 61.5 ha 4 -5908.9 0.0 1.00

8.7 ha 4 -5916.8 15.7 0.00

661.4 ha 4 -6190.0 562.1 0.00

Null 3 -6986.9 2154.0 0.00

Riparian 661.4 ha 4 -6022.0 0.0 1.00

61.5 ha 4 -6577.3 1110.6 0.00

Null 3 -6986.9 1927.8 0.00

8.7 ha 4 -6854.2 1664.3 0.00

Wet meadow 661.4 ha 4 -6952.1 0.0 1.00

61.5 ha 4 -6971.9 39.7 0.00

8.7 ha 4 -6980.1 56.2 0.00

Null 3 -6986.9 67.7 0.00

Sagebrush height Sagebrush height 8.7 ha 4 -6970.3 0.0 1.00

Null 3 -6986.9 31.3 0.00

61.5 ha 4 -6986.2 31.9 0.00

661.4 ha 4 -6986.6 32.6 0.00

Agriculture Distance to cropland Linear 4 -6900.5 0.0 1.00

Expon. decay 4 -6955.4 109.8 0.00

Null 3 -6986.9 170.8 0.00

Edge Variety of edge types 661.4 ha 4 -6324.2 0.0 1.00

61.5 ha 4 -6624.1 599.7 0.00

8.7 ha 4 -6809.6 970.7 0.00

Null 3 -6986.9 1323.4 0.00

Landscape variation Variety of land cover types 61.5 ha 4 -6530.3 0.0 1.00

661.4 ha 4 -6666.8 272.9 0.00

8.7 ha 4 -6708.0 355.4 0.00

Null 3 -6986.9 911.2 0.00

Water sources Distance to wet meadow Expon. decay 4 -5337.8 0.0 0.99

Distance to wet meadow Linear 4 -5343.0 10.3 0.01

Distance to water body Expon. decay 4 -5888.0 1100.3 0.00

Distance to water body Linear 4 -6067.4 1459.2 0.00

Distance to perennial stream Expon. decay 4 -6380.1 2084.6 0.00

Distance to perennial stream Linear 4 -6480.7 2285.7 0.00

Distance to spring Expon. decay 4 -6516.9 2358.1 0.00

Distance to spring Linear 4 -6621.5 2567.3 0.00

Distance to intermittent stream Linear 4 -6757.7 2839.8 0.00

Distance to nearest stream Linear 4 -6799.2 2922.7 0.00

Distance to intermittent stream Expon. decay 4 -6843.1 3010.5 0.00

Distance to intermittent stream Expon. decay 4 -6875.0 3074.4 0.00

Null Null 3 -6986.9 3296.2 0.00

Topography Roughness index 1 ha 4 -6511.5 0.0 1.00

Elevation Linear 4 -6918.2 813.3 0.00

Topographic position index 2010 m 4 -6963.6 904.3 0.00

Null 3 -6986.9 948.8 0.00

Topographic position index 510 m 4 -6986.8 950.5 0.00

South SWIP Spring Land cover Big sagebrush 661.4 ha 4 -7624.7 0.0 1.00

61.5 ha 4 -7743.8 238.3 0.00

8.7 ha 4 -7744.9 240.5 0.00

Null 3 -7749.4 247.4 0.00

Cropland 661.4 ha 4 -7199.7 0.0 1.00

61.5 ha 4 -7346.8 294.1 0.00

8.7 ha 4 -7485.1 570.8 0.00

Null 3 -7749.4 1097.3 0.00

Forest 661.4 ha 4 -7633.0 0.0 1.00

61.5 ha 4 -7664.6 63.2 0.00

8.7 ha 4 -7684.3 102.6 0.00

Null 3 -7749.4 230.9 0.00

Herbaceous 661.4 ha 4 -7217.3 0.0 1.00

61.5 ha 4 -7501.6 568.6 0.00

8.7 ha 4 -7584.4 734.1 0.00

Null 3 -7749.4 1062.1 0.00

Non-sagebrush shrub 661.4 ha 4 -7736.2 0.0 0.99

61.5 ha 4 -7741.2 9.8 0.01

8.7 ha 4 -7745.4 18.3 0.00

Null 3 -7749.4 24.3 0.00

Other sagebrush 661.4 ha 4 -7367.9 0.0 1.00

61.5 ha 4 -7456.5 177.2 0.00

8.7 ha 4 -7490.2 244.6 0.00

Null 3 -7749.4 761.0 0.00

Pinyon-juniper 61.5 ha 4 -6351.5 0.0 1.00

8.7 ha 4 -6415.2 127.3 0.00

661.4 ha 4 -6493.3 283.7 0.00

Null 3 -7749.4 2793.8 0.00

Riparian 8.7 ha 4 -7724.1 0.0 1.00

61.5 ha 4 -7733.5 18.9 0.00

661.4 ha 4 -7739.2 30.2 0.00

Null 3 -7749.4 48.6 0.00

Sagebrush height Sagebrush height 661.4 ha 4 -7728.4 0.0 0.99

8.7 ha 4 -7733.5 10.1 0.01

61.5 ha 4 -7743.0 29.1 0.00

Null 3 -7749.4 39.9 0.00

Agriculture Distance to cropland Expon. decay 4 -5996.0 0.0 1.00

Null 3 -7749.4 3504.7 0.00

Linear 4 -6901.8 1811.6 0.00

Edge Variety of edge types 661.4 ha 4 -7735.8 0.0 0.93

61.5 ha 4 -7738.3 5.1 0.07

Null 3 -7749.4 25.2 0.00

8.7 ha 4 -7749.1 26.5 0.00

Landscape variation Variety of land cover types 661.4 ha 4 -7104.8 0.0 1.00

61.5 ha 4 -7657.9 1106.1 0.00

Null 3 -7749.4 1287.1 0.00

8.7 ha 4 -7748.5 1287.3 0.00

Water sources Distance to wet meadow Expon. decay 4 -7215.4 0.0 1.00

Distance to intermittent stream Expon. decay 4 -7242.7 54.5 0.00

Distance to nearest stream Expon. decay 4 -7270.3 109.7 0.00

Distance to wet meadow Linear 4 -7318.6 206.3 0.00

Distance to intermittent stream Linear 4 -7363.5 296.1 0.00

Distance to nearest stream Linear 4 -7377.6 324.4 0.00

Distance to water body Linear 4 -7444.1 457.3 0.00

Distance to water body Expon. decay 4 -7575.8 720.8 0.00

Distance to spring Expon. decay 4 -7621.8 812.8 0.00

Distance to perennial stream Expon. decay 4 -7642.3 853.8 0.00

Distance to spring Linear 4 -7644.6 858.4 0.00

Distance to perennial stream Linear 4 -7647.8 864.7 0.00

Null Null 3 -7749.4 1065.9 0.00

Elevation Roughness index 1 ha 4 -6804.8 0.0 1.00

Elevation Linear 4 -6888.8 167.8 0.00

Topographic position index 2010 m 4 -7725.2 1840.7 0.00

Null 3 -7749.4 1887.1 0.00

Topographic position index 510 m 4 -7749.2 1888.7 0.00

Summer Land cover Big sagebrush 661.4 ha 4 -6640.2 0.0 1.00

61.5 ha 4 -7015.3 750.2 0.00

Null 3 -7028.5 774.7 0.00

8.7 ha 4 -7028.5 776.7 0.00

Cropland 661.4 ha 4 -4581.6 0.0 1.00

61.5 ha 4 -5609.6 2056.0 0.00

8.7 ha 4 -6037.7 2912.1 0.00

Null 3 -7028.5 4891.8 0.00

Forest1 61.5 ha 4 -6703.2 0.0 1.00

661.4 ha 4 -6821.3 236.2 0.00

8.7 ha 4 -6872.1 337.9 0.00

Null 3 -7028.5 648.6 0.00

Herbaceous 661.4 ha 4 -5228.8 0.0 1.00

61.5 ha 4 -5764.5 1071.3 0.00

8.7 ha 4 -6025.7 1593.6 0.00

Null 3 -7028.5 3597.3 0.00

Non-sagebrush shrub 661.4 ha 4 -6799.9 0.0 1.00

61.5 ha 4 -6822.5 45.2 0.00

8.7 ha 4 -6923.0 246.2 0.00

Null 3 -7028.5 455.2 0.00

Other sagebrush 661.4 ha 4 -5437.5 0.0 1.00

61.5 ha 4 -5909.4 943.8 0.00

8.7 ha 4 -6078.4 1281.8 0.00

Null 3 -7028.5 3180.0 0.00

Pinyon-juniper 61.5 ha 4 -5065.3 0.0 1.00

8.7 ha 4 -5215.8 301.0 0.00

661.4 ha 4 -5343.1 555.7 0.00

Null 3 -7028.5 3924.4 0.00

Riparian 661.4 ha 4 -6915.7 0.0 1.00

61.5 ha 4 -6998.5 165.5 0.00

8.7 ha 4 -7007.5 183.5 0.00

Null 3 -7028.5 223.6 0.00

Sagebrush height Sagebrush height 661.4 ha 4 -6773.8 0.0 1.00

8.7 ha 4 -6993.1 438.5 0.00

61.5 ha 4 -7013.6 479.6 0.00

Null 3 -7028.5 507.4 0.00

Agriculture Distance to cropland Expon. decay 4 -3249.2 0.0 1.00

Linear 4 -3679.9 861.5 0.00

Null 3 -7028.5 7556.6 0.00

Edge Variety of edge types 61.5 ha 4 -6700.8 0.0 1.00

661.4 ha 4 -6757.4 113.1 0.00

8.7 ha 4 -6955.3 508.9 0.00

Null 3 -7028.5 653.4 0.00

Landscape variation Variety of land cover types 661.4 ha 4 -4940.8 0.0 1.00

61.5 ha 4 -6174.6 2467.6 0.00

8.7 ha 4 -6914.9 3948.2 0.00

Null 3 -7028.5 4173.4 0.00

Distance to wet meadow Linear 4 -4803.9 0.0 1.00

Distance to wet meadow Expon. decay 4 -5242.5 877.1 0.00

Distance to spring Linear 4 -5550.2 1492.5 0.00

Distance to nearest stream Linear 4 -6013.0 2418.1 0.00

Distance to intermittent stream Linear 4 -6013.0 2418.2 0.00

Distance to spring Expon. decay 4 -6053.0 2498.2 0.00

Distance to intermittent stream Expon. decay 4 -6215.7 2823.6 0.00

Distance to perennial stream Linear 4 -6252.1 2896.3 0.00

Distance to nearest stream Expon. decay 4 -6286.0 2964.1 0.00

Distance to water body Linear 4 -6428.2 3248.6 0.00

Distance to water body Expon. decay 4 -6787.3 3966.8 0.00

Distance to perennial stream Expon. decay 4 -6788.4 3969.0 0.00

Null Null 3 -7028.5 4447.2 0.00

Topography Elevation Linear 4 -5554.2 0.0 1.00

Roughness index 1 ha 4 -6095.2 1082.1 0.00

Topographic position index 2010 m 4 -6892.1 2675.9 0.00

Topographic position index 510 m 4 -7023.1 2937.9 0.00

Null 3 -7028.5 2946.7 0.00

Toiyabe Spring Land cover Annual grass 661.4 ha 4 -12497.2 0.0 1.00

8.7 ha 4 -12510.9 27.3 0.00

61.5 ha 4 -12515.1 35.9 0.00

Null 3 -12518.2 40.1 0.00

Big sagebrush 661.4 ha 4 -11183.1 0.0 1.00

61.5 ha 4 -11457.0 547.9 0.00

8.7 ha 4 -11658.5 950.8 0.00

Null 3 -12518.2 2668.3 0.00

Cropland 61.5 ha 4 -12349.7 0.0 0.93

8.7 ha 4 -12352.3 5.3 0.07

661.4 ha 4 -12448.3 197.3 0.00

Null 3 -12518.2 335.1 0.00

Herbaceous 661.4 ha 4 -11264.8 0.0 1.00

61.5 ha 4 -11563.1 596.6 0.00

8.7 ha 4 -11616.1 702.6 0.00

Null 3 -12518.2 2504.8 0.00

Non-sagebrush shrub 8.7 ha 4 -12474.8 0.0 1.00

661.4 ha 4 -12515.2 81.0 0.00

Null 3 -12518.2 85.0 0.00

61.5 ha 4 -12518.2 86.9 0.00

Other sagebrush 661.4 ha 4 -10428.8 0.0 1.00

61.5 ha 4 -10908.4 959.3 0.00

8.7 ha 4 -11396.2 1934.8 0.00

Null 3 -12518.2 4176.9 0.00

Pinyon-juniper 61.5 ha 4 -11028.5 0.0 1.00

8.7 ha 4 -11049.4 41.7 0.00

661.4 ha 4 -11467.4 877.7 0.00

Null 3 -12518.2 2977.4 0.00

Riparian 661.4 ha 4 -12329.3 0.0 1.00

61.5 ha 4 -12435.6 212.7 0.00

8.7 ha 4 -12454.2 249.8 0.00

Null 3 -12518.2 375.9 0.00

Sagebrush height Sagebrush height 661.4 ha 4 -11157.4 0.0 1.00

61.5 ha 4 -11509.5 704.1 0.00

8.7 ha 4 -11802.2 1289.6 0.00

Null 3 -12518.2 2719.6 0.00

Agriculture Distance to cropland Linear 4 -11584.6 0.0 1.00

Expon. decay 4 -12138.0 1106.9 0.00

Null 3 -12518.2 1865.4 0.00

Edge Variety of edge types 661.4 ha 4 -12197.4 0.0 1.00

61.5 ha 4 -12321.4 248.0 0.00

8.7 ha 4 -12432.6 470.5 0.00

Null 3 -12518.2 639.8 0.00

Landscape variation1 Variety of land cover types 661.4 ha 4 -12199.8 0.0 1.00

61.5 ha 4 -12301.4 203.3 0.00

8.7 ha 4 -12395.0 390.5 0.00

Null 3 -12518.2 634.9 0.00

Water sources Distance to spring Linear 4 -11228.5 0.0 1.00

Distance to perennial stream Linear 4 -11501.1 545.0 0.00

Distance to wet meadow Linear 4 -11860.7 1264.3 0.00

Distance to water body Linear 4 -11878.4 1299.8 0.00

Distance to spring Expon. decay 4 -12073.4 1689.7 0.00

Distance to water body Expon. decay 4 -12139.3 1821.6 0.00

Distance to wet meadow Expon. decay 4 -12237.4 2017.7 0.00

Distance to perennial stream Expon. decay 4 -12332.7 2208.3 0.00

Distance to intermittent stream Linear 4 -12349.0 2240.9 0.00

Distance to nearest stream Linear 4 -12393.3 2329.6 0.00

Distance to intermittent stream Expon. decay 4 -12432.5 2407.9 0.00

Distance to nearest stream Expon. decay 4 -12453.1 2449.2 0.00

Null Null 3 -12518.2 2577.4 0.00

Topography Elevation Linear 4 -11512.9 0.0 1.00

Roughness index 1 ha 4 -12069.3 1112.9 0.00

Topographic position index 2010 m 4 -12250.6 1475.5 0.00

Null 3 -12518.2 2008.8 0.00

Topographic position index 510 m 4 -12473.7 1921.8 0.00

Summer Land cover Annual grass 661.4 ha 4 -14753.1 0.0 0.58

8.7 ha 4 -14753.6 1.0 0.36

61.5 ha 4 -14756.0 5.7 0.03

Null 3 -14757.1 6.0 0.03

Big sagebrush 661.4 ha 4 -12461.2 0.0 1.00

61.5 ha 4 -13357.3 1792.1 0.00

8.7 ha 4 -13836.9 2751.4 0.00

Null 3 -14757.1 4589.8 0.00

Cropland 61.5 ha 4 -14486.8 0.0 1.00

8.7 ha 4 -14540.2 106.8 0.00

661.4 ha 4 -14668.8 364.0 0.00

Null 3 -14757.1 538.6 0.00

Herbaceous 661.4 ha 4 -10286.4 0.0 1.00

61.5 ha 4 -12207.3 3841.8 0.00

8.7 ha 4 -12972.3 5371.9 0.00

Null 3 -14757.1 8939.5 0.00

Non-sagebrush shrub 661.4 ha 4 -13629.7 0.0 1.00

61.5 ha 4 -13836.8 414.1 0.00

8.7 ha 4 -13940.8 622.0 0.00

Null 3 -14757.1 2252.7 0.00

Other sagebrush 661.4 ha 4 -12736.2 0.0 1.00

61.5 ha 4 -13865.3 2258.2 0.00

8.7 ha 4 -14366.0 3259.6 0.00

Null 3 -14757.1 4039.9 0.00

Pinyon-juniper 61.5 ha 4 -13010.7 0.0 1.00

8.7 ha 4 -13083.8 146.2 0.00

661.4 ha 4 -13325.0 628.6 0.00

Null 3 -14757.1 3490.8 0.00

Riparian 661.4 ha 4 -13501.7 0.0 1.00

61.5 ha 4 -13738.2 472.9 0.00

8.7 ha 4 -14080.6 1157.7 0.00

Null 3 -14757.1 2508.8 0.00

Sagebrush height Sagebrush height 661.4 ha 4 -13138.9 0.0 1.00

61.5 ha 4 -13324.7 371.7 0.00

8.7 ha 4 -13639.2 1000.6 0.00

Null 3 -14757.1 3234.5 0.00

Agriculture Distance to cropland Linear 4 -14334.2 0.0 1.00

Expon. decay 4 -14596.0 523.5 0.00

Null 3 -14757.1 843.7 0.00

Edge Variety of edge types 661.4 ha 4 -11825.6 0.0 1.00

61.5 ha 4 -13117.0 2582.8 0.00

8.7 ha 4 -13532.8 3414.4 0.00

Null 3 -14757.1 5861.0 0.00

Landscape variation Variety of land cover types 661.4 ha 4 -12437.8 0.0 1.00

61.5 ha 4 -12653.9 432.1 0.00

8.7 ha 4 -13407.2 1938.9 0.00

Null 3 -14757.1 4636.6 0.00

Water sources Distance to perennial stream Linear 4 -11089.3 0.0 1.00

Distance to spring Linear 4 -11704.3 1230.1 0.00

Distance to spring Expon. decay 4 -12320.9 2463.3 0.00

Distance to perennial stream Expon. decay 4 -12763.8 3349.1 0.00

Distance to wet meadow Linear 4 -13194.6 4210.8 0.00

Distance to wet meadow Expon. decay 4 -13481.3 4784.2 0.00

Distance to nearest stream Expon. decay 4 -14546.6 6914.8 0.00

Distance to water body Linear 4 -14551.2 6924.0 0.00

Distance to nearest stream Linear 4 -14651.8 7125.1 0.00

Distance to intermittent stream Expon. decay 4 -14667.3 7156.1 0.00

Distance to intermittent stream Linear 4 -14719.5 7260.5 0.00

Distance to water body Expon. decay 4 -14744.7 7310.9 0.00

Null Null 3 -14757.1 7333.7 0.00

Topography Elevation Linear 4 -11412.7 0.0 1.00

Roughness index 1 ha 4 -13978.4 5131.3 0.00

Topographic position index 510 m 4 -14482.7 6139.9 0.00

Topographic position index 2010 m 4 -14736.8 6648.2 0.00

Null 3 -14757.1 6686.7 0.00

Winter Land cover Annual grass 661.4 ha 4 -10450.5 0.0 0.63

Null 3 -10452.7 2.3 0.20

8.7 ha 4 -10452.5 3.9 0.09

61.5 ha 4 -10452.5 4.0 0.09

Big sagebrush 661.4 ha 4 -10202.7 0.0 1.00

61.5 ha 4 -10290.4 175.4 0.00

8.7 ha 4 -10347.0 288.6 0.00

Null 3 -10452.7 498.0 0.00

Cropland 661.4 ha 4 -10380.2 0.0 0.96

8.7 ha 4 -10383.4 6.4 0.04

61.5 ha 4 -10393.6 26.8 0.00

Null 3 -10452.7 143.0 0.00

Herbaceous 661.4 ha 4 -10391.7 0.0 1.00

61.5 ha 4 -10421.3 59.2 0.00

8.7 ha 4 -10436.9 90.2 0.00

Null 3 -10452.7 119.9 0.00

Non-sagebrush shrub 661.4 ha 4 -9818.3 0.0 1.00

61.5 ha 4 -9910.5 184.5 0.00

8.7 ha 4 -10008.7 380.8 0.00

Null 3 -10452.7 1266.8 0.00

Other sagebrush 661.4 ha 4 -8265.8 0.0 1.00

61.5 ha 4 -8642.7 753.8 0.00

8.7 ha 4 -8974.0 1416.5 0.00

Null 3 -10452.7 4371.7 0.00

Pinyon-juniper 61.5 ha 4 -9168.7 0.0 1.00

661.4 ha 4 -9211.0 84.5 0.00

8.7 ha 4 -9243.7 150.0 0.00

Null 3 -10452.7 2565.9 0.00

Riparian 661.4 ha 4 -10397.4 0.0 0.99

61.5 ha 4 -10401.7 8.6 0.01

8.7 ha 4 -10426.3 57.9 0.00

Null 3 -10452.7 108.6 0.00

Sagebrush height Sagebrush height 661.4 ha 4 -10338.7 0.0 1.00

61.5 ha 4 -10368.0 58.6 0.00

8.7 ha 4 -10378.3 79.2 0.00

Null 3 -10452.7 225.9 0.00

Agriculture Distance to cropland Linear 4 -9260.0 0.0 1.00

Expon. decay 4 -9490.7 461.5 0.00

Null 3 -10452.7 2383.4 0.00

Edge Variety of edge types 661.4 ha 4 -10445.3 0.0 0.99

8.7 ha 4 -10450.1 9.7 0.01

61.5 ha 4 -10450.8 11.1 0.00

Null 3 -10452.7 12.8 0.00

Landscape variation Variety of land cover types 661.4 ha 4 -10208.4 0.0 1.00

8.7 ha 4 -10451.5 486.2 0.00

Null 3 -10452.7 486.6 0.00

61.5 ha 4 -10452.2 487.8 0.00

Water sources Distance to spring Linear 4 -8722.8 0.0 1.00

Distance to spring Expon. decay 4 -9162.2 878.7 0.00

Distance to water body Linear 4 -9241.4 1037.1 0.00

Distance to water body Expon. decay 4 -9541.1 1636.6 0.00

Distance to wet meadow Linear 4 -10221.2 2996.7 0.00

Distance to perennial stream Linear 4 -10223.9 3002.1 0.00

Distance to nearest stream Expon. decay 4 -10308.9 3172.2 0.00

Distance to intermittent stream Expon. decay 4 -10359.3 3272.9 0.00

Distance to nearest stream Linear 4 -10391.3 3336.9 0.00

Distance to wet meadow Expon. decay 4 -10404.2 3362.8 0.00

Distance to intermittent stream Linear 4 -10424.9 3404.2 0.00

Distance to perennial stream Expon. decay 4 -10449.6 3453.5 0.00

Null Null 3 -10452.7 3457.7 0.00

Topography Elevation Linear 4 -10383.3 0.0 1.00

Topographic position index 510 m 4 -10430.4 94.2 0.00

Topographic position index 2010 m 4 -10438.5 110.4 0.00

Null 3 -10452.7 136.7 0.00

Roughness index 1 ha 4 -10452.2 137.9 0.00

Tuscarora Spring Land cover Annual grass 661.4 ha 4 -13254.6 0.0 1.00

61.5 ha 4 -13660.0 811.0 0.00

8.7 ha 4 -13736.8 964.5 0.00

Null 3 -13828.3 1145.5 0.00

Bare ground 661.4 ha 4 -12376.8 0.0 1.00

61.5 ha 4 -12980.8 1207.9 0.00

8.7 ha 4 -13166.2 1578.8 0.00

Null 3 -13828.3 2900.9 0.00

Big sagebrush 661.4 ha 4 -13057.1 0.0 1.00

61.5 ha 4 -13425.4 736.5 0.00

8.7 ha 4 -13536.6 958.9 0.00

Null 3 -13828.3 1540.3 0.00

Cropland 661.4 ha 4 -13481.3 0.0 1.00

61.5 ha 4 -13798.2 633.8 0.00

8.7 ha 4 -13823.7 684.8 0.00

Null 3 -13828.3 692.0 0.00

Forest 61.5 ha 4 -13571.0 0.0 1.00

8.7 ha 4 -13595.2 48.5 0.00

661.4 ha 4 -13664.4 186.9 0.00

Null 3 -13828.3 512.7 0.00

Herbaceous 8.7 ha 4 -13191.1 0.0 0.89

61.5 ha 4 -13193.2 4.1 0.11

661.4 ha 4 -13350.0 317.7 0.00

Null 3 -13828.3 1272.3 0.00

Non-sagebrush shrub 8.7 ha 4 -13763.5 0.0 1.00

61.5 ha 4 -13790.2 53.4 0.00

661.4 ha 4 -13817.3 107.7 0.00

Null 3 -13828.3 127.7 0.00

Other sagebrush 61.5 ha 4 -13609.8 0.0 1.00

8.7 ha 4 -13695.1 170.6 0.00

661.4 ha 4 -13715.0 210.5 0.00

Null 3 -13828.3 435.0 0.00

Riparian 8.7 ha 4 -13784.5 0.0 1.00

61.5 ha 4 -13792.3 15.5 0.00

661.4 ha 4 -13824.4 79.8 0.00

Null 3 -13828.3 85.6 0.00

Sagebrush height Sagebrush height 661.4 ha 4 -12628.2 0.0 1.00

61.5 ha 4 -13324.2 1391.9 0.00

8.7 ha 4 -13553.1 1849.7 0.00

Null 3 -13828.3 2398.1 0.00

Agriculture Distance to cropland Expon. decay 4 -13399.9 0.0 1.00

Linear 4 -13613.5 427.3 0.00

Null 3 -13828.3 854.9 0.00

Edge Variety of edge types 661.4 ha 4 -13744.4 0.0 1.00

61.5 ha 4 -13761.5 34.3 0.00

8.7 ha 4 -13780.1 71.3 0.00

Null 3 -13828.3 165.8 0.00

Landscape variation Variety of land cover types 8.7 ha 4 -13772.6 0.0 1.00

661.4 ha 4 -13781.6 18.0 0.00

61.5 ha 4 -13822.5 99.8 0.00

Null 3 -13828.3 109.3 0.00

Water sources Distance to wet meadow Linear 4 -11554.1 0.0 1.00

Distance to nearest stream Linear 4 -12659.6 2210.9 0.00

Distance to wet meadow Expon. decay 4 -12710.1 2311.9 0.00

Distance to nearest stream Expon. decay 4 -12838.8 2569.4 0.00

Distance to intermittent stream Expon. decay 4 -13081.2 3054.2 0.00

Distance to intermittent stream Linear 4 -13197.6 3287.0 0.00

Distance to water body Expon. decay 4 -13396.3 3684.3 0.00

Distance to water body Linear 4 -13719.6 4330.9 0.00

Distance to spring Linear 4 -13722.1 4335.9 0.00

Distance to perennial stream Linear 4 -13788.6 4469.0 0.00

Distance to perennial stream Expon. decay 4 -13794.5 4480.8 0.00

Distance to spring Expon. decay 4 -13822.2 4536.1 0.00

Null Null 3 -13828.3 4546.3 0.00

Topography Roughness index 1 ha 4 -13371.4 0.0 1.00

Topographic position index 510 m 4 -13697.7 652.5 0.00

Topographic position index 2010 m 4 -13771.2 799.6 0.00

Elevation Linear 4 -13795.2 847.5 0.00

Null 3 -13828.3 911.7 0.00

Summer Land cover Annual grass 661.4 ha 4 -15709.2 0.0 1.00

61.5 ha 4 -16451.5 1484.7 0.00

8.7 ha 4 -16776.7 2135.0 0.00

Null 3 -17349.5 3278.6 0.00

Bare ground 661.4 ha 4 -12964.8 0.0 1.00

61.5 ha 4 -13642.1 1354.6 0.00

8.7 ha 4 -13924.6 1919.6 0.00

Null 3 -17349.5 8767.4 0.00

Big sagebrush 661.4 ha 4 -16881.9 0.0 1.00

61.5 ha 4 -17101.0 438.1 0.00

8.7 ha 4 -17140.8 517.7 0.00

Null 3 -17349.5 933.1 0.00

Cropland 661.4 ha 4 -15110.2 0.0 1.00

61.5 ha 4 -15746.1 1271.8 0.00

8.7 ha 4 -15908.8 1597.3 0.00

Null 3 -17349.5 4476.6 0.00

Forest 61.5 ha 4 -17218.5 0.0 1.00

8.7 ha 4 -17273.0 109.1 0.00

661.4 ha 4 -17287.9 139.0 0.00

Null 3 -17349.5 260.0 0.00

Herbaceous 661.4 ha 4 -16444.4 0.0 1.00

61.5 ha 4 -16692.3 495.8 0.00

8.7 ha 4 -16740.4 592.0 0.00

Null 3 -17349.5 1808.2 0.00

Non-sagebrush shrub 661.4 ha 4 -16972.6 0.0 1.00

61.5 ha 4 -17221.6 497.9 0.00

8.7 ha 4 -17234.0 522.7 0.00

Null 3 -17349.5 751.7 0.00

Other sagebrush 661.4 ha 4 -16947.9 0.0 1.00

61.5 ha 4 -17010.9 126.0 0.00

8.7 ha 4 -17020.1 144.4 0.00

Null 3 -17349.5 801.1 0.00

Riparian 661.4 ha 4 -16163.1 0.0 1.00

61.5 ha 4 -17164.8 2003.3 0.00

8.7 ha 4 -17247.6 2168.9 0.00

Null 3 -17349.5 2370.7 0.00

Sagebrush height Sagebrush height 661.4 ha 4 -16422.6 0.0 1.00

61.5 ha 4 -16765.7 686.3 0.00

8.7 ha 4 -17018.3 1191.5 0.00

Null 3 -17349.5 1851.8 0.00

Agriculture Distance to cropland Expon. decay 4 -16611.5 0.0 1.00

Linear 4 -17237.0 1251.0 0.00

Null 3 -17349.5 1473.9 0.00

Edge Variety of edge types 661.4 ha 4 -16457.3 0.0 1.00

61.5 ha 4 -17068.2 1221.8 0.00

8.7 ha 4 -17225.7 1536.7 0.00

Null 3 -17349.5 1782.4 0.00

Landscape variation Variety of land cover types 61.5 ha 4 -16951.0 0.0 1.00

8.7 ha 4 -17051.7 201.4 0.00

661.4 ha 4 -17341.0 780.0 0.00

Null 3 -17349.5 794.9 0.00

Water sources Distance to wet meadow Linear 4 -15145.3 0.0 1.00

Distance to wet meadow Expon. decay 4 -16680.1 3069.5 0.00

Distance to perennial stream Linear 4 -17048.8 3807.0 0.00

Distance to water body Expon. decay 4 -17203.7 4116.9 0.00

Distance to intermittent stream Linear 4 -17279.5 4268.5 0.00

Distance to perennial stream Expon. decay 4 -17283.4 4276.3 0.00

Distance to spring Linear 4 -17291.6 4292.7 0.00

Distance to nearest stream Linear 4 -17296.5 4302.5 0.00

Distance to intermittent stream Expon. decay 4 -17323.6 4356.6 0.00

Distance to spring Expon. decay 4 -17331.8 4372.9 0.00

Null Null 3 -17349.5 4406.4 0.00

Distance to nearest stream Expon. decay 4 -17349.2 4407.8 0.00

Distance to water body Linear 4 -17349.5 4408.4 0.00

Topography Roughness index 1 ha 4 -16884.8 0.0 1.00

Topographic position index 510 m 4 -17337.4 905.2 0.00

Topographic position index 2010 m 4 -17344.5 919.5 0.00

Null 3 -17349.5 927.4 0.00

Elevation Linear 4 -17348.9 928.1 0.00

Winter Land cover Annual grass 661.4 ha 4 -7363.6 0.0 1.00

61.5 ha 4 -7906.4 1085.7 0.00

8.7 ha 4 -8128.4 1529.5 0.00

Null 3 -8310.8 1892.5 0.00

Bare ground 661.4 ha 4 -8255.0 0.0 1.00

8.7 ha 4 -8297.8 85.6 0.00

61.5 ha 4 -8307.0 104.0 0.00

Null 3 -8310.8 109.7 0.00

Big sagebrush 661.4 ha 4 -8247.5 0.0 1.00

8.7 ha 4 -8277.2 59.4 0.00

61.5 ha 4 -8279.2 63.5 0.00

Null 3 -8310.8 124.7 0.00

Cropland 8.7 ha 4 -8244.7 0.0 1.00

61.5 ha 4 -8282.0 74.7 0.00

661.4 ha 4 -8285.9 82.5 0.00

Null 3 -8310.8 130.3 0.00

Forest 661.4 ha 4 -8128.1 0.0 1.00

61.5 ha 4 -8138.1 19.9 0.00

8.7 ha 4 -8151.5 46.8 0.00

Null 3 -8310.8 363.5 0.00

Herbaceous 661.4 ha 4 -8249.4 0.0 1.00

61.5 ha 4 -8302.8 106.9 0.00

8.7 ha 4 -8308.3 117.8 0.00

Null 3 -8310.8 121.0 0.00

Non-sagebrush shrub 61.5 ha 4 -7742.8 0.0 1.00

8.7 ha 4 -7755.6 25.6 0.00

661.4 ha 4 -7903.2 320.9 0.00

Null 3 -8310.8 1134.2 0.00

Other sagebrush 661.4 ha 4 -7372.7 0.0 1.00

61.5 ha 4 -7471.1 196.7 0.00

8.7 ha 4 -7716.4 687.4 0.00

Null 3 -8310.8 1874.3 0.00

Riparian 61.5 ha 4 -8199.5 0.0 1.00

8.7 ha 4 -8263.3 127.6 0.00

661.4 ha 4 -8309.5 220.0 0.00

Null 3 -8310.8 220.7 0.00

Sagebrush height Sagebrush height 661.4 ha 4 -8032.8 0.0 1.00

61.5 ha 4 -8191.3 317.0 0.00

8.7 ha 4 -8208.2 350.7 0.00

Null 3 -8310.8 554.0 0.00

Agriculture Distance to cropland Linear 4 -7943.6 0.0 1.00

Expon. decay 4 -8037.3 187.5 0.00

Null 3 -8310.8 732.6 0.00

Edge Variety of edge cover types 61.5 ha 4 -8002.5 0.0 1.00

8.7 ha 4 -8196.4 387.7 0.00

661.4 ha 4 -8255.2 505.4 0.00

Null 3 -8310.8 614.7 0.00

Landscape variation Variety of land cover types 61.5 ha 4 -7900.4 0.0 1.00

8.7 ha 4 -8092.6 384.4 0.00

661.4 ha 4 -8180.7 560.5 0.00

Null 3 -8310.8 818.8 0.00

Water sources Distance to wet meadow Linear 4 -7232.9 0.0 1.00

Distance to nearest stream Linear 4 -7667.8 869.7 0.00

Distance to nearest stream Expon. decay 4 -7793.8 1121.8 0.00

Distance to intermittent stream Expon. decay 4 -7929.1 1392.4 0.00

Distance to wet meadow Expon. decay 4 -8043.5 1621.1 0.00

Distance to spring Linear 4 -8043.9 1622.0 0.00

Distance to intermittent stream Linear 4 -8046.0 1626.2 0.00

Distance to water body Expon. decay 4 -8121.7 1777.5 0.00

Distance to spring Expon. decay 4 -8270.0 2074.2 0.00

Distance to perennial stream Expon. decay 4 -8289.7 2113.5 0.00

Distance to perennial stream Linear 4 -8303.8 2141.8 0.00

Distance to water body Linear 4 -8307.1 2148.4 0.00

Null Null 3 -8310.8 2153.8 0.00

Topography Roughness index 1 ha 4 -7308.0 0.0 1.00

Elevation Linear 4 -8138.6 1661.2 0.00

Topographic position index 2010 m 4 -8282.5 1949.0 0.00

Topographic position index 510 m 4 -8300.8 1985.7 0.00

Null 3 -8310.8 2003.7 0.00

Virginia Spring Land cover Annual grass 661.4 ha 4 -2076.7 0.0 1.00

61.5 ha 4 -2578.4 1003.4 0.00

8.7 ha 4 -2949.9 1746.6 0.00

Null 3 -4720.3 5285.3 0.00

Bare ground 661.4 ha 4 -4670.8 0.0 0.60

8.7 ha 4 -4671.2 0.9 0.38

61.5 ha 4 -4674.2 6.8 0.02

Null 3 -4720.3 97.1 0.00

Big sagebrush 661.4 ha 4 -3870.9 0.0 1.00

61.5 ha 4 -4000.7 259.7 0.00

8.7 ha 4 -4036.4 331.1 0.00

Null 3 -4720.3 1696.9 0.00

Cropland 661.4 ha 4 -4495.2 0.0 1.00

61.5 ha 4 -4567.9 145.5 0.00

8.7 ha 4 -4643.2 296.0 0.00

Null 3 -4720.3 448.3 0.00

Forest 661.4 ha 4 -4712.7 0.0 0.97

8.7 ha 4 -4716.2 6.9 0.03

Null 3 -4720.3 13.3 0.00

61.5 ha 4 -4719.6 13.9 0.00

Herbaceous 661.4 ha 4 -2190.0 0.0 1.00

61.5 ha 4 -2656.6 933.2 0.00

8.7 ha 4 -2912.8 1445.5 0.00

Null 3 -4720.3 5058.6 0.00

Non-sagebrush shrub 8.7 ha 4 -4483.7 0.0 1.00

61.5 ha 4 -4490.1 12.7 0.00

661.4 ha 4 -4510.2 52.9 0.00

Null 3 -4720.3 471.2 0.00

Other sagebrush 661.4 ha 4 -4686.5 0.0 1.00

61.5 ha 4 -4713.9 54.9 0.00

8.7 ha 4 -4717.5 62.1 0.00

Null 3 -4720.3 65.7 0.00

Riparian 61.5 ha 4 -4714.8 0.0 0.62

8.7 ha 4 -4715.4 1.1 0.35

661.4 ha 4 -4718.5 7.4 0.02

Null 3 -4720.3 9.0 0.01

Sagebrush Height Sagebrush Height 661.4 ha 4 -4702.7 0.0 1.00

61.4 ha 4 -4715.5 25.7 0.00

Null 3 -4720.3 33.3 0.00

8.7 ha 4 -4720.2 35.1 0.00

Agriculture Distance to cropland Expon. decay 4 -4556.0 0.0 1.00

Linear 4 -4675.0 237.9 0.00

Null 3 -4720.3 326.6 0.00

Edge Variety of edge types 661.4 ha 4 -3016.8 0.0 1.00

61.5 ha 4 -3744.8 1456.0 0.00

8.7 ha 4 -4181.0 2328.3 0.00

Null 3 -4720.3 3405.0 0.00

Landscape variation Variety of land cover types 8.7 ha 4 -4308.1 0.0 1.00

61.5 ha 4 -4467.9 319.5 0.00

661.4 ha 4 -4628.7 641.1 0.00

Null 3 -4720.3 822.4 0.00

Water sources Distance to spring Linear 4 -3760.9 0.0 1.00

Distance to spring Expon. decay 4 -4171.8 822.0 0.00

Distance to wet meadow Linear 4 -4475.1 1428.5 0.00

Distance to nearest stream Expon. decay 4 -4564.8 1607.8 0.00

Distance to intermittent stream Linear 4 -4633.0 1744.4 0.00

Distance to intermittent stream Expon. decay 4 -4655.3 1788.8 0.00

Distance to wet meadow Expon. decay 4 -4665.4 1809.0 0.00

Distance to perennial stream Linear 4 -4705.7 1889.6 0.00

Distance to water body Linear 4 -4711.7 1901.6 0.00

Distance to water body Expon. decay 4 -4717.0 1912.3 0.00

Null null 3 -4720.3 1917.0 0.00

Distance to nearest stream Linear 4 -4720.3 1918.8 0.00

Distance to perennial stream Expon. decay 4 -4720.3 1918.9 0.00

Topography Elevation Linear 4 -3373.5 0.0 1.00

Topographic position index 2010 m 4 -4469.7 2192.5 0.00

Roughness index 1 ha 4 -4627.6 2508.3 0.00

Topographic position index 510 m 4 -4642.0 2537.1 0.00

Null 3 -4720.3 2691.8 0.00

Summer Land cover Annual grass 661.4 ha 4 -628.1 0.0 1.00

61.5 ha 4 -829.9 403.5 0.00

8.7 ha 4 -975.3 694.3 0.00

Null 3 -1622.0 1985.7 0.00

Bare ground 661.4 ha 4 -932.0 0.0 1.00

8.7 ha 4 -1100.7 337.4 0.00

61.5 ha 4 -1177.9 491.9 0.00

Null 3 -1622.0 1378.0 0.00

Big sagebrush 661.4 ha 4 -932.0 0.0 1.00

61.5 ha 4 -1100.7 337.4 0.00

8.7 ha 4 -1177.9 491.9 0.00

Null 3 -1622.0 1378.0 0.00

Cropland 661.4 ha 4 -1431.2 0.0 1.00

61.5 ha 4 -1573.8 285.2 0.00

8.7 ha 4 -1597.4 332.4 0.00

Null 3 -1622.0 379.5 0.00

Forest 661.4 ha 4 -1602.6 0.0 1.00

61.5 ha 4 -1620.4 35.6 0.00

Null 3 -1622.0 36.7 0.00

8.7 ha 4 -1621.9 38.5 0.00

Herbaceous 661.4 ha 4 -463.3 0.0 1.00

61.5 ha 4 -697.6 468.6 0.00

8.7 ha 4 -819.8 713.0 0.00

Null 3 -1622.0 2315.3 0.00

Non-sagebrush shrub 661.4 ha 4 -1049.9 0.0 1.00

61.5 ha 4 -1174.3 248.7 0.00

8.7 ha 4 -1239.8 379.7 0.00

Null 3 -1622.0 1142.1 0.00

Other sagebrush 8.7 ha 4 -1581.9 0.0 1.00

61.5 ha 4 -1600.1 36.4 0.00

661.4 ha 4 -1614.7 65.6 0.00

Null 3 -1622.0 78.2 0.00

Riparian 661.4 ha 4 -1571.7 0.0 1.00

61.5 ha 4 -1605.1 66.7 0.00

Null 3 -1622.0 98.5 0.00

8.7 ha 4 -1621.1 98.8 0.00

Sagebrush height Sagebrush height 8.7 ha 4 -1620.2 0.0 0.48

Null 3 -1622.0 1.4 0.24

661.4 ha 4 -1621.3 2.0 0.18

61.5 ha 4 -1621.8 3.1 0.10

Distance to cropland Expon. decay 4 -1283.0 0.0 1.00

Linear 4 -1382.7 199.4 0.00

Null 3 -1622.0 675.9 0.00

Edge Variety of edge cover types 661.4 ha 4 -824.3 0.0 1.00

61.5 ha 4 -1083.4 518.2 0.00

8.7 ha 4 -1324.9 1001.1 0.00

Null 3 -1622.0 1593.2 0.00

Landscape variation Variety of land cover types 8.7 ha 4 -1382.5 0.0 1.00

61.5 ha 4 -1442.8 120.7 0.00

661.4 ha 4 -1555.1 345.2 0.00

Null 3 -1622.0 476.9 0.00

Water sources Distance to spring Linear 4 -1193.0 0.0 1.00

Distance to spring Expon. decay 4 -1343.4 300.9 0.00

Distance to wet meadow Linear 4 -1505.6 625.3 0.00

Distance to perennial Linear 4 -1554.6 723.4 0.00

Distance to nearest stream Expon. decay 4 -1569.2 752.5 0.00

Distance to intermittent stream Linear 4 -1587.9 790.0 0.00

Distance to water body Linear 4 -1589.8 793.7 0.00

Distance to wet meadow Expon. decay 4 -1592.9 799.8 0.00

Distance to intermittent stream Expon. decay 4 -1599.7 813.4 0.00

Distance to perennial stream Expon. decay 4 -1618.6 851.2 0.00

Null Null 3 -1622.0 856.0 0.00

Distance to nearest stream Linear 4 -1621.3 856.6 0.00

Distance to water body Expon. decay 4 -1621.9 857.9 0.00

Topography Elevation Linear 4 -690.0 0.0 1.00

Topographic position index 2010 m 4 -1606.3 1832.5 0.00

Topographic position index 510 m 4 -1609.1 1838.2 0.00

Roughness index 1 ha 4 -1615.1 1850.1 0.00

Null 3 -1622.0 1861.8 0.00

*Model failed to converge. Was not carried forward in the RSF modeling procedure.

**Table S3.** Model averaged parameter estimates and 95-percent confidence intervals for candidate variables included seasonal resource selection function (RSF) models from 10 sites within Nevada and northeastern California.

Site Season Covariate Scale Estimate (95% CI) Selection/Avoidance

Buffalo-Skedaddle Spring Bare ground 61.5 ha 0.38 (0.09, 0.66) Selection

Big sagebrush 661.4 ha -13.26 (-14.78, -11.74) Avoidance

Forest 661.4 ha -25.97 (-33.16, -18.77) Avoidance

Herbaceous 661.4 ha 8.57 (7.77, 9.38) Selection

Non-sagebrush shrub 61.5 ha -6.53 (-7.78, -5.29) Avoidance

Riparian 661.4 ha -57.72 (-67.65, -47.79) Avoidance

Wet meadow 661.4 ha -16.05 (-20.31, -11.79) Avoidance

Distance to cropland Expon. decay -0.70 (-0.86, -0.53) Avoidance

Variety of land cover types 61.5 ha -0.48 (-0.51, -0.46) Avoidance

Distance to perennial stream Expon. decay -1.11 (-1.27, -0.94) Avoidance

Distance to spring Expon. decay -0.77 (-0.95, -0.59) Avoidance

Distance to water body Linear -0.24 (-0.27, -0.21) Selection

Elevation Linear -0.86 (-1.10, -0.62) Selection for lower elevation

Topographic position index 510 m 0.009 (0.005, 0.01) Selected ridges/Avoided valleys

Summer Annual grass 661.4 ha 0.26 (-0.91, 1.44) None

Bare ground 661.4 ha -4.25 (-4.59, -3.92) Avoidance

Big sagebrush 661.4 ha -10.97 (-12.69, -9.25) Avoidance

Cropland 661.4 ha 0.48 (-0.26, 1.22) None

Forest 661.4 ha -4.46 (-6.25, -2.67) Avoidance

Herbaceous 61.5 ha 9.98 (9.36, 10.60) Selection

Other sagebrush 661.4 ha -8.12 (-9.86, -6.38) Avoidance

Riparian 661.4 ha -16.84 (-23.68, -10.01) Avoidance

Wet meadow 661.4 ha -14.96 (-17.70, -12.21) Avoidance

Variety of land cover types 61.5 ha -0.19 (-0.22, -0.17) Avoidance

Distance to perennial stream Expon. decay -1.46 (-1.64, -1.29) Avoidance

Distance to spring Expon. decay -2.70 (-2.93, -2.47) Avoidance

Distance to water body Linear -0.29 (-0.33, -0.25) Selection

Elevation Linear -0.25 (-0.53, 0.02) None

Roughness index 1 ha -17.91 (-18.59, -17.24) Avoidance

Topographic position index 2010 m 0.005 (0.004, 0.007) Selected ridges/Avoided valleys

Winter Annual grass 661.4 ha 11.37 (9.04, 13.69) Selection

Bare ground 8.7 ha 0.02 (0.01, 0.03) Selection

Big sagebrush 661.4 ha -0.21 (-0.25, -0.18) Avoidance

Cropland 661.4 ha 7.98 (6.56, 9.40) Selection

Herbaceous 8.7 ha 0.01 (0.002, 0.02) Selection

Non-sagebrush shrub 661.4 ha -0.25 (-0.28, -0.21) Avoidance

Other sagebrush 661.4 ha -0.12 (-0.16, -0.09) Avoidance

Variety of land cover types 61.5 ha -0.34 (-0.39, -0.29) Avoidance

Distance to perennial stream Expon. decay -1.69 (-2.00, -1.38) Avoidance

Distance to spring Expon. decay -2.64 (-2.98, -2.30) Avoidance

Elevation Linear 0.62 (0.14, 1.10) Selection for higher elevation

Roughness index 1 ha -8.49 (-9.72, -7.26) Avoidance

Topographic position index 510 m 0.008 (0.001, 0.015) Selected ridges/Avoided valleys

Cortez Spring Annual Grass 8.7 ha -0.91 (-1.07, -0.74) Avoidance

Bare ground 8.7 ha 2.38 (2.23, 2.54) Selection

Big sagebrush 661.4 ha 1.11 (0.34, 1.87) Selection

Herbaceous 8.7 ha 0.03 (-0.39, 0.45) None

Non-sagebrush shrub 661.4 ha 13.52 (12.55, 14.49) Selection

Other sagebrush 661.4 ha 59.01 (57.03, 61.00) Selection

Pinyon-juniper 8.7 ha -6.14 (-6.63, -5.66) Avoidance

Riparian 661.4 ha -39.33 (-42.37, -36.30) Avoidance

Sagebrush height 661.4 ha -2.96 (-3.21, -2.71) Avoidance

Distance to cropland Expon. decay -0.83 (-0.94, -0.71) Avoidance

Variety of land cover types 61.5 ha -0.20 (-0.22, -0.19) Avoidance

Distance to intermittent stream Linear -0.40 (-0.47, -0.33) Selection

Distance to spring Linear -0.03 (-0.04, -0.02) Selection

Distance to water body Expon. decay 0.43 (0.30, 0.56) Selection

Distance to wet meadow Expon. decay 0.72 (0.56, 0.87) Selection

Elevation Linear 0.59 (0.46, 0.71) Selection for higher elevations

Roughness index 1 ha -1.13 (-1.41, -0.84) Avoidance

Topographic position index 510 m 0.01 (0.01, 0.01) Selected ridges/Avoided valleys

Summer Annual grass 661.4 ha 5.72 (5.15, 6.28) Selection

Bare ground 661.4 ha -0.67 (-1.12, -0.23) Avoidance

Big sagebrush 661.4 ha 19.98 (18.02, 21.94) Selection

Forest 61.5 ha -57.12 (-102.06, -12.18) Avoidance

Herbaceous 661.4 ha 19.36 (17.89, 20.84) Selection

Non-sagebrush shrub 61.5 ha 21.36 (19.56, 23.15) Selection

Other sagebrush 661.4 ha 78.93 (72.96, 84.90) Selection

Pinyon-juniper 61.5 ha -10.38 (-11.41, -9.35) Avoidance

Riparian 661.4 ha -78.32 (-86.95, -69.70) Avoidance

Sagebrush height 8.7 ha 0.48 (0.07, 0.89) Selection

Distance to cropland Linear -0.21 (-0.34, -0.09) Avoidance

Variety of land cover types 661.4 ha 0.41 (0.38, 0.45) Selection

Distance to perennial stream Expon. decay 2.46 (2.21, 2.71) Selection

Distance to spring Expon. decay 4.86 (4.56, 5.16) Selection

Distance to water body Linear -0.21 (-0.24, -0.18) Selection

Distance to wet meadow Expon. decay -2.16 (-2.57, -1.74) Avoidance

Elevation Linear 5.76 (5.39, 6.14) Selection for higher elevation

Roughness index 1 ha 1.38 (0.62, 2.15) Selection

Topographic position index 510 m -0.005 (-0.008, -0.001) Avoided ridges/Selected valleys

Winter Annual grass 661.4 ha 0.82 (0.22, 1.43) Selection

Bare ground 8.7 ha 3.14 (2.61, 3.66) Selection

Big sagebrush 661.4 ha 3.24 (1.10, 5.37) Selection

Cropland 61.5 ha -6.43 (-10.46, -2.39) Avoidance

Herbaceous 661.4 ha 2.97 (1.41, 4.53) Selection

Non-sagebrush shrub 661.4 ha 14.72 (12.05, 17.39) Selection

Other sagebrush 61.5 ha 36.70 (31.72, 41.68) Selection

Pinyon-juniper 61.5 ha -0.79 (-1.67, 0.10) None

Riparian 661.4 ha -22.94 (-31.20, -14.68) Avoidance

Variety of land cover types 8.7 ha -0.19 (-0.25, -0.12) Avoidance

Distance to perennial stream Linear -0.10 (-0.12, -0.08) Selection

Distance to spring Expon. decay 0.76 (0.48, 1.04) Selection

Distance to water body Linear -0.13 (-0.16, -0.10) Selection

Distance to wet meadow Expon. decay 0.21 (-0.23, 0.66) Selection

Elevation Linear 1.24 (0.20, 2.28) Selection for higher elevations

Roughness index 1 ha 1.14 (0.34, 1.95) Selection

Topographic position index 510 m 0.004 (-0.001, 0.009) Selected ridges/Avoided valleys

Gollaher Spring Forest 661.4 ha -133.95 (-149.11, -118.78) Avoidance

Herbaceous 661.4 ha 3.13 (2.16, 4.10) Selection

Non-sagebrush shrub 661.4 ha -41.26 (-43.86, -38.65) Avoidance

Other sagebrush 661.4 ha 158.88 (152.99, 164.76) Selection

Riparian 61.5 ha -19.95 (-22.05, -17.85) Avoidance

Sagebrush height 661.4 ha -15.06 (-15.90, -14.22) Avoidance

Distance to cropland Linear -0.51 (-0.55, -0.47) Selection

Variety of edge types 661.4 ha -0.33 (-0.35, -0.31) Avoidance

Variety of land cover types 61.5 ha -0.47 (-0.51, -0.44) Avoidance

Distance to intermittent stream Linear 1.87 (1.71, 2.03) Avoidance

Distance to spring Expon. decay -0.16 (-0.40, 0.08) None

Distance to water body Expon. decay 0.26 (0.00, 0.51) Selection

Roughness index 1 ha -3.65 (-4.21, -3.09) Avoidance

Topographic Position Index 2010 m 0.01 (0.01, 0.01) Selected ridges/Avoided valleys

Summer Forest 661.4 ha -6.90 (-8.28, -5.52) Avoidance

Herbaceous 661.4 ha 7.37 (6.42, 8.33) Selection

Non-sagebrush shrub 661.4 ha -12.97 (-14.83, -11.10) Avoidance

Other sagebrush 661.4 ha 39.57 (33.29, 45.85) Selection

Riparian 661.4 ha -11.14 (-12.85, -9.44) Avoidance

Sagebrush height 61.5 ha -2.14 (-2.67, -1.62) Avoidance

Distance to cropland Linear -0.34 (-0.37, -0.30) Selection

Variety of edge types 661.4 ha -0.08 (-0.10, -0.06) Avoidance

Variety of land cover types 661.4 ha 0.06 (0.03, 0.09) Selection

Distance to intermittent spring Expon. decay -0.91 (-1.10, -0.73) Avoidance

Distance to spring Linear -0.53 (-0.58, -0.48) Selection

Distance to water body Linear -0.39 (-0.44, -0.34) Selection

Roughness index 1 ha -5.52 (-6.21, -4.83) Avoidance

Topographic position index 2010 m 0.005 (0.004, 0.006) Selected ridges/Avoided valleys

Winter Cropland 661.4 ha -3.25 (-12.81, 6.32) Avoidance

Forest 661.4 ha -10.80 (-18.90, -2.71) Avoidance

Herbaceous 61.5 ha -3.17 (-5.59, -0.76) Avoidance

Non-sagebrush shrub 661.4 ha -16.85 (-22.40, -11.30) Avoidance

Other sagebrush 661.4 ha 144.07 (130.08, 158.06) Selection

Riparian 61.5 ha -5.64 (-9.18, -2.11) Avoidance

Sagebrush height 661.4 ha -3.18 (-4.84, -1.52) Avoidance

Variety of land cover types 8.7 ha -0.21 (-0.33, -0.09) Avoidance

Distance to intermittent stream Expon. decay -2.67 (-3.24, -2.10) Avoidance

Distance to spring Expon. decay 0.20 (-0.46, 0.86) None

Distance to water body Linear -0.47 (-0.59, -0.36) Selection

Roughness index 1 ha -2.78 (-4.34, -1.21) Avoidance

Topographic position index 2010 m 0.008 (0.005, 0.01) Selected ridges/Avoided valleys

Lincoln Spring Big sagebrush 661.4 ha 15.43 (12.79, 18.07) Selection

Forest 8.7 ha -17.62 (-23.74, -11.51) Avoidance

Herbaceous 661.4 ha 18.74 (15.57, 21.92) Selection

Non-sagebrush shrub 61.5 ha -14.68 (-17.41, -11.96) Avoidance

Pinyon-juniper 61.5 ha -13.41 (-15.80, -11.03) Avoidance

Riparian 8.7 ha 2.11 (0.33, 3.90) Selection

Distance to cropland Linear -0.59 (-0.66, -0.52) Selection

Variety of land cover types 661.4 ha 0.35 (0.31, 0.39) Selection

Distance to perennial stream Linear -0.31 (-0.35, -0.28) Selection

Distance to spring Linear -0.44 (-0.49, -0.39) Selection

Distance to water body Linear -0.53 (-0.59, -0.47) Selection

Distance to wet meadow Expon. decay 3.35 (2.95, 3.74) Selection

Roughness index 1 ha -9.05 (-10.23, -7.86) Avoidance

Topographic position index 2010 m -0.002 (-0.005, 0.0002) Avoided ridges/Selected valleys

Summer Big sagebrush 661.4 ha 21.02 (18.08, 23.95) Selection

Forest 8.7 ha -1.23 (-1.82, -0.65) Avoidance

Herbaceous 661.4 ha 42.85 (38.67, 47.03) Selection

Non-sagebrush shrub 661.4 ha 19.29 (16.42, 22.15) Selection

Pinyon-juniper 61.5 ha -15.07 (-16.62, -13.51) Avoidance

Riparian 661.4 ha 9.30 (4.72, 13.88) Selection

Sagebrush height 61.5 ha 2.12 (1.08, 3.16) Selection

Distance to cropland Linear -1.01 (-1.12, -0.91) Selection

Variety of edge types 61.5 ha 0.24 (0.20, 0.28) Selection

Variety of land cover types 661.4 ha 0.64 (0.58, 0.70) Selection

Distance to perennial stream Linear -0.35 (-0.39, -0.30) Selection

Distance to spring Linear -0.65 (-0.73, -0.57) Selection

Distance to water body Linear -0.87 (-0.96, -0.79) Selection

Distance to wet meadow Expon. decay 0.27 (-0.10, 0.65) None

Roughness index 1 ha 0.03 (-1.37, 1.44) None

Topographic position index 510 m 0.0009 (-0.004, 0.01) None

Winter Big sagebrush 661.4 ha 11.58 (9.05, 14.11) Selection

Forest 661.4 ha 1.43 (0.26, 2.60) Selection

Non-sagebrush shrub 61.5 ha -7.60 (-10.53, -4.68) Avoidance

Other sagebrush 661.4 ha 90.51 (75.34, 105.68) Selection

Pinyon-juniper 661.4 ha -15.21 (-17.08, -13.34) Avoidance

Riparian 61.5 ha -11.20 (-17.27, -5.13) Avoidance

Sagebrush height 661.4 ha 2.67 (1.33, 4.01) Selection

Distance to cropland Linear -0.24 (-0.29, -0.19) Selection

Variety of land cover types 8.7 ha -0.39 (-0.50, -0.27) Avoidance

Distance to nearest stream Linear -2.15 (-2.57, -1.73) Selection

Distance to spring Expon. decay 1.56 (1.17, 1.96) Selection

Distance to water body Linear -0.16 (-0.20, -0.11) Selection

Distance to wet meadow Linear -0.04 (-0.05, -0.03) Selection

Topographic position index 510 m 0.02 (0.01, 0.03) Selected ridges/Avoided valleys

Midway Spring Cropland 61.5 ha 9.51 (8.60, 10.42) Selection

Forest 61.5 ha -2.10 (-5.33, 1.14) None

Herbaceous 8.7 ha 15.76 (14.38, 17.13) Selection

Non-sagebrush shrub 8.7 ha 3.91 (3.10, 4.72) Selection

Other sagebrush 61.5 ha 48.26 (44.24, 52.29) Selection

Pinyon-juniper 61.5 ha -29.56 (-31.80, -27.32) Avoidance

Sagebrush height 661.4 ha 4.30 (3.69, 4.90) Selection

Variety of edge types 8.7 ha 0.24 (0.21, 0.28) Selection

Variety of land cover types 661.4 ha 0.27 (0.24, 0.29) Selection

Distance to nearest stream Linear -1.82 (-2.01, -1.62) Selection

Distance to spring Linear -0.16 (-0.18, -0.15) Selection

Distance to water body Linear 0.16 (0.15, 0.17) Avoidance

Distance to wet meadow Expon. decay 2.68 (2.48, 2.89) Selection

Roughness index 1 ha -1.09 (-1.72, -0.45) Avoidance

Topographic position index 2010 m -0.002 (-0.004, -0.001) Avoided ridges/Selected valleys

Summer Forest 8.7 ha -10.00 (-11.89, -8.11) Avoidance

Herbaceous 661.4 ha 36.25 (33.90, 38.60) Selection

Non-sagebrush shrub 661.4 ha 44.87 (41.44, 48.31) Selection

Other sagebrush 61.5 ha 57.77 (52.74, 62.80) Selection

Pinyon-juniper 661.4 ha -18.52 (-19.99, -17.06) Avoidance

Riparian 61.5 ha -0.10 (-2.22, 2.01) None

Sagebrush height 661.4 ha -2.56 (-3.17, -1.95) Avoidance

Distance to cropland Expon. decay 1.36 (1.12, 1.59) Selection

Variety of edge types 661.4 ha 0.22 (0.20, 0.24) Selection

Variety of land cover types 661.4 ha -0.22 (-0.19, -0.25) Avoidance

Distance to spring Expon. decay 2.75 (2.52, 2.99) Selection

Distance to water body Expon. decay 3.68 (3.41, 3.95) Selection

Distance to wet meadow Linear -0.58 (-0.60, -0.56) Selection

Topographic position index 2010 m -0.015 (-0.017, -0.013) Avoided ridges/Selected valleys

North SWIP Spring Annual grass 661.4 ha -93.41 (-153.57, -33.25) Avoidance

Big sagebrush 661.4 ha 13.83 (12.74, 14.91) Selection

Cropland 661.4 ha 18.25 (16.80, 19.69) Selection

Forest 661.4 ha -10.08 (-12.25, -7.91) Avoidance

Herbaceous 661.4 ha 31.48 (25.96, 37.00) Selection

Non-sagebrush shrub 661.4 ha -11.47 (-13.10, -9.85) Avoidance

Other sagebrush 661.4 ha 63.96 (58.99, 68.93) Selection

Pinyon-juniper 8.7 ha -3.42 (-3.90, -2.94) Avoidance

Riparian 661.4 ha -12.75 (-16.10, -9.40) Avoidance

Variety of edge types 661.4 ha -0.004 (-0.02, 0.01) None

Variety of land cover types 8.7 ha -0.11 (-0.15, -0.07) Avoidance

Distance to nearest stream Linear -1.44 (-1.63, -1.25) Selection

Distance to spring Expon. decay -0.21 (-0.38, -0.03) Avoidance

Distance to water body Linear 0.01 (-0.001, 0.03) Avoidance

Distance to wet meadow Linear 0.10 (0.09, 0.103) Avoidance

Elevation Linear 0.25 (0.04, 0.46) Selection for higher elevations

Topographic position index 2010 m 0.001 (-0.0003, 0.002) None

Summer Annual grass 8.7 ha -39.76 (-66.51, -13.01) Avoidance

Big sagebrush 661.4 ha 12.57 (11.26, 13.88) Selection

Forest 661.4 ha -5.43 (-7.54, -3.32) Avoidance

Herbaceous 661.4 ha 59.36 (56.58, 62.14) Selection

Non-sagebrush shrub 661.4 ha 11.53 (9.75, 13.31) Selection

Other sagebrush 61.5 ha 14.43 (10.37, 18.49) Selection

Pinyon-juniper 8.7 ha -4.53 (-5.29, -3.77) Avoidance

Riparian 8.7 ha 6.32 (5.22, 7.42) Selection

Sagebrush height 661.4 ha 3.29 (2.49, 4.08) Selection

Distance to cropland Linear 0.09 (0.07, 0.10) Avoidance

Variety of edge types 661.4 ha 0.13 (0.11, 0.14) Selection

Variety of land cover types 8.7 ha 0.28 (0.24, 0.32) Selection

Distance to spring Expon. decay 3.39 (3.15, 3.62) Selection

Distance to water body Linear -0.06 (-0.08, -0.04) Selection

Distance to wet meadow Expon. decay -0.97 (-1.23, -0.72) Avoidance

Elevation Linear 1.74 (1.48, 2.00) Selection for higher elevation

Topographic position index 2010 m -0.007 (-0.008, -0.006) Avoided ridges/Selected valleys

Winter Bare ground 61.5 ha 6.65 (6.23, 7.06) Selection

Big sagebrush 661.4 ha 4.50 (3.43, 5.56) Selection

Forest 661.4 ha -8.19 (-11.09, -5.29) Avoidance

Herbaceous 661.4 ha 27.88 (25.30, 30.46) Selection

Non-sagebrush shrub 661.4 ha -8.37 (-10.82, -5.92) Avoidance

Other sagebrush 661.4 ha 90.93 (85.25, 96.60) Selection

Riparian 661.4 ha -158.90 (-171.11, -146.69) Avoidance

Sagebrush height 8.7 ha -0.30 (-0.72, 0.12) None

Distance to cropland Linear -0.30 (-0.32, -0.27) Selection

Variety of edge types 661.4 ha -0.17 (-0.18, -0.15) Avoidance

Distance to water body Expon. decay -4.84 (-5.12, -4.56) Avoidance

Distance to wet meadow Expon. decay -7.63 (-7.99, -7.28) Avoidance

Topographic position index 2010 m -0.004 (-0.005, -0.002) Avoided ridges /Selected valleys

South SWIP Spring Big sagebrush 661.4 ha 8.13 (6.94, 9.31) Selection

Forest 661.4 ha 1.03 (-1.22, 3.29) None

Herbaceous 661.4 ha -38.62 (-40.89, -36.36) Avoidance

Non-sagebrush shrubs 661.4 ha -7.51 (-10.20, -4.81) Avoidance

Pinyon-juniper 61.5 ha -9.60 (-10.27, -8.93) Avoidance

Riparian 8.7 ha -6.14 (-7.39, -4.88) Avoidance

Distance to cropland Expon. decay 3.89 (3.70, 4.08) Selection

Variety of edge types 661.4 ha -0.18 (-0.20, -0.16) Avoidance

Variety of land cover types 661.4 ha 0.58 (0.55, 0.61) Selection

Distance to intermittent stream Expon. decay 1.19 (1.04, 1.33) Selection

Distance to spring Expon. decay -0.33 (-0.53, -0.13) Avoidance

Distance to water body Linear -0.11 (-0.14, -0.08) Selection

Roughness index 1 ha -9.95 (-10.71, -9.20) Avoidance

Topographic position index 2010 m 0.0002 (-0.001, 0.002) Selected ridges/Avoided valleys

Summer Big sagebrush 661.4 ha 19.83 (18.08, 21.57) Selection

Forest 661.4 ha -1.19 (-4.57, 2.18) None

Herbaceous 661.4 ha -26.51 (-29.99, -23.03) Avoidance

Non-sagebrush shrub 661.4 ha 0.27 (-3.44, 3.98) None

Pinyon-juniper 61.5 ha -12.28 (-13.66, -10.90) Avoidance

Riparian 661.4 ha -27.41 (-33.97, -20.86) Avoidance

Distance to cropland Expon. decay 7.50 (7.16, 7.84) Selection

Variety of edge types 61.5 ha -0.42 (-0.46, -0.37) Avoidance

Distance to nearest stream Linear -3.25 (-3.67, -2.83) Selection

Distance to spring Linear -0.19 (-0.22, -0.16) Selection

Distance to water body Linear -0.31 (-0.36, -0.26) Selection

Distance to wet meadow Linear -0.11 (-0.13, -0.09) Selection

Elevation Linear -1.55 (-2.00, -1.10) Selection for lower elevations

Topographic position index 2010 m -0.009 (-0.012, -0.007) Avoided ridges/Selected valleys

Toiyabe Spring Annual grass 661.4 ha 5.95 (4.59, 7.32) Selection

Herbaceous 661.4 ha 20.54 (19.50, 21.58) Selection

Non-sagebrush shrub 8.7 ha 14.39 (13.36, 15.43) Selection

Other sagebrush 661.4 ha 101.10 (97.66, 104.54) Selection

Pinyon-juniper 61.5 ha -55.50 (-59.87, -51.14) Avoidance

Riparian 661.4 ha 27.20 (25.07, 29.32) Selection

Sagebrush height 661.4 ha 10.67 (10.26, 11.08) Selection

Distance to cropland Linear -0.26 (-0.28, -0.25) Selection

Variety of edge types 661.4 ha 0.14 (0.13, 0.15) Selection

Distance to spring Linear -0.55 (-0.58, -0.53) Selection

Distance to water body Linear -0.15 (-0.16, -0.14) Selection

Distance to wet meadow Linear -0.18 (-0.19, -0.18) Selection

Summer Annual grass 661.4 ha -8.50 (-10.02, -6.98) Avoidance

Big sagebrush 661.4 ha 28.94 (27.56, 30.33) Selection

Herbaceous 661.4 ha 32.38 (31.16, 33.61) Selection

Non-sagebrush shrub 661.4 ha -5.64 (-7.60, -3.69) Avoidance

Other sagebrush 661.4 ha 57.89 (54.52, 61.27) Selection

Pinyon-juniper 61.5 ha -51.83 (-55.73, -47.94) Avoidance

Riparian 661.4 ha 26.06 (24.44, 27.69) Selection

Distance to cropland Linear -0.34 (-0.36, -0.32) Selection

Variety of edge types 661.4 ha 0.41 (0.40, 0.42) Selection

Distance to perennial stream Linear -0.35 (-0.37, -0.33) Selection

Distance to water body Linear -0.10 (-0.12, -0.09) Selection

Distance to wet meadow Linear -0.115 (-0.12, -0.11) Selection

Roughness index 1 ha -4.41 (-4.93, -3.88) Avoidance

Topographic position index 510 m -0.015 (-0.016, -0.013) Avoided ridges/Selected valleys

Winter Annual grass 661.4 ha -2.08 (-3.86, -0.31) Avoidance

Big sagebrush 661.4 ha -8.05 (-9.59, -6.51) Avoidance

Herbaceous 661.4 ha -6.31 (-7.65, -4.98) Avoidance

Non-sagebrush shrub 661.4 ha -13.36 (-15.53, -11.19) Avoidance

Other sagebrush 661.4 ha 86.04 (82.12, 89.97) Selection

Pinyon-juniper 61.5 ha -56.43 (-61.83, -51.02) Avoidance

Riparian 661.4 ha -1.41 (-3.26, 0.44) None

Distance to cropland Linear -0.34 (-0.36, -0.32) Selection

Variety of edge types 661.4 ha -0.25 (-0.26, -0.24) Avoidance

Distance to spring Linear -0.71 (-0.75, -0.68) Selection

Distance to water body Linear -0.31 (-0.33, -0.30) Selection

Distance to wet meadow Linear -0.12 (-0.13, -0.11) Selection

Topographic position index 510 m 0.008 (0.006, 0.01) Selected ridges/Avoided valleys

Tuscarora Spring Annual grass 661.4 ha -21.80 (-25.15, -18.45) Avoidance

Bare ground 661.4 ha -6.68 (-7.00, -6.36) Avoidance

Forest 61.5 ha -13.32 (-14.92, -11.71) Avoidance

Herbaceous 8.7 ha 7.77 (7.37, 8.17) Selection

Non-sagebrush shrub 8.7 ha -25.41 (-26.44, -24.38) Avoidance

Other sagebrush 61.5 ha 71.81 (68.70, 74.93) Selection

Riparian 8.7 ha -3.18 (-3.68, -2.69) Avoidance

Distance to cropland Expon. decay 0.57 (0.45, 0.69) Selection

Variety of edge types 661.4 ha 0.01 (0.00, 0.02) Selection

Variety of land cover types 8.7 ha -0.17 (-0.20, -0.15) Avoidance

Distance to nearest stream Linear 3.55 (3.36, 3.73) Avoidance

Distance to spring Linear 0.08 (0.05, 0.10) Avoidance

Distance to water body Expon. decay -2.90 (-3.08, -2.73) Avoidance

Distance to wet meadow Linear -0.19 (-0.20, -0.18) Selection

Elevation Linear -6.67 (-6.95, -6.38) Selection for lower elevations

Roughness index 1 ha -12.60 (-13.12, -12.09) Avoidance

Topographic position index 510 m 0.01 (0.01, 0.02) Selected ridges/Avoided valleys

Summer Annual grass 661.4 ha -83.20 (-90.05, -76.36) Avoidance

Bare ground 661.4 ha -14.93 (-15.31, -14.54) Avoidance

Cropland 661.4 ha 6.40 (6.05, 6.74) Selection

Forest 61.5 ha -22.23 (-23.61, -20.85) Avoidance

Herbaceous 661.4 ha -4.21 (-4.80, -3.62) Avoidance

Other sagebrush 661.4 ha 119.19 (113.65, 124.72) Selection

Riparian 661.4 ha -3.34 (-4.35, -2.34) Avoidance

Variety of edge types 661.4 ha -0.01 (-0.02, 0.01) None

Variety of land cover types 61.5 ha -0.04 (-0.06, -0.01) Avoidance

Distance to perennial stream Linear -0.02 (-0.04, 0.01) None

Distance to spring Linear 0.08 (0.05, 0.10) Avoidance

Distance to water body Expon. decay -0.87 (-1.03, -0.71) Avoidance

Distance to wet meadow Linear -0.184 (-0.19, -0.175) Selection

Roughness index 1 ha -13.39 (-13.88, -12.91) Avoidance

Topographic position index 510 m 0.0003 (-0.001, 0.002) None

Winter Annual grass 661.4 ha -106.86 (-115.55, -98.17) Avoidance

Forest 661.4 ha -17.70 (-19.96, -15.44) Avoidance

Herbaceous 661.4 ha 15.62 (14.69, 16.55) Selection

Non-sagebrush shrub 61.5 ha -35.58 (-37.16, -34.00) Avoidance

Other sagebrush 661.4 ha 68.86 (63.08, 74.63) Selection

Riparian 61.5 ha -10.51 (-11.71, -9.31) Avoidance

Sagebrush height 661.4 ha 13.20 (12.54, 13.86) Selection

Distance to cropland Linear -0.26 (-0.28, -0.23) Selection

Variety of land cover types 61.5 ha -0.46 (-0.49, -0.43) Avoidance

Distance to nearest stream Linear 4.12 (3.89, 4.35) Avoidance

Distance to spring Linear -0.51 (-0.55, -0.47) Selection

Distance to water body Expon. decay -3.25 (-3.49, -3.01) Avoidance

Distance to wet meadow Linear -0.15 (-0.16, -0.14) Selection

Elevation Linear -3.90 (-4.19, -3.61) Selection of lower elevations

Roughness index 1 ha -18.73 (-19.50, -17.95) Avoidance

Topographic position index 2010 m -0.003 (-0.004, -0.002) Avoided ridges/Selected valleys

Virginia Spring Annual grass 661.4 ha 10.88 (10.06, 11.70) Selection

Bare ground 661.4 ha -2.70 (-3.34, -2.07) Avoidance

Cropland 661.4 ha -25.17 (-36.85, -13.48) Avoidance

Forest 661.4 ha 55.25 (46.19, 64.32) Selection

Herbaceous 661.4 ha 28.32 (25.53, 31.11) Selection

Non-sagebrush shrub 8.7 ha 22.21 (19.90, 24.51) Selection

Other sagebrush 661.4 ha 23.07 (18.48, 27.67) Selection

Riparian 61.5 ha 4.14 (-1.13, 9.40) None

Sagebrush height 661.4 ha 6.58 (5.29, 7.87) Selection

Variety of land cover types 8.7 ha 0.32 (0.24, 0.40) Selection

Distance to nearest stream Expon. decay -2.49 (-2.81, -2.14) Avoidance

Distance to water body Linear 0.13 (0.07, 0.19) Avoidance

Distance to wet meadow Linear -0.35 (-0.40, -0.30) Selection

Roughness index 1 ha 3.83 (2.90, 4.76) Selection

Topographic position index 2010 m 0.011 (0.01, 0.012) Selected ridges/Avoided valleys

Summer Annual grass 661.4 ha 9.45 (9.448, 9.46) Selection

Bare ground 661.4 ha 8.69 (3.82, 13.56) Selection

Forest 661.4 ha 43.36 (27.64, 59.08) Selection

Herbaceous 661.4 ha 56.38 (56.37, 56.38) Selection

Non-sagebrush shrubs 661.4 ha 37.30 (28.51, 46.10) Selection

Other sagebrush 8.7 ha -22.79 (-32.93, -12.65) Avoidance

Riparian 661.4 ha -26.33 (-59.51, 6.85) None

Distance to cropland Expon. decay -6.18 (-6.19, -6.17) Avoidance

Variety of land cover types 8.7 ha 0.19 (-0.01, 0.40) None

Distance to perennial stream Linear -0.23 (-0.32, -0.13) Selection

Distance to water body Linear -0.66 (-0.81, 0.50) None

Distance to wet meadow Linear -0.12 (-0.23, -0.01) Selection

Roughness index 1 ha -8.77 (-11.66, -5.88) Avoidance

Topographic position index 510 m 0.04 (0.03, 0.05) Selected ridges/Avoided valleys

**Table S4.** Means and standard errors for habitat features available to, and used by, greater sage-grouse (*Centrocercus urophasianus*) in and found important in seasonal resource selection function (RSF) modeling in 10 sites within Nevada and northeastern California.

Available Used

Site Season Variable Scale Mean SE Mean SE

Buffalo-Skedaddle Spring Bare ground 61.5 ha 0.510 0.0019 0.527 0.0034

Big sagebrush 661.4 ha 0.049 0.0003 0.042 0.0007

Forest 661.4 ha 0.014 0.0009 0.000 0.0001

Herbaceous 661.4 ha 0.169 0.0007 0.187 0.0014

Non-sagebrush shrub 61.5 ha 0.043 0.0004 0.034 0.0008

Riparian 661.4 ha 0.003 0.0001 0.001 0.0001

Wet meadow 661.4 ha 0.005 0.0003 0.001 0.0002

Distance to cropland Km 2.93 0.03 3.61 0.06

Variety of land cover types 61.5 ha 3.80 0.02 2.56 0.04

Distance to perennial stream Km 4.58 0.05 5.45 0.08

Distance to spring Km 2.78 0.02 3.37 0.06

Distance to water body Km 2.07 0.02 1.82 0.03

Elevation Km 1.62 0.00 1.57 0.00

Topographic position index 510 m 0.07 0.17 1.11 0.22

Summer Annual grass 661.4 ha 0.015 0.0004 0.021 0.0009

Bare ground 661.4 ha 0.508 0.0015 0.418 0.0039

Big sagebrush 661.4 ha 0.050 0.0003 0.041 0.0006

Cropland 661.4 ha 0.010 0.0005 0.024 0.0019

Forest 661.4 ha 0.014 0.0009 0.001 0.0003

Herbaceous 61.5 ha 0.171 0.0007 0.201 0.0020

Other sagebrush 661.4 ha 0.041 0.0003 0.040 0.0006

Riparian 661.4 ha 0.003 0.0001 0.001 0.0001

Wet meadow 661.4 ha 0.005 0.0003 0.003 0.0003

Variety of land cover types 61.5 ha 3.85 0.02 2.88 0.05

Distance to perennial stream Km 4.55 0.04 6.08 0.08

Distance to spring Km 2.76 0.02 4.48 0.05

Distance to water body Km 2.05 0.02 1.47 0.02

Elevation Km 1.62 0.00 1.55 0.00

Roughness index 1 ha 0.14 0.00 0.06 0.00

Topographic position index 2010 m -0.37 0.44 -2.43 0.33

Winter Annual grass 661.4 ha 0.016 0.0009 0.021 0.0021

Bare ground 8.7 ha 0.511 0.0037 0.526 0.0085

Big sagebrush 661.4 ha 0.049 0.0006 0.040 0.0011

Cropland 661.4 ha 0.011 0.0011 0.032 0.0053

Herbaceous 8.7 ha 0.172 0.0017 0.181 0.0043

Non-sagebrush shrub 661.4 ha 0.042 0.0007 0.029 0.0013

Other sagebrush 661.4 ha 0.041 0.0006 0.037 0.0012

Variety of land cover types 61.5 ha 3.77 0.05 2.62 0.08

Distance to perennial stream Km 4.65 0.09 5.77 0.15

Distance to spring Km 2.82 0.05 3.84 0.12

Elevation Km 1.61 0.00 1.60 0.01

Roughness index 1 ha 0.14 0.00 0.08 0.00

Topographic position index 510 m -0.20 0.33 0.75 0.41

Cortez Spring Annual grass 8.7 ha 0.037 0.0012 0.028 0.0023

Bare ground 8.7 ha 0.520 0.0018 0.592 0.0024

Big sagebrush 661.4 ha 0.063 0.0003 0.076 0.0007

Herbaceous 8.7 ha 0.078 0.0005 0.088 0.0010

Non-sagebrush shrubs 661.4 ha 0.047 0.0002 0.050 0.0006

Other sagebrush 661.4 ha 0.030 0.0001 0.042 0.0003

Pinyon-juniper 8.7 ha 0.050 0.0009 0.009 0.0007

Riparian 661.4 ha 0.007 0.0001 0.003 0.0001

Sagebrush height 661.4 ha 0.32 0.00 0.31 0.00

Distance to cropland Km 3.07 0.02 3.51 0.03

Variety of land cover types 61.5 ha 3.28 0.01 2.87 0.03

Distance to intermittent stream Km 0.43 0.00 0.31 0.01

Distance to spring Km 3.14 0.02 2.83 0.04

Distance to water body Km 4.78 0.02 5.22 0.04

Distance to wet meadow Km 9.68 0.03 9.78 0.07

Elevation Km 1.92 0.00 1.95 0.00

Roughness index 1 ha 0.14 0.00 0.15 0.00

Topographic position index 510 m 0.02 0.13 3.33 0.25

Summer Annual grass 661.4 ha 0.031 0.0017 0.089 0.0073

Bare ground 661.4 ha 0.523 0.0033 0.454 0.0043

Big sagebrush 661.4 ha 0.063 0.0006 0.103 0.0016

Forest 61.5 ha 0.000 0.0000 0.000 0.0000

Herbaceous 661.4 ha 0.079 0.0009 0.140 0.0018

Non-sagebrush shrub 61.5 ha 0.047 0.0006 0.075 0.0023

Other sagebrush 661.4 ha 0.031 0.0003 0.044 0.0003

Pinyon-juniper 61.5 ha 0.048 0.0019 0.018 0.0022

Riparian 661.4 ha 0.007 0.0002 0.005 0.0003

Sagebrush height 8.7 ha 0.30 0.00 0.34 0.01

Distance to cropland Km 3.06 0.04 2.60 0.07

Variety of land cover types 661.4 ha 5.69 0.04 7.21 0.06

Distance to perennial stream Km 7.04 0.08 3.75 0.14

Distance to spring Km 3.16 0.04 1.18 0.06

Distance to water body Km 4.81 0.05 4.00 0.09

Distance to wet meadow Km 9.70 0.08 10.52 0.14

Elevation Km 1.92 0.00 2.17 0.01

Roughness index 1 ha 0.14 0.00 0.20 0.00

Topographic position index 510 m 0.12 0.29 -0.89 0.71

Winter Annual grass 661.4 ha 0.033 0.0028 0.060 0.0092

Bare ground 8.7 ha 0.525 0.0059 0.567 0.0094

Big sagebrush 661.4 ha 0.062 0.0009 0.074 0.0023

Cropland 61.5 ha 0.023 0.0034 0.001 0.0006

Herbaceous 661.4 ha 0.079 0.0013 0.092 0.0028

Non-sagebrush shrub 661.4 ha 0.048 0.0007 0.058 0.0022

Other sagebrush 61.5 ha 0.031 0.0005 0.039 0.0010

Pinyon-juniper 61.5 ha 0.046 0.0028 0.028 0.0043

Riparian 661.4 ha 0.007 0.0003 0.004 0.0004

Variety of land cover types 8.7 ha 2.19 0.03 1.96 0.06

Distance to perennial stream Km 7.03 0.12 5.26 0.23

Distance to spring Km 3.10 0.06 2.61 0.12

Distance to water body Km 4.85 0.07 4.50 0.15

Distance to wet meadow Km 9.67 0.12 9.85 0.23

Elevation Km 1.92 0.01 1.98 0.01

Roughness index 1 ha 0.14 0.00 0.16 0.00

Topographic position index 510 m -0.40 0.44 0.84 0.74

Gollaher Spring Forest 661.4 ha 0.034 0.0014 0.000 0.0001

Herbaceous 661.4 ha 0.175 0.0008 0.143 0.0016

Non-sagebrush shrub 661.4 ha 0.050 0.0005 0.019 0.0005

Other sagebrush 661.4 ha 0.035 0.0001 0.047 0.0004

Riparian 61.5 ha 0.030 0.0009 0.004 0.0005

Sagebrush height 661.4 ha 0.32 0.00 0.29 0.00

Distance to cropland Km 2.74 0.03 1.58 0.02

Variety of edge types 661.4 ha 6.19 0.04 3.86 0.06

Variety of land cover types 61.5 ha 3.53 0.02 2.28 0.04

Distance to intermittent stream Km 0.25 0.00 0.42 0.01

Distance to spring Km 2.39 0.02 3.23 0.05

Distance to water body Km 2.02 0.02 2.24 0.03

Roughness index 1 ha 0.18 0.00 0.15 0.00

Topographic position index 2010 m -0.52 0.58 5.23 0.73

Summer Forest 661.4 ha 0.033 0.0017 0.011 0.0015

Herbaceous 661.4 ha 0.175 0.0011 0.194 0.0018

Non-sagebrush shrub 661.4 ha 0.052 0.0006 0.047 0.0009

Other sagebrush 661.4 ha 0.034 0.0002 0.037 0.0003

Riparian 661.4 ha 0.028 0.0007 0.017 0.0009

Sagebrush height 61.5 ha 0.32 0.00 0.31 0.00

Distance to cropland Km 2.80 0.04 1.80 0.06

Variety of edge types 661.4 ha 6.11 0.05 5.91 0.08

Variety of land cover types 661.4 ha 6.37 0.03 6.72 0.06

Distance to intermittent stream Km 0.25 0.00 0.30 0.01

Distance to spring Km 2.44 0.03 1.65 0.03

Distance to water body Km 2.04 0.02 1.59 0.04

Roughness index 1 ha 0.18 0.00 0.16 0.00

Topographic position index 2010 m 1.44 0.77 11.38 1.43

Winter Cropland 661.4 ha 0.009 0.0013 0.003 0.0003

Forest 661.4 ha 0.030 0.0029 0.002 0.0005

Herbaceous 61.5 ha 0.174 0.0022 0.125 0.0028

Non-sagebrush shrub 661.4 ha 0.051 0.0011 0.018 0.0015

Other sagebrush 661.4 ha 0.035 0.0003 0.053 0.0007

Riparian 61.5 ha 0.030 0.0024 0.008 0.0017

Sagebrush height 661.4 ha 0.32 0.00 0.31 0.00

Variety of land cover types 8.7 ha 2.25 0.04 1.63 0.07

Distance to intermittent stream Km 0.25 0.01 0.60 0.03

Distance to spring Km 2.47 0.05 2.99 0.10

Distance to water body Km 2.05 0.04 1.61 0.06

Roughness index 1 ha 0.18 0.00 0.14 0.01

Topographic position index 2010 m 1.98 1.41 7.27 2.17

Lincoln Spring Big sagebrush 661.4 ha 0.047 0.0010 0.064 0.0026

Forest 8.7 ha 0.083 0.0065 0.001 0.0007

Herbaceous 661.4 ha 0.054 0.0008 0.068 0.0019

Non-sagebrush shrub 61.5 ha 0.056 0.0011 0.050 0.0019

Pinyon-juniper 61.5 ha 0.139 0.0043 0.016 0.0029

Riparian 8.7 ha 0.021 0.0017 0.009 0.0027

Distance to cropland Km 3.34 0.06 1.42 0.08

Variety of land cover types 661.4 ha 5.32 0.05 6.83 0.16

Distance to perennial stream Km 4.87 0.10 2.98 0.15

Distance to spring Km 3.36 0.08 2.27 0.10

Distance to water body Km 4.14 0.06 1.99 0.09

Distance to wet meadow Km 12.12 0.21 6.52 0.39

Roughness index 1 ha 0.18 0.00 0.11 0.00

Topographic position index 2010 m -1.94 1.55 -7.14 1.35

Summer Big sagebrush 661.4 ha 0.048 0.0008 0.061 0.0022

Forest 8.7 ha 0.094 0.0062 0.020 0.0050

Herbaceous 661.4 ha 0.053 0.0007 0.080 0.0021

Non-sagebrush shrub 661.4 ha 0.057 0.0008 0.082 0.0022

Pinyon-juniper 61.5 ha 0.145 0.0039 0.017 0.0023

Riparian 661.4 ha 0.016 0.0006 0.019 0.0012

Sagebrush height 61.5 ha 0.20 0.00 0.18 0.00

Distance to cropland Km 3.26 0.05 0.80 0.05

Variety of edge types 61.5 ha 2.21 0.05 4.41 0.17

Variety of land cover types 661.4 ha 5.45 0.05 8.24 0.09

Distance to perennial stream Km 4.78 0.09 2.42 0.09

Distance to spring Km 3.32 0.06 1.34 0.05

Distance to water body Km 3.95 0.06 1.06 0.05

Distance to wet meadow Km 11.65 0.18 7.45 0.35

Roughness index 1 ha 0.18 0.00 0.13 0.00

Topographic position index 510 m 0.05 0.50 2.40 0.76

Winter Big sagebrush 661.4 ha 0.048 0.0010 0.067 0.0022

Forest 661.4 ha 0.072 0.0048 0.011 0.0041

Non-sagebrush shrub 61.5 ha 0.056 0.0011 0.060 0.0017

Other sagebrush 661.4 ha 0.014 0.0002 0.021 0.0004

Pinyon-juniper 661.4 ha 0.144 0.0040 0.019 0.0025

Riparian 61.5 ha 0.018 0.0010 0.002 0.0006

Sagebrush height 661.4 ha 0.20 0.00 0.17 0.00

Distance to cropland Km 3.36 0.06 2.31 0.09

Variety of land cover types 8.7 ha 2.04 0.03 1.61 0.05

Distance to nearest stream Km 0.27 0.01 0.17 0.01

Distance to spring Km 3.39 0.08 3.63 0.16

Distance to water body Km 4.05 0.07 2.97 0.11

Distance to wet meadow Km 11.97 0.21 10.24 0.41

Topographic position index 510 m -1.49 0.57 0.81 0.45

Midway Spring Cropland 61.5 ha 0.003 0.0005 0.074 0.0044

Forest 61.5 ha 0.009 0.0007 0.001 0.0001

Herbaceous 8.7 ha 0.065 0.0005 0.077 0.0012

Non-sagebrush shrub 8.7 ha 0.060 0.0004 0.071 0.0021

Other sagebrush 61.5 ha 0.019 0.0001 0.022 0.0004

Pinyon-juniper 61.5 ha 0.082 0.0017 0.004 0.0004

Sagebrush height 661.4 ha 0.19 0.00 0.16 0.00

Variety of edge types 8.7 ha 1.09 0.01 1.37 0.04

Variety of land cover types 661.4 ha 5.02 0.02 5.82 0.06

Distance to nearest stream Km 0.25 0.00 0.17 0.00

Distance to spring Km 5.13 0.04 4.67 0.07

Distance to water body Km 4.33 0.04 5.48 0.12

Distance to wet meadow Km 12.18 0.07 8.87 0.16

Roughness index 1 ha 0.15 0.00 0.10 0.00

Topographic position index 2010 ha -0.70 0.52 -4.86 0.56

Summer Forest 8.7 ha 0.011 0.0010 0.001 0.0003

Herbaceous 661.4 ha 0.066 0.0003 0.084 0.0008

Non-sagebrush shrub 661.4 ha 0.059 0.0002 0.082 0.0007

Other sagebrush 61.5 ha 0.019 0.0001 0.019 0.0004

Pinyon-juniper 661.4 ha 0.082 0.0015 0.010 0.0007

Riparian 61.5 ha 0.005 0.0002 0.014 0.0011

Sagebrush height 661.4 ha 0.19 0.00 0.13 0.00

Distance to cropland Km 6.31 0.04 3.29 0.11

Variety of edge types 661.4 ha 6.97 0.04 10.11 0.09

Variety of land cover types 661.4 ha 5.02 0.02 7.31 0.07

Distance to spring Km 5.09 0.04 2.94 0.07

Distance to water body Km 4.32 0.04 1.79 0.04

Distance to wet meadow Km 11.99 0.07 3.59 0.09

Topographic position index 2010 m -0.56 0.51 -11.91 0.71

North SWIP Spring Annual grass 661.4 ha 0.003 0.0004 0.000 0.0000

Big sagebrush 661.4 ha 0.064 0.0006 0.116 0.0019

Cropland 661.4 ha 0.007 0.0004 0.017 0.0016

Forest 661.4 ha 0.010 0.0006 0.016 0.0007

Herbaceous 661.4 ha 0.064 0.0003 0.102 0.0013

Non-sagebrush shrub 661.4 ha 0.058 0.0004 0.074 0.0012

Other sagebrush 661.4 ha 0.022 0.0001 0.035 0.0007

Pinyon-juniper 8.7 ha 0.111 0.0027 0.026 0.0022

Riparian 661.4 ha 0.009 0.0003 0.007 0.0004

Variety of edge types 661.4 ha 7.26 0.05 9.23 0.12

Variety of land cover types 8.7 ha 2.17 0.02 2.55 0.04

Distance to nearest stream Km 0.31 0.01 0.18 0.01

Distance to spring Km 5.38 0.06 4.26 0.15

Distance to water body Km 4.45 0.04 3.98 0.08

Distance to wet meadow Km 9.97 0.10 12.57 0.22

Elevation Km 2.04 0.00 2.24 0.01

Topographic position index 2010 m 0.66 0.74 19.33 1.74

Summer Annual grass 8.7 ha 0.003 0.0007 0.000 0.0000

Big sagebrush 661.4 ha 0.063 0.0005 0.136 0.0017

Forest 661.4 ha 0.010 0.0005 0.026 0.0008

Herbaceous 661.4 ha 0.064 0.0003 0.126 0.0011

Non-sagebrush shrub 661.4 ha 0.057 0.0004 0.101 0.0012

Other sagebrush 61.5 ha 0.022 0.0002 0.040 0.0005

Pinyon-juniper 8.7 ha 0.098 0.0020 0.016 0.0016

Riparian 8.7 ha 0.010 0.0006 0.026 0.0024

Sagebrush height 661.4 ha 0.25 0.00 0.28 0.00

Distance to cropland Km 4.11 0.04 6.65 0.10

Variety of edge types 661.4 ha 7.32 0.05 11.42 0.08

Variety of land cover types 8.7 ha 2.16 0.01 3.01 0.04

Distance to spring Km 5.36 0.05 1.47 0.07

Distance to water body Km 4.50 0.04 3.26 0.06

Distance to wet meadow Km 10.00 0.09 10.30 0.13

Elevation Km 2.04 0.00 2.44 0.01

Topographic position index 2010 m 0.25 0.65 10.36 1.99

Winter Bare ground 61.5 ha 0.501 0.0035 0.673 0.0026

Big sagebrush 661.4 ha 0.063 0.0006 0.076 0.0016

Forest 661.4 ha 0.010 0.0006 0.002 0.0003

Herbaceous 661.4 ha 0.064 0.0003 0.071 0.0005

Non-sagebrush shrub 661.4 ha 0.057 0.0004 0.047 0.0005

Other sagebrush 661.4 ha 0.022 0.0002 0.031 0.0003

Riparian 661.4 ha 0.009 0.0003 0.001 0.0001

Sagebrush height 8.7 ha 0.24 0.00 0.25 0.00

Distance to cropland Km 4.08 0.04 3.37 0.07

Variety of edge types 661.4 ha 7.22 0.06 4.70 0.09

Distance to water body Km 4.43 0.04 7.05 0.09

Distance to wet meadow Km 9.98 0.10 17.51 0.18

Topographic position index 2010 m -0.93 0.74 -6.34 0.67

South SWIP Spring Big sagebrush 661.4 ha 0.069 0.0006 0.080 0.0009

Forest 661.4 ha 0.008 0.0004 0.002 0.0004

Herbaceous 661.4 ha 0.070 0.0004 0.054 0.0007

Non-sagebrush shrub 661.4 ha 0.059 0.0003 0.057 0.0005

Pinyon-juniper 61.5 ha 0.151 0.0022 0.031 0.0014

Riparian 8.7 ha 0.012 0.0006 0.007 0.0011

Distance to cropland Km 7.63 0.06 4.09 0.15

Variety of edge types 661.4 ha 6.73 0.04 6.44 0.08

Variety of land cover types 661.4 ha 5.06 0.02 6.33 0.06

Distance to intermittent stream Km 0.30 0.00 0.16 0.01

Distance to spring Km 5.69 0.06 4.56 0.12

Distance to water body Km 3.37 0.03 2.60 0.04

Roughness index 1 ha 0.16 0.00 0.10 0.00

Topographic position index 2010 m -0.59 0.54 -4.57 0.46

Summer Big sagebrush 661.4 ha 0.068 0.0004 0.087 0.0002

Forest 661.4 ha 0.008 0.0003 0.001 0.0001

Herbaceous 661.4 ha 0.070 0.0003 0.043 0.0002

Non-sagebrush shrub 661.4 ha 0.059 0.0002 0.052 0.0002

Pinyon-juniper 61.5 ha 0.151 0.0017 0.014 0.0003

Riparian 661.4 ha 0.010 0.0001 0.014 0.0001

Distance to cropland Km 7.49 0.04 1.26 0.02

Variety of edge types 61.5 ha 2.38 0.02 3.50 0.03

Distance to nearest stream Km 0.28 0.00 0.10 0.00

Distance to spring Km 5.72 0.04 2.26 0.02

Distance to water body Km 3.40 0.02 2.38 0.01

Distance to wet meadow Km 11.30 0.06 4.94 0.03

Elevation Km 2.09 0.00 1.92 0.00

Topographic position index 2010 m -0.38 0.40 -10.59 0.19

Toiyabe Spring Annual grass 661.4 ha 0.003 0.0003 0.006 0.0006

Herbaceous 661.4 ha 0.055 0.0003 0.083 0.0010

Non-sagebrush shrub 8.7 ha 0.043 0.0003 0.048 0.0011

Other sagebrush 661.4 ha 0.033 0.0001 0.045 0.0003

Pinyon-juniper 61.5 ha 0.049 0.0011 0.001 0.0002

Riparian 661.4 ha 0.013 0.0002 0.020 0.0007

Sagebrush height 661.4 ha 0.370 0.0014 0.460 0.0018

Distance to cropland Km 4.53 0.03 2.95 0.04

Variety of edge types 661.4 ha 7.27 0.04 8.82 0.10

Distance to spring Km 3.33 0.03 1.70 0.02

Distance to water body Km 6.58 0.04 4.95 0.06

Distance to wet meadow Km 12.13 0.06 9.31 0.10

Summer Annual grass 661.4 ha 0.004 0.0003 0.004 0.0004

Big sagebrush 661.4 ha 7.542 0.0326 10.405 0.0666

Herbaceous 661.4 ha 5.455 0.0310 10.469 0.0989

Non-sagebrush shrub 661.4 ha 4.284 0.0201 5.778 0.0640

Other sagebrush 661.4 ha 3.241 0.0135 4.338 0.0266

Pinyon-juniper 61.5 ha 0.049 0.0010 0.001 0.0001

Riparian 661.4 ha 0.013 0.0002 0.028 0.0008

Distance to cropland Km 4.54 0.03 3.52 0.04

Variety of edge types 661.4 ha 7.23 0.04 10.87 0.05

Distance to perennial stream Km 6.09 0.05 1.95 0.03

Distance to water body Km 6.58 0.03 5.77 0.05

Distance to wet meadow Km 12.12 0.06 8.01 0.11

Roughness index 1 ha 0.17 0.00 0.22 0.00

Topographic position index 510 m -0.08 0.17 -6.39 0.46

Winter Annual grass 661.4 ha 0.003 0.0003 0.003 0.0006

Big sagebrush 661.4 ha 0.076 0.0004 0.086 0.0006

Herbaceous 661.4 ha 0.054 0.0004 0.060 0.0007

Non-sagebrush shrub 661.4 ha 0.043 0.0002 0.032 0.0005

Other sagebrush 661.4 ha 0.032 0.0002 0.046 0.0002

Pinyon-juniper 61.5 ha 0.049 0.0012 0.001 0.0002

Riparian 661.4 ha 0.012 0.0002 0.009 0.0005

Distance to cropland Km 4.53 0.04 2.52 0.05

Variety of edge types 661.4 ha 7.19 0.05 6.95 0.09

Distance to spring Km 3.38 0.03 1.29 0.03

Distance to water body Km 6.60 0.04 4.12 0.06

Distance to wet meadow Km 12.05 0.07 10.23 0.11

Topographic position index 510 m 0.07 0.20 1.76 0.34

Tuscarora Spring Annual grass 661.4 ha 0.015 0.0004 0.004 0.0001

Bare ground 661.4 ha 0.463 0.0015 0.366 0.0022

Forest 61.5 ha 0.015 0.0006 0.003 0.0003

Herbaceous 8.7 ha 0.179 0.0010 0.225 0.0019

Non-sagebrush shrub 8.7 ha 0.058 0.0005 0.050 0.0011

Other sagebrush 61.5 ha 0.040 0.0001 0.044 0.0003

Riparian 8.7 ha 0.026 0.0008 0.017 0.0012

Distance to cropland Km 0.40 0.00 0.52 0.01

Variety of edge types 661.4 ha 9.98 0.04 10.57 0.06

Variety of land cover types 8.7 ha 2.69 0.01 2.50 0.03

Distance to nearest stream Km 0.20 0.00 0.34 0.01

Distance to spring Km 2.00 0.02 1.70 0.02

Distance to water body Km 3.22 0.02 3.63 0.03

Distance to wet meadow Km 13.34 0.09 6.70 0.08

Elevation Km 1.92 0.00 1.90 0.00

Roughness index 1 ha 0.19 0.00 0.16 0.00

Topographic position index 510 m 0.17 0.21 4.49 0.38

Summer Annual grass 661.4 ha 0.015 0.0004 0.002 0.0001

Bare ground 661.4 ha 0.462 0.0013 0.308 0.0018

Cropland 661.4 ha 0.026 0.0008 0.127 0.0035

Forest 61.5 ha 0.015 0.0006 0.006 0.0005

Herbaceous 661.4 ha 0.179 0.0007 0.215 0.0012

Other sagebrush 661.4 ha 0.040 0.0001 0.036 0.0002

Riparian 661.4 ha 0.023 0.0003 0.044 0.0007

Variety of edge types 661.4 ha 10.04 0.03 11.59 0.04

Variety of land cover types 61.5 ha 4.36 0.02 4.89 0.02

Distance to perennial stream Km 2.08 0.02 1.53 0.03

Distance to spring Km 2.06 0.02 1.84 0.02

Distance to water body Km 3.19 0.02 3.19 0.03

Distance to wet meadow Km 13.23 0.08 7.52 0.06

Roughness index 1 ha 0.19 0.00 0.16 0.00

Topographic position index 510 m -0.12 0.19 1.08 0.34

Winter Annual grass 661.4 ha 0.015 0.0005 0.001 0.0002

Forest 661.4 ha 0.015 0.0006 0.003 0.0005

Herbaceous 661.4 ha 0.180 0.0010 0.193 0.0012

Non-sagebrush shrub 61.5 ha 0.059 0.0006 0.034 0.0010

Other sagebrush 661.4 ha 0.040 0.0002 0.049 0.0003

Riparian 61.5 ha 0.024 0.0007 0.011 0.0011

Sagebrush height 661.4 ha 0.340 0.0014 0.380 0.0016

Distance to cropland Km 2.330 0.0236 1.530 0.0427

Variety of land cover types 61.5 ha 4.42 0.02 3.60 0.04

Distance to nearest stream Km 0.20 0.00 0.34 0.01

Distance to spring Km 2.02 0.02 1.42 0.03

Distance to water body Km 3.20 0.03 3.29 0.03

Distance to wet meadow Km 13.23 0.12 7.54 0.07

Elevation Km 1.92 0.00 1.86 0.00

Roughness index 1 ha 0.20 0.00 0.13 0.00

Topographic position index 2010 m 0.13 0.65 -5.60 0.91

Virginia Spring Annual grass 661.4 ha 0.040 0.0016 0.330 0.0059

Bare ground 661.4 ha 0.420 0.0031 0.388 0.0017

Cropland 661.4 ha 0.013 0.0010 0.000 0.0002

Forest 661.4 ha 0.001 0.0001 0.002 0.0004

Herbaceous 661.4 ha 0.176 0.0018 0.321 0.0014

Non-sagebrush shrub 8.7 ha 0.034 0.0006 0.056 0.0019

Other sagebrush 661.4 ha 0.028 0.0004 0.032 0.0005

Riparian 61.5 ha 0.005 0.0003 0.003 0.0003

Sagebrush height 661.4 ha 0.25 0.00 0.24 0.00

Variety of land cover types 8.7 ha 2.53 0.02 3.28 0.03

Distance to nearest stream Km 0.38 0.01 0.38 0.01

Distance to water body Km 2.59 0.03 2.42 0.05

Distance to wet meadow Km 5.28 0.06 3.89 0.06

Roughness index 1 ha 0.19 0.00 0.22 0.00

Topographic position index 2010 m 2.54 1.07 39.90 2.84

Summer Annual grass 661.4 ha 0.037 0.003 0.312 0.008

Bare ground 661.4 ha 0.418 0.006 0.385 0.002

Forest 661.4 ha 0.001 0.000 0.004 0.001

Herbaceous 661.4 ha 0.169 0.003 0.342 0.002

Non-sagebrush shrub 661.4 ha 0.033 0.001 0.068 0.002

Other sagebrush 8.7 ha 0.028 0.001 0.020 0.001

Riparian 661.4 ha 0.004 0.000 0.007 0.000

Distance to cropland Km 3.70 0.08 5.66 0.08

Variety of land cover types 8.7 ha 2.45 0.04 3.46 0.06

Distance to perennial stream Km 5.79 0.13 4.18 0.11

Distance to water body Km 2.52 0.06 2.00 0.07

Distance to wet meadow Km 5.16 0.10 3.62 0.08

Roughness index 1 ha 0.19 0.00 0.20 0.00

Topographic position index 510 m -0.46 0.65 3.71 1.12

**Table S5.** Meta-analysis results, including parameter estimates (85% confidence intervals) and I^2 for the habitat features found important for greater sage-grouse (*Centrocercus urophasianus*) in resource selection function (RSF) modeling in Nevada and northeastern California. Results are presented across all sites, as well as across those sites in the northern and southern hydrographic boundaries separately.

Variable Season Model Sites Estimate (85% CI) I^2^ (%)

Annual grass Spring Combined 5 -0.33 (-1.33 – 0.66) 99.62 (99.52 – 99.71)

Annual grass Spring North 3 0.23 (-0.83 – 1.29) 99.78 (99.7 – 99.83)

Annual grass Spring South 2 -1.24 (-3.31 – 0.83) 97.4 (93.46 – 98.96)

Annual grass Summer Combined 5 -0.6 (-1.8 – 0.59) 99.95 (99.94 – 99.95)

Annual grass Summer North 3 -0.36 (-2.35 – 1.64) 99.89 (99.86 – 99.91)

Annual grass Summer South 2 -0.91 (-2.08 – 0.27) 82.55 (26.74 – 95.84)

Annual grass Winter Combined 4 -0.88 (-2.37 – 0.62) 99.57 (99.41 – 99.68)

Annual grass Winter North 3 -1.15 (-3.2 – 0.89) 99.7 (99.59 – 99.79)

Annual grass Winter South 1 -0.05 (-0.09 – -0.02) NA

Bare ground Spring Combined 4 -0.21 (-0.68 – 0.25) 99.89 (99.86 – 99.91)

Bare ground Spring North 4 -0.21 (-0.68 – 0.25) 99.89 (99.86 – 99.91)

Bare ground Summer Combined 4 -0.4 (-1.5 – 0.7) 99.89 (99.86 – 99.91)

Bare ground Summer North 4 -0.4 (-1.5 – 0.7) 99.89 (99.86 – 99.91)

Bare ground Winter Combined 3 0.86 (0.32 – 1.4) 99.49 (99.24 – 99.65)

Bare ground Winter North 2 0.5 (0.22 – 0.77) 96.47 (90.37 – 98.71)

Bare ground Winter South 1 1.57 (1.5 – 1.64) NA

Big sagebrush Spring Combined 5 0.27 (-0.01 – 0.54) 99.65 (99.55 – 99.73)

Big sagebrush Spring North 2 -0.16 (-0.44 – 0.13) 99.59 (99.32 – 99.76)

Big sagebrush Spring South 3 0.55 (0.4 – 0.7) 97.66 (95.53 – 98.78)

Big sagebrush Summer Combined 6 0.63 (0.36 – 0.91) 99.71 (99.65 – 99.77)

Big sagebrush Summer North 2 0.25 (-0.53 – 1.02) 99.82 (99.74 – 99.88)

Big sagebrush Summer South 4 0.83 (0.73 – 0.93) 95.16 (90.57 – 97.52)

Big sagebrush Winter Combined 5 -0.01 (-0.26 – 0.24) 99.02 (98.61 – 99.31)

Big sagebrush Winter North 2 -0.22 (-0.7 – 0.26) 99.18 (98.44 – 99.57)

Big sagebrush Winter South 3 0.12 (-0.17 – 0.42) 99.2 (98.74 – 99.49)

Cropland Spring Combined 3 0.05 (-0.86 – 0.97) 96.12 (91.73 – 98.18)

Cropland Spring North 1 -1.28 (-1.73 – -0.84) NA

Cropland Spring South 2 0.67 (0.55 – 0.78) 92.51 (74.65 – 97.79)

Cropland Summer Combined 1 0.73 (0.7 – 0.76) NA

Cropland Summer North 1 0.73 (0.7 – 0.76) NA

Cropland Winter Combined 2 -0.13 (-1.03 – 0.76) 95.86 (88.18 – 98.55)

Cropland Winter North 2 -0.13 (-1.03 – 0.76) 95.86 (88.18 – 98.55)

Forest Spring Combined 6 -3.25 (-6.21 – -0.28) 99.45 (99.29 – 99.58)

Forest Spring North 3 -0.67 (-1.58 – 0.24) 99.56 (99.36 – 99.7)

Forest Spring South 3 -5.85 (-11.32 – -0.37) 99.49 (99.25 – 99.66)

Forest Summer Combined 8 -0.45 (-0.7 – -0.2) 98.82 (98.43 – 99.11)

Forest Summer North 4 -0.42 (-0.92 – 0.08) 99.38 (99.12 – 99.56)

Forest Summer South 4 -0.48 (-0.66 – -0.29) 94.59 (89.22 – 97.28)

Forest Winter Combined 4 -0.42 (-0.79 – -0.04) 96.99 (94.64 – 98.31)

Forest Winter North 1 -0.81 (-0.88 – -0.73) NA

Forest Winter South 3 -0.27 (-0.72 – 0.17) 92.92 (82.65 – 97.11)

Herbaceous Spring Combined 9 0.7 (0.18 – 1.22) 99.74 (99.7 – 99.78)

Herbaceous Spring North 3 1.44 (0.23 – 2.66) 99.41 (99.11 – 99.61)

Herbaceous Spring South 6 0.33 (-0.11 – 0.78) 99.8 (99.76 – 99.83)

Herbaceous Summer Combined 10 1.36 (0.44 – 2.27) 100 (100 – 100)

Herbaceous Summer North 4 2 (-0.22 – 4.23) 100 (100 – 100)

Herbaceous Summer South 6 0.92 (0.34 – 1.51) 99.77 (99.73 – 99.81)

Herbaceous Winter Combined 6 0.27 (-0.04 – 0.57) 99.63 (99.53 – 99.7)

Herbaceous Winter North 3 0.45 (-0.03 – 0.93) 99.63 (99.47 – 99.74)

Herbaceous Winter South 3 0.08 (-0.33 – 0.5) 99.62 (99.46 – 99.73)

Non-sagebrush shrub Spring Combined 10 -0.23 (-0.57 – 0.12) 99.84 (99.82 – 99.86)

Non-sagebrush shrub Spring North 4 -0.08 (-0.75 – 0.59) 99.92 (99.9 – 99.93)

Non-sagebrush shrub Spring South 6 -0.32 (-0.72 – 0.08) 99.76 (99.71 – 99.8)

Non-sagebrush shrub Summer Combined 7 0.47 (0.16 – 0.78) 99.59 (99.5 – 99.67)

Non-sagebrush shrub Summer North 2 0.88 (0.79 – 0.98) 35.03 (NA – NA)

Non-sagebrush shrub Summer South 5 0.29 (-0.08 – 0.66) 99.64 (99.54 – 99.72)

Non-sagebrush shrub Winter Combined 7 -0.48 (-0.83 – -0.13) 99.63 (99.55 – 99.7)

Non-sagebrush shrub Winter North 3 -0.64 (-1.52 – 0.23) 99.86 (99.82 – 99.89)

Non-sagebrush shrub Winter South 4 -0.33 (-0.43 – -0.23) 74.87 (30.21 – 90.95)

Other sagebrush Spring Combined 7 1.04 (0.79 – 1.29) 99.53 (99.42 – 99.63)

Other sagebrush Spring North 3 0.84 (0.61 – 1.06) 97.57 (95.31 – 98.74)

Other sagebrush Spring South 4 1.19 (0.8 – 1.59) 99.73 (99.65 – 99.79)

Other sagebrush Summer Combined 8 0.5 (0.16 – 0.85) 99.7 (99.65 – 99.75)

Other sagebrush Summer North 4 0.46 (-0.26 – 1.18) 99.85 (99.81 – 99.88)

Other sagebrush Summer South 4 0.54 (0.34 – 0.74) 98.86 (98.26 – 99.25)

Other sagebrush Winter Combined 7 0.86 (0.52 – 1.2) 99.51 (99.39 – 99.61)

Other sagebrush Winter North 3 0.4 (-0.1 – 0.9) 99.6 (99.43 – 99.72)

Other sagebrush Winter South 4 1.2 (0.9 – 1.5) 96.92 (94.48 – 98.28)

Pinyon-juniper Spring Combined 6 -2.37 (-3.5 – -1.24) 99.55 (99.43 – 99.65)

Pinyon-juniper Spring North 1 -0.67 (-0.71 – -0.63) NA

Pinyon-juniper Spring South 5 -2.71 (-3.96 – -1.47) 99.57 (99.44 – 99.67)

Pinyon-juniper Summer Combined 6 -2.23 (-3.15 – -1.32) 99.28 (99.05 – 99.46)

Pinyon-juniper Summer North 1 -1.06 (-1.14 – -0.98) NA

Pinyon-juniper Summer South 5 -2.47 (-3.51 – -1.43) 99.38 (99.16 – 99.54)

Pinyon-juniper Winter Combined 2 -3.84 (-6.22 – -1.45) 99.16 (98.39 – 99.56)

Pinyon-juniper Winter South 2 -3.84 (-6.22 – -1.45) 99.16 (98.39 – 99.56)

Riparian Spring Combined 8 -0.28 (-0.56 – 0) 99.57 (99.47 – 99.65)

Riparian Spring North 3 -0.38 (-0.47 – -0.28) 96.58 (92.91 – 98.35)

Riparian Spring South 5 -0.22 (-0.68 – 0.24) 99.64 (99.54 – 99.72)

Riparian Summer Combined 8 -0.1 (-0.34 – 0.15) 99.59 (99.5 – 99.66)

Riparian Summer North 3 -0.38 (-0.73 – -0.02) 99.01 (98.38 – 99.4)

Riparian Summer South 5 0.07 (-0.23 – 0.37) 99.57 (99.45 – 99.67)

Riparian Winter Combined 5 -0.92 (-1.67 – -0.18) 99.08 (98.71 – 99.35)

Riparian Winter North 2 -0.42 (-0.63 – -0.2) 96.05 (88.87 – 98.6)

Riparian Winter South 3 -1.27 (-2.51 – -0.03) 99.34 (98.99 – 99.57)

Wet meadow Spring Combined 1 -0.39 (-0.47 – -0.31) NA

Wet meadow Spring North 1 -0.39 (-0.47 – -0.31) NA

Wet meadow Summer Combined 1 -0.35 (-0.4 – -0.3) NA

Wet meadow Summer North 1 -0.35 (-0.4 – -0.3) NA

Sagebrush height Spring Combined 5 0.13 (-0.55 – 0.81) 99.91 (99.9 – 99.93)

Sagebrush height Spring North 2 0.14 (-0.59 – 0.87) 99.56 (99.26 – 99.74)

Sagebrush height Spring South 3 0.12 (-1.05 – 1.28) 99.94 (99.93 – 99.95)

Sagebrush height Summer Combined 5 0.01 (-0.15 – 0.18) 98.12 (97.1 – 98.78)

Sagebrush height Summer North 1 0.08 (0.03 – 0.13) NA

Sagebrush height Summer South 4 0 (-0.21 – 0.2) 98.49 (97.6 – 99.05)

Sagebrush height Winter Combined 3 0.43 (-0.28 – 1.13) 99.63 (99.47 – 99.74)

Sagebrush height Winter North 1 1.36 (1.31 – 1.41) NA

Sagebrush height Winter South 3 -0.04 (-0.42 – 0.34) 96.47 (90.37 – 98.71)

Distance to cropland Spring Combined 3 -1.06 (-1.28 – -0.85) 97.29 (94.66 – 98.63)

Distance to cropland Spring South 3 -1.06 (-1.28 – -0.85) 97.29 (94.66 – 98.63)

Distance to cropland Summer Combined 5 -0.84 (-1.45 – -0.23) 99.68 (99.59 – 99.75)

Distance to cropland Summer North 1 -0.43 (-0.61 – -0.24) NA

Distance to cropland Summer South 4 -0.94 (-1.7 – -0.18) 99.76 (99.69 – 99.81)

Distance to cropland Winter Combined 4 -0.72 (-0.93 – -0.52) 98.73 (98.04 – 99.18)

Distance to cropland Winter North 1 -0.46 (-0.5 – -0.43) NA

Distance to cropland Winter South 3 -0.81 (-1.03 – -0.59) 97.05 (94.07 – 98.53)

Distance to cropland_Exp. Spring Combined 4 0.22 (-0.21 – 0.66) 99.84 (99.8 – 99.87)

Distance to cropland_Exp. Spring North 3 -0.07 (-0.24 – 0.1) 99.27 (98.86 – 99.53)

Distance to cropland_Exp. Spring South 1 1.1 (1.06 – 1.14) NA

Distance to cropland_Exp. Summer Combined 3 0.37 (-1.05 – 1.8) 99.8 (99.73 – 99.85)

Distance to cropland_Exp. Summer North 1 -1.36 (-1.61 – -1.1) NA

Distance to cropland_Exp. Summer South 2 1.23 (0.01 – 2.44) 99.88 (99.83 – 99.91)

Variety of edge types Spring Combined 5 -0.13 (-0.54 – 0.28) 99.84 (99.81 – 99.87)

Variety of edge types Spring North 1 0.04 (0.01 – 0.06) NA

Variety of edge types Spring South 4 -0.17 (-0.7 – 0.35) 99.88 (99.86 – 99.9)

Variety of edge types Summer Combined 6 0.41 (-0.1 – 0.92) 99.85 (99.83 – 99.88)

Variety of edge types Summer South 6 0.41 (-0.1 – 0.92) 99.85 (99.83 – 99.88)

Variety of edge types Winter Combined 2 -0.83 (-1.09 – -0.57) 98.76 (97.41 – 99.41)

Variety of edge types Winter South 2 -0.83 (-1.09 – -0.57) 98.76 (97.41 – 99.41)

Variety of land cover types Spring Combined 9 0.03 (-0.3 – 0.36) 99.84 (99.82 – 99.86)

Variety of land cover types Spring North 4 -0.28 (-0.67 – 0.11) 99.62 (99.5 – 99.72)

Variety of land cover types Spring South 5 0.28 (-0.2 – 0.76) 99.85 (99.82 – 99.87)

Variety of land cover types Summer Combined 7 0.4 (0.07 – 0.74) 99.62 (99.53 – 99.69)

Variety of land cover types Summer North 3 0.16 (-0.41 – 0.74) 99.77 (99.69 – 99.83)

Variety of land cover types Summer South 4 0.58 (0.16 – 1) 99.08 (98.63 – 99.38)

Variety of land cover types Winter Combined 5 -0.46 (-0.62 – -0.3) 97.76 (96.46 – 98.58)

Variety of land cover types Winter North 3 -0.55 (-0.8 – -0.31) 98.55 (97.47 – 99.17)

Variety of land cover types Winter South 2 -0.32 (-0.41 – -0.22) 54.52 (0 – 88.87)

Distance to nearest stream Spring Combined 3 -0.12 (-0.69 – 0.45) 99.89 (99.86 – 99.91)

Distance to nearest stream Spring North 1 0.66 (0.64 – 0.69) NA

Distance to nearest stream Spring South 2 -0.51 (-0.6 – -0.42) 85.41 (41.1 – 96.39)

Distance to nearest stream Summer Combined 1 -0.96 (-1.05 – -0.87) NA

Distance to nearest stream Summer South 1 -0.96 (-1.05 – -0.87) NA

Distance to nearest stream Winter Combined 2 0.02 (-1.08 – 1.12) 99.75 (99.61 – 99.84)

Distance to nearest stream Winter North 1 0.79 (0.75 – 0.82) NA

Distance to nearest stream Winter South 1 -0.74 (-0.85 – -0.64) NA

Distance to nearest stream_Exp. Spring Combined 1 -0.68 (-0.75 – -0.61) NA

Distance to nearest stream_Exp. Spring North 1 -0.68 (-0.75 – -0.61) NA

Distance to intermittent stream Spring Combined 2 0.17 (-0.38 – 0.72) 99.84 (99.77 – 99.89)

Distance to intermittent stream Spring North 1 -0.21 (-0.24 – -0.19) NA

Distance to intermittent stream Spring South 1 0.55 (0.51 – 0.58) NA

Distance to intermittent stream_Exp. Spring Combined 1 0.38 (0.34 – 0.41) NA

Distance to intermittent stream_Exp. Spring South 1 0.38 (0.34 – 0.41) NA

Distance to intermittent stream_Exp. Summer Combined 1 -0.25 (-0.28 – -0.21) NA

Distance to intermittent stream_Exp. Summer South 1 -0.25 (-0.28 – -0.21) NA

Distance to intermittent stream_Exp. Winter Combined 1 -0.64 (-0.74 – -0.54) NA

Distance to intermittent stream_Exp. Winter South 1 -0.64 (-0.74 – -0.54) NA

Distance to perennial stream Spring Combined 1 -1.14 (-1.24 – -1.05) NA

Distance to perennial stream Spring South 1 -1.14 (-1.24 – -1.05) NA

Distance to perennial stream Summer Combined 3 -1.33 (-1.64 – -1.01) 93.2 (83.48 – 97.2)

Distance to perennial stream Summer North 1 -0.93 (-1.21 – -0.65) NA

Distance to perennial stream Summer South 2 -1.49 (-1.79 – -1.18) 94.43 (82.7 – 98.21)

Distance to perennial stream Winter Combined 1 -0.47 (-0.53 – -0.41) NA

Distance to perennial stream Winter North 1 -0.47 (-0.53 – -0.41) NA

Distance to perennial stream_Exp. Spring Combined 1 -0.29 (-0.32 – -0.25) NA

Distance to perennial stream_Exp. Spring North 1 -0.29 (-0.32 – -0.25) NA

Distance to perennial stream_Exp. Summer Combined 1 0.16 (-0.61 – 0.93) 99.84 (99.77 – 99.89)

Distance to perennial stream_Exp. Summer North 1 0.16 (-0.61 – 0.93) 99.84 (99.77 – 99.89)

Distance to perennial stream_Exp. Winter Combined 1 -0.43 (-0.49 – -0.37) NA

Distance to perennial stream_Exp. Winter North 1 -0.43 (-0.49 – -0.37) NA

Distance to spring Spring Combined 5 -0.63 (-1.09 – -0.16) 99.81 (99.77 – 99.84)

Distance to spring Spring North 2 0.03 (-0.12 – 0.18) 98.41 (96.48 – 99.28)

Distance to spring Spring South 3 -1.07 (-1.52 – -0.61) 99.71 (99.6 – 99.79)

Distance to spring Summer Combined 4 -0.81 (-1.37 – -0.25) 99.62 (99.49 – 99.71)

Distance to spring Summer North 1 0.14 (0.1 – 0.17) NA

Distance to spring Summer South 3 -1.13 (-1.57 – -0.68) 96.85 (93.6 – 98.45)

Distance to spring Winter Combined 2 -1.46 (-2.28 – -0.65) 99.73 (99.59 – 99.83)

Distance to spring Winter North 1 -0.9 (-0.95 – -0.85) NA

Distance to spring Winter South 1 -2.03 (-2.1 – -1.97) NA

Distance to spring_Exp. Spring Combined 3 -0.11 (-0.16 – -0.06) 86.03 (59.44 – 95.19)

Distance to spring_Exp. Spring North 1 -0.18 (-0.21 – -0.15) NA

Distance to spring_Exp. Spring South 2 -0.07 (-0.1 – -0.05) 0 (0.00 – 0.00)

Distance to spring_Exp. Summer Combined 4 0.59 (-0.01 – 1.18) 99.87 (99.84 – 99.89)

Distance to spring_Exp. Summer North 2 0.34 (-1.02 – 1.71) 99.94 (99.92 – 99.95)

Distance to spring_Exp. Summer South 2 0.83 (0.63 – 1.02) 97.19 (92.78 – 98.9)

Distance to spring_Exp. Winter Combined 3 0 (-0.45 – 0.45) 99.36 (99.03 – 99.58)

Distance to spring_Exp. Winter North 2 -0.21 (-0.8 – 0.38) 99.53 (99.2 – 99.73)

Distance to spring_Exp. Winter South 1 0.4 (0.33 – 0.48) NA

Distance to wet meadow Spring Combined 4 -0.8 (-1.54 – -0.07) 99.91 (99.9 – 99.93)

Distance to wet meadow Spring North 2 -1.39 (-1.8 – -0.98) 97.77 (94.61 – 99.07)

Distance to wet meadow Spring South 2 -0.22 (-1.49 – 1.06) 99.95 (99.94 – 99.96)

Distance to wet meadow Summer Combined 5 -1.38 (-2.23 – -0.54) 99.79 (99.74 – 99.83)

Distance to wet meadow Summer North 2 -1 (-1.89 – -0.11) 98.06 (95.48 – 99.16)

Distance to wet meadow Summer South 3 -1.64 (-3.04 – -0.25) 99.88 (99.85 – 99.9)

Distance to wet meadow Winter Combined 3 -0.77 (-1.18 – -0.36) 99.31 (98.93 – 99.55)

Distance to wet meadow Winter North 1 -1.29 (-1.35 – -1.24) NA

Distance to wet meadow Winter South 2 -0.51 (-0.78 – -0.23) 98.02 (95.36 – 99.15)

Distance to wet meadow_Exp. Spring Combined 3 0.53 (0.21 – 0.84) 99.53 (99.32 – 99.68)

Distance to wet meadow_Exp. Spring North 1 0.13 (0.11 – 0.15) NA

Distance to wet meadow_Exp. Spring South 2 0.73 (0.51 – 0.95) 96.41 (90.16 – 98.69)

Distance to wet meadow_Exp. Summer Combined 2 -0.3 (-0.4 – -0.21) 86.46 (46.29 – 96.59)

Distance to wet meadow_Exp. Summer North 1 -0.37 (-0.42 – -0.32) NA (NA – NA)

Distance to wet meadow_Exp. Summer South 1 -0.24 (-0.28 – -0.19) NA (NA – NA)

Distance to wet meadow_Exp. Winter Combined 1 -1.67 (-1.73 – -1.62) NA

Distance to wet meadow_Exp. Winter South 1 -1.67 (-1.73 – -1.62) NA

Distance to water body Spring Combined 6 -0.27 (-0.64 – 0.11) 99.68 (99.61 – 99.74)

Distance to water body Spring North 2 -0.06 (-0.48 – 0.36) 99.04 (98.11 – 99.51)

Distance to water body Spring South 4 -0.37 (-0.91 – 0.17) 99.79 (99.73 – 99.83)

Distance to water body Summer Combined 8 -0.74 (-1.07 – -0.41) 98.11 (97.38 – 98.64)

Distance to water body Summer North 3 -0.71 (-1.04 – -0.39) 94.03 (85.95 – 97.46)

Distance to water body Summer South 5 -0.75 (-1.28 – -0.23) 98.78 (98.22 – 99.16)

Distance to water body Winter Combined 4 -0.62 (-0.86 – -0.37) 98.94 (98.41 – 99.3)

Distance to water body Winter North 1 -0.37 (-0.43 – -0.3) NA

Distance to water body Winter South 3 -0.7 (-1 – -0.4) 98.75 (97.88 – 99.27)

Distance to water body_Exp. Spring Combined 2 -0.29 (-0.84 – 0.26) 99.89 (99.86 – 99.92)

Distance to water body_Exp. Spring North 2 -0.29 (-0.84 – 0.26) 99.89 (99.86 – 99.92)

Distance to water body_Exp. Summer Combined 2 0.33 (-0.44 – 1.11) 99.87 (99.82 – 99.91)

Distance to water body_Exp. Summer North 1 -0.2 (-0.23 – -0.18) NA

Distance to water body_Exp. Summer South 1 0.87 (0.82 – 0.92) NA

Distance to water body_Exp. Winter Combined 2 -0.89 (-1.09 – -0.69) 97.81 (94.76 – 99.09)

Distance to water body_Exp. Winter North 1 -0.75 (-0.79 – -0.71) NA

Distance to water body_Exp. Winter South 1 -1.03 (-1.08 – -0.99) NA

Elevation Spring Combined 4 -0.33 (-0.84 – 0.17) 99.86 (99.83 – 99.88)

Elevation Spring North 3 -0.47 (-1.13 – 0.19) 99.9 (99.88 – 99.92)

Elevation Spring South 1 0.07 (0.03 – 0.11) NA

Elevation Summer Combined 3 0.51 (-0.19 – 1.2) 99.69 (99.57 – 99.78)

Elevation Summer North 1 1.33 (1.27 – 1.4) NA

Elevation Summer South 2 0.09 (-0.52 – 0.7) 99.46 (99.04 – 99.69)

Elevation Winter Combined 3 -0.15 (-0.62 – 0.33) 99.4 (99.09 – 99.6)

Elevation Winter North 3 -0.15 (-0.62 – 0.33) 99.4 (99.09 – 99.6)

Roughness Spring Combined 6 -0.44 (-0.75 – -0.13) 99.7 (99.63 – 99.75)

Roughness Spring North 4 -0.26 (-0.91 – 0.38) 99.87 (99.84 – 99.9)

Roughness Spring South 2 -0.57 (-0.88 – -0.26) 99.25 (98.91 – 99.48)

Roughness Summer Combined 4 -0.76 (-1.14 – -0.39) 99.74 (99.69 – 99.79)

Roughness Summer North 3 -0.92 (-1.47 – -0.38) 99.77 (99.7 – 99.82)

Roughness Summer South 1 -0.44 (-0.47 – -0.41) NA

Roughness Winter Combined 4 -0.63 (-1.2 – -0.07) 99.75 (99.67 – 99.8)

Roughness Winter North 3 -0.76 (-1.51 – -0.02) 99.82 (99.76 – 99.86)

Roughness Winter South 1 -0.24 (-0.33 – -0.14) NA

TPI Spring Combined 6 0.28 (0.11 – 0.44) 98.44 (97.75 – 98.92)

TPI Spring North 4 0.33 (0.14 – 0.52) 98.31 (97.27 – 98.96)

TPI Spring South 2 0.18 (-0.21 – 0.56) 99.28 (98.66 – 99.61)

TPI Summer Combined 8 -0.07 (-0.29 – 0.15) 98.95 (98.62 – 99.2)

TPI Summer North 4 0.29 (-0.08 – 0.66) 97.51 (95.18 – 98.72)

TPI Summer South 5 -0.28 (-0.48 – -0.09) 99.03 (98.62 – 99.31)

TPI Winter Combined 6 0.1 (-0.04 – 0.24) 97.01 (95.31 – 98.09)

TPI Winter North 2 -0.03 (-0.22 – 0.16) 96.1 (89.06 – 98.61)

TPI Winter South 4 0.17 (-0.02 – 0.36) 96.75 (94.12 – 98.2)

**Appendix S1.** Examples of Program R script that was used to estimate resource selection functions, map habitat selection indices, map management categories, and conduct meta-analysis to evaluate spatiotemporal variation in of habitat selection patterns for greater sage-grouse (*Centrocercus urophasianus*) in Nevada and northeastern California.

#Program R Script

###########################################################################

###########################################################################

#Example of resource selection model development (three-step proces) for each season and #subregion across Nevada and northeastern California for sage-grouse. Not all variables used in #the modeling are shown below.

library(lme4)

library(AICcmodavg)

###########################

##Step one - EXAMPLE single variable model reduction - carry forward spatial lowest AIC #from each landscape model set, provided <2 AIC from null model

###########################

null<-glmer(present~1+(1|BirdID)+(1|Year),data, weights=weight, family=binomial)

AIC(null)

#Big sagebrush model

pbs1451<-glmer(present~pbs1451+(1|BirdID)+(1|Year),data, weights=weight, family=binomial)

pbs167<-glmer(present~pbs167+(1|BirdID)+(1|Year),data, weights=weight, family=binomial)

pbs439<-glmer(present~pbs439+(1|BirdID)+(1|Year),data, weights=weight, family=binomial)

AIC(pbs1451)

AIC(pbs167)

AIC(pbs439)

#Annual grass model

ag1451<-glmer(present~ag1451+(1|BirdID)+(1|Year),data, weights=weight, family=binomial)

ag167<-glmer(present~ag167+(1|BirdID)+(1|Year),data, weights=weight, family=binomial)

ag439<-glmer(present~ag439+(1|BirdID)+(1|Year),data, weights=weight, family=binomial)

AIC(ag1451)

AIC(ag167)

AIC(ag439)

#Topography models

elevkm<-glmer(present~elevkm+(1|BirdID)+(1|Year),data,  weights= weight, family=binomial)

roughnorm<-glmer(present~roughnorm+(1|BirdID)+(1|Year),data,  weights= weight, family=binomial)

tpi2010<-glmer(present~tpi2010+(1|BirdID)+(1|Year),data,  weights= weight, family=binomial)

tpi510<-glmer(present~tpi510+(1|BirdID)+(1|Year),data,  weights= weight, family=binomial)

AIC(elevkm)

AIC(roughnorm)

AIC(tpi2010)

AIC(tpi510)

# Distance model example

distagr<-glmer(present~distagr+(1|BirdID)+(1|Year),data,  weights= weight, family=binomial)

distagr_exp<-glmer(present~distagr_exp+(1|BirdID)+(1|Year),data,  weights= weight, family=binomial)

AIC(distagr)

AIC(distagr_exp)

###########################

##Step Two - EXAMPLE - correlations between variables from step one. All possible 2-variable combinations examined. If correlated variables (R>0.65), remove variable with greater AIC from step one.

###########################

c1<-cor(data$pbs1451,data$elevkm) #found correlation >0.65. removed elevkm from final model set.

c2  <-  cor(data$pbs1451, data$ag439)

c3  <-  cor(data$pbs1451, data$distagr_exp)

c4  <-  cor(data$elevkm,  data$ag439)

c5  <-  cor(data$elevkm,  data$distagr_exp)

c6  <-  cor(data$ag439,   data$distagr_exp)

###########################

##Step Three - EXAMPLE - Examine all possible 2-variable combinations of variables carried forward from step two. Model Average parameter estimates from entire model set.

###########################

model1<-glmer(present~pbs1451+ag439+(1|BirdID)+(1|Year), data,  weights= weight, family=binomial)

model2  <-glmer(present~pbs1451+distagr_exp+(1|BirdID)+(1|Year), data,  weights= weight, family=binomial)

model3  <-glmer(present~ag439+distagr_exp+(1|BirdID)+(1|Year), data,  weights= weight, family=binomial)

modelset=c( model1, model2,model3 )

modelnames=c( "model1", "model2", "model3" )

print(aictab(cand.set =modelset, modnames =modelnames),digits = 4)

###########################################################################

###########################################################################

Example to create habitat selection index maps from greater sage-grouse (*Centrocercus urophasianus*) resource selection function models for each season and subregion across Nevada and northeastern California.

#########

#load libraries

library(raster)

library(rgdal)

library(raster)

############

## Step One: Create RSF maps for each site and season using final parameter coefficients from RSF modeling

############

#Buffalo Skedattle

######

##spring

# load distance rasters - create km rasters from m rasters

rast.distagr_km <- raster("linear_ag.img")/1000

rast.distperen_km <- raster("linear_perennialstream.img")/1000

rast.distspring_km<- raster("linear_spring.img")/1000

rast.distwtrbdy_km <- raster("linear_waterbody.img")/1000

### Create exp. dist rasters - mean of used points to create exp. dist rasters

#Average of distagr = 3.610333183

#Average of distperen = 5.446424627

#Average of distspring = 3.3683065

rast.distagr_exp<- exp(-1*rast.distagr_km/3.610333183)

rast.distperen_exp<- exp(-1*rast.distperen_km/5.446424627)

rast.distspring_exp<-exp(-1*rast.distspring_km/3.3683065)

#topo rasters

rast.elev_km <- raster("elevation.img")/1000

rast.tpi510 <- raster("tpi510.img")

#land cover rasters - turn percentages into ratios

rast.bare439<-raster("bare439.img")/100

rast.bs1451    <-raster("bigsage1451.img")/100

rast.her1451   <-raster("herb1451.img")/100

rast.nss439    <-raster("nonsage439.img")/100

rast.for1451   <-raster("forest1451.img")/100

rast.lcvar439  <-raster("lcvar439.img")

rast.rip1451   <-raster("riparian1451.img")/100

rast.wm1451   <-raster("wetmeadow1451.img")/100

##calculate rsf and hsi for buff sked - spring

rsf.buffsked_spring<-exp((rast.distagr_exp *-0.6956) + (rast.elev_km * -0.8604) + (rast.tpi510 * 0.0087) + (rast.distperen_exp * -1.1059) + (rast.distspring_exp * -0.7706)  + (rast.distwtrbdy_km * -0.2374) + (rast.bare439 * 0.3776) + (rast.bs1451 * -13.2588) + (rast.her1451 * 8.5743) + (rast.nss439 * -6.5325) + (rast.for1451 * -25.965) + (rast.lcvar439 * -0.4821) + (rast.rip1451 * -57.7197) + (rast.wm1451 * -16.0506))

 writeRaster(rsf.buffsked_spring,"rsf.buffsked_spring.img", overwrite=TRUE)

hsi.buffsked_spring<- rsf.buffsked_spring / (1 + rsf.buffsked_spring)

writeRaster(hsi.buffsked_spring,"hsi.buffsked_spring.img", overwrite=TRUE)

##summer

#distance rasters - create km rasters from m rasters

rast.distperen_km <- raster("linear_perennialstream.img")/1000

rast.distspring_km<- raster("linear_spring.img")/1000

rast.distwtrbdy_km <- raster("linear_waterbody.img")/1000

### Create exp. dist rasters - mean of used points to create exp. dist rasters

#Average of distperen = 6.075319576

#Average of distspring = 4.483067596

rast.distperen_exp<- exp(-1*rast.distperen_km/6.075319576)

rast.distspring_exp<-exp(-1*rast.distspring_km/4.483067596)

#topo rasters Normalize roughness with max of original Rough layer = 9.91894

rast.roughnorm<-raster("roughnorm_cl.img")

rast.tpi510    <- raster("tpi510.img")

#land cover rasters - turn percentages into ratios

rast.bare1451   <-raster("bare1451.img")/100

rast.bs1451    <-raster("bigsage1451.img")/100

rast.her439   <-raster("herb439.img")/100

rast.osage1451<-raster("osage1451.img")/100

rast.for1451   <-raster("forest1451.img")/100

rast.lcvar439  <-raster("lcvar439.img")

rast.rip1451   <-raster("riparian1451.img")/100

rast.wm1451   <-raster("wetmeadow1451.img")/100

##calculate rsf and hsi for buff sked - summer

rsf.buffsked_summer<-exp((rast.bare1451 * -4.2531) + (rast.bs1451 * -10.9696) + (rast.her439 * 9.9763) + (rast.osage1451 * -8.1225) + (rast.for1451  * -4.459) + (rast.lcvar439  * -0.1949) + (rast.rip1451 * -16.8435) + (rast.wm1451 * -14.9554) + (rast.roughnorm  * -17.9131) + (rast.tpi510 * 0.0054) + (rast.distspring_exp * -2.7019) + (rast.distperen_exp * -1.4645) +

(rast.distwtrbdy_km  * -0.2923))

hsi.buffsked_summer<- rsf.buffsked_summer / (1 + rsf.buffsked_summer)

writeRaster(rsf.buffsked_summer,"rsf.buffsked_summer.img", overwrite=TRUE)

writeRaster(hsi.buffsked_summer,"hsi.buffsked_summer.img", overwrite=TRUE)

##Winter

#land cover rasters - turn percentages into ratios

rast.ag1451   <-raster("ag1451.img")/100

rast.agr1451   <-raster("agr1451.img")/100

rast.lcvar439  <-raster("lcvar439.img")

rast.bare167   <-raster("bare167.img")/100

rast.bs1451    <-raster("bigsage1451.img")/100

rast.her167   <-raster("herb167.img")/100

rast.nss1451   <-raster("nonsage1451.img")/100

rast.osage1451<-raster("osage1451.img")/100

#topo rasters

rast.elev_km     <- raster("elevation.img")/1000

rast.tpi510  <- raster("tpi510.img")

rast.roughnorm<-raster("roughnorm_cl.img")

#distance rasters

rast.distperen_km <- raster("linear_perennialstream.img")/1000

rast.distspring_km<- raster("linear_spring.img")/1000

### Create exp. dist rasters - mean of used points to create exp. dist rasters

#Average of distperen = 5.772723053

#Average of distspring = 3.839444534

rast.distperen_exp<- exp(-1*rast.distperen_km/5.772723053)

rast.distspring_exp<-exp(-1*rast.distspring_km/3.839444534) #Buffalo Skedattle

rsf.buffsked_winter<-exp((rast.ag1451 * 11.3656) + (rast.agr1451 * 7.9824) + (rast.lcvar439 * -0.3391) + (rast.bare167 *  0.0199) + (rast.bs1451 * -0.2145) + (rast.her167  * 0.0116) + (rast.nss1451 * -0.2452) + (rast.osage1451 * -0.1235) + (rast.roughnorm * -8.4857) + (rast.elev_km * 0.6178) + (rast.tpi510 * 0.0078) + (rast.distspring_exp * -2.6356) + (rast.distperen_exp * -1.6876))

hsi.buffsked_winter<-rsf.buffsked_winter/(1+rsf.buffsked_winter)

writeRaster(rsf.buffsked_winter,"rsf.buffsked_winter.img", overwrite=TRUE)

writeRaster(hsi.buffsked_winter,"hsi.buffsked_winter.img", overwrite=TRUE)

#####

#Cortez

#####

#spring

#distance rasters - create km rasters from m rasters

rast.distagr_km<-raster("linear_ag.img")/1000

rast.distinter_km<- raster("linear_intermittentstream.img")/1000

rast.distwtrbdy_km <- raster("linear_waterbody.img")/1000

rast.distspring_km<- raster("linear_spring.img")/1000

rast.distwm_km<- raster("linear_wetmeadow.img")/1000

### Create exp. dist rasters - mean of used points to create exp. dist rasters

#Average of distagr = 3.506055121

#Average of distwaterbody = 5.217547562

#Average of distwm = 9.775658301

rast.distagr_exp<- exp(-1*rast.distagr_km/3.506055121)

rast.distwtrbdy_exp<- exp(-1*rast.distwtrbdy_km/5.217547562)

rast.distwm_exp<-exp(-1*rast.distwm_km/9.775658301)

#topo rasters

rast.elev_km<- raster("elevation.img")/1000

rast.tpi510 <- raster("tpi510.img")

rast.roughnorm<-raster("roughnorm_cl.img")

#land cover rasters - turn percentages into ratios

rast.ag167   <-raster("ag167.img")/100

rast.lcvar439  <-raster("lcvar439.img")

rast.rip1451   <-raster("riparian1451.img")/100

rast.pjpct167   <-raster("pj167.img")/100

rast.bare167   <-raster("bare167.img")/100

rast.bs1451    <-raster("bigsage1451.img")/100

rast.nss1451    <-raster("nonsage1451.img")/100

rast.os1451   <-raster("osage1451.img")/100

rast.sagehgt1451m<-raster("sagehgt1451.img")/100

##calculate rsf and hsi for buff sked - spring

rsf.cortez_spring<-exp((rast.ag167* -0.9083) + (rast.lcvar439 * -0.2004) + (rast.rip1451 * -39.3324) + (rast.pjpct167 * -6.1449) + (rast.bare167 * 2.3811) + (rast.bs1451  * 1.1052) +  (rast.nss1451 * 13.5217) + (rast.os1451 * 59.0112) + (rast.distagr_exp * -0.8255) + (rast.tpi510 * 0.0131) + (rast.elev_km * 0.5875) + (rast.roughnorm * -1.1256) + (rast.distinter_km * -0.4026) + (rast.distwtrbdy_exp * 0.4303) + (rast.distspring_km * -0.0315) + (rast.distwm_exp * 0.7172) + (rast.sagehgt1451m * -2.9601))

hsi.cortez_spring<- rsf.cortez_spring / (1 + rsf.cortez_spring)

writeRaster(rsf.cortez_spring,"rsf.cortez_spring.img", overwrite=TRUE)

writeRaster(hsi.cortez_spring,"hsi.cortez_spring.img", overwrite=TRUE)

##summer

#distance rasters - create km rasters from m rasters

rast.distagr_km<-raster("linear_ag.img")/1000

rast.distperen_km<- raster("linear_perennialstream.img")/1000

rast.distwtrbdy_km <- raster("linear_waterbody.img")/1000

rast.distspring_km<- raster("linear_spring.img")/1000

rast.distwm_km<- raster("linear_wetmeadow.img")/1000

### Create exp. dist rasters - mean of used points to create exp. dist rasters

#Average of distspring = 1.183025408

#Average of distperen = 3.750692185

#Average of distwm = 10.52131171

rast.distspring_exp<- exp(-1*rast.distspring_km/1.183025408)

rast.distperen_exp<- exp(-1*rast.distperen_km/3.750692185)

rast.distwm_exp<-exp(-1*rast.distwm_km/10.52131171)

#topo rasters

rast.elev_km<- raster("elevation.img")/1000

rast.tpi510<- raster("tpi510.img")

rast.roughnorm<-raster("roughnorm_cl.img")

#land cover rasters - turn percentages into ratios

rast.ag1451   <-raster("ag1451.img")/100

rast.forest439  <-raster("forest439.img")/100

rast.lcvar1451  <-raster("lcvar1451.img")

rast.rip1451   <-raster("riparian1451.img")/100

rast.pjpct439   <-raster("pj439.img")/100

rast.bare1451  <-raster("bare1451.img")/100

rast.bs1451    <-raster("bigsage1451.img")/100

rast.herb1451    <-raster("herb1451.img")/100

rast.nss439    <-raster("nonsage439.img")/100

rast.os1451   <-raster("osage1451.img")/100

rast.sagehgt167m<-raster("sagehgt167.img")/100

rsf.cortez_summer<-Exp((rast.ag1451 * 5.7187) + (rast.forest439  * -57.1196) + (rast.lcvar1451 * 0.4148) + (rast.rip1451 * -78.3209) + (rast.pjpct439 * -10.3801) + (rast.bare1451 * -0.674) + (rast.bs1451 * 19.9759) + (rast.herb1451 * 19.3627) + (rast.nss439 * 21.3561) + (rast.os1451 * 78.9271) + (rast.sagehgt167m * 0.4794) + (rast.distagr_km * -0.2138) + (rast.elev_km * 5.762) + (rast.roughnorm * 1.3818) + (rast.tpi510 * -0.0049) + (rast.distspring_exp * 4.8615) + (rast.distperen_exp * 2.4569) + (rast.distwtrbdy_km * -0.21) + (rast.distwm_exp * -2.158))

hsi.cortez_summer<- rsf.cortez_summer / (1 + rsf.cortez_summer)

writeRaster(rsf.cortez_summer,"rsf.cortez_summer.img", overwrite=TRUE)

writeRaster(hsi.cortez_summer,"hsi.cortez_summer.img", overwrite=TRUE)

##Winter

#distance rasters - create km rasters from m rasters

rast.distperen_km<- raster("linear_perennialstream.img")/1000

rast.distwtrbdy_km <- raster("linear_waterbody.img")/1000

rast.distspring_km<- raster("linear_spring.img")/1000

### Create exp. dist rasters - mean of used points to create exp. dist rasters

#Average of distspring = 2.613103104

rast.distspring_exp<- exp(-1*rast.distspring_km/2.613103104)

#topo rasters

rast.elev_km <- raster("elevation.img")/1000

rast.tpi510 <- raster("tpi510.img")

rast.roughnorm<-raster("roughnorm_cl.img")

#land cover rasters - turn percentages into ratios

rast.bare167  <-raster("bare167.img")/100

rast.bs1451    <-raster("bigsage1451.img")/100

rast.herb1451    <-raster("herb1451.img")/100

rast.nss1451    <-raster("nonsage1451.img")/100

rast.os439  <-raster("osage439.img")/100

rast.ag1451   <-raster("ag1451.img")/100

rast.agr439   <-raster("agr439.img")/100

rast.lcvar167  <-raster("lcvar167.img")

rast.rip1451   <-raster("riparian1451.img")/100

rsf.cortez_winter<-exp((rast.bare167 * 3.1364) + (rast.bs1451 * 3.2359) + (rast.herb1451  * 2.967) + (rast.nss1451 * 14.7211) + (rast.os439 * 36.6994) + (rast.ag1451 * .8227) +                          (rast.agr439  * -6.4255) + (rast.lcvar167 * -.1862) + (rast.rip1451 * -22.9425) + (rast.elev_km * 1.2408) + (rast.roughnorm * 1.1427) + (tpi510.img *0.0042) + (rast.distperen_km* -.1025) + (rast.distspring_exp * .7594) + (rast.distwtrbdy_km  * -.1306))

hsi.cortez_winter<- rsf.cortez_winter / (1 + rsf.cortez_winter)

writeRaster(rsf.cortez_winter,"rsf.cortez_winter.img", overwrite=TRUE)

writeRaster(hsi.cortez_winter,"hsi.cortez_winter.img", overwrite=TRUE)

#####

#Gollaher

#####

#spring

#distance rasters - create km rasters from m rasters

rast.distagr_km<-raster("linear_ag.img")/1000

rast.distinter_km<- raster("linear_intermittentstream.img")/1000

#topo rasters

rast.tpi510    <- raster("tpi510.img")

rast.roughnorm<-raster("roughnorm_cl.img")

#land cover rasters - turn percentages into ratios

rast.herb1451    <-raster("herb1451.img")/100

rast.nss1451    <-raster("nonsage1451.img")/100

rast.os1451   <-raster("osage1451.img")/100

rast.sagehgt1451m<-raster("sagehgt1451.img")/100

rast.edgevar1451  <-raster("edgevar1451.img")

rast.for1451    <-raster("forest1451.img")

rast.lcvar439  <-raster("lcvar439.img")

rast.rip439   <-raster("riparian439.img")/100

rsf.gollaher_spring<-Exp((rast.herb1451 * 3.131) + (rast.nss1451 * -41.2565) + (rast.os1451 * 158.8759) + (rast.sagehgt1451m * -15.0619) + (rast.edgevar1451 * -.3321) +                    (rast.for1451 * -133.946) + (rast.lcvar439 * -.4719) + (rast.rip439 * -19.9487) + (rast.distagr_km * -.5097) + (rast.roughnorm * -3.6512) + (rast.tpi510 * .0105) + (rast.distinter_km * 1.8655))

hsi.gollaher_spring<-rsf.gollaher_spring/(1+rsf.gollaher_spring)

writeRaster(rsf.gollaher_spring,"rsf.gollaher_spring.img", overwrite=TRUE)

writeRaster(hsi.gollaher_spring,"hsi.gollaher_spring.img", overwrite=TRUE)

#summer

#distance rasters - create km rasters from m rasters

rast.distagr_km<-raster("linear_ag.img")/1000

rast.distinter_km<- raster("linear_intermittentstream.img")/1000

rast.distspring_km<- raster("linear_spring.img")/1000

rast.distwtrbdy_km<- raster("linear_waterbody.img")/1000

### Create exp. dist rasters - mean of used points to create exp. dist rasters

#Average of distinter = 0.298795635

rast.distinter_exp<- exp(-1*rast.distinter_km/0.298795635)

#topo rasters

rast.tpi510    <- raster("tpi510.img")

rast.roughnorm<-raster("roughnorm_cl.img")

#land cover rasters - turn percentages into ratios

rast.herb1451    <-raster("herb1451.img")/100

rast.nss1451    <-raster("nonsage1451.img")/100

rast.os1451   <-raster("osage1451.img")/100

rast.sagehgt439m<-raster("sagehgt439.img")/100

rast.edgevar1451  <-raster("edgevar1451.img")

rast.for1451    <-raster("forest1451.img")/100

rast.lcvar1451  <-raster("lcvar1451.img")

rast.rip1451   <-raster("riparian1451.img")/100

rsf.gollaher_summer<-Exp((rast.herb1451 * 7.3722) + (rast.nss1451  * -12.9658) + (rast.os1451 * 39.5682) + (rast.sagehgt439m * -2.1414) + (rast.edgevar1451 * -.084) + (rast.for1451  * -6.9031) + (rast.lcvar1451 * .0569) + (rast.rip1451  * -11.1401) + (rast.distagr_km * -.3375) + (rast.roughnorm * -5.5155) + (rast.tpi510 * .0049) + (rast.distspring_km * -.5311) +                           (rast.distwtrbdy_km * -.391) + (rast.distinter_exp * -.9123))

hsi.gollaher_summer<-rsf.gollaher_summer/(1+rsf.gollaher_summer)

writeRaster(rsf.gollaher_summer,"rsf.gollaher_summer.img", overwrite=TRUE)

writeRaster(hsi.gollaher_summer,"hsi.gollaher_summer.img", overwrite=TRUE)

#winter

#distance rasters - create km rasters from m rasters

rast.distinter_km<- raster("linear_intermittentstream.img")/1000

rast.distwtrbdy_km<- raster("linear_waterbody.img")/1000

### Create exp. dist rasters - mean of used points to create exp. dist rasters

#Average of distinter = 0.595813988

rast.distinter_exp<- exp(-1*rast.distinter_km/0.595813988)

#landcover rasters

rast.herb439    <-raster("herb439.img")/100

rast.nss1451    <-raster("nonsage1451.img")/100

rast.os1451   <-raster("osage1451.img")/100

rast.sagehgt1451m<-raster("sagehgt1451.img")/100

rast.for1451    <-raster("forest1451.img")/100

rast.lcvar167  <-raster("lcvar167.img")

rast.rip439   <-raster("riparian439.img")/100

#topo rasters

rast.tpi2010    <- raster("tpi2010.img")

rast.roughnorm<-raster("roughnorm_cl.img")

rsf.gollaher_winter<-exp((rast.herb439 * -3.1732) + (rast.nss1451 * -16.8462) + (rast.os1451 * 144.069) + (rast.sagehgt1451m * -3.1802) + (rast.for1451  * -10.8017) + (rast.lcvar167 * -0.2083) + (rast.rip439 * -5.6413) + (rast.roughnorm * -2.775) + (rast.tpi2010 * 0.008) + (rast.distinter_exp * -2.6689) + (rast.distwtrbdy_km * -.4726))

hsi.gollaher_winter<-rsf.gollaher_winter/(1+rsf.gollaher_winter)

writeRaster(rsf.gollaher_winter,"rsf.gollaher_winter.img", overwrite=TRUE)

writeRaster(hsi.gollaher_winter,"hsi.gollaher_winter.img", overwrite=TRUE)

#####

#Lincoln

#####

rast.distagr_km<-raster("linear_ag.img")/1000

rast.distwtrbdy_km<- raster("linear_waterbody.img")/1000

rast.distwm_km<- raster("linear_wetmeadow.img")/1000

rast.distperen_km<- raster("linear_perennialstream.img")/1000

rast.distspring_km<- raster("linear_spring.img")/1000

### Create exp. dist rasters - mean of used points to create exp. dist rasters

#Average of distwm = 0.298795635

rast.distwm_exp<- exp(-1*rast.distwm_km/0.298795635)

#topo rasters

rast.roughnorm<-raster("roughnorm_cl.img")

#land cover rasters- convert from percent to ratio

rast.bs1451<-raster("bigsage1451.img")/100

rast.herb1451<-raster("herb1451.img")/100

rast.nss439<-raster("nonsage439.img")/100

rast.for167<-raster("forest167.img")/100

rast.lcvar1451<-raster("lcvar1451.img")

rast.rip167<-raster("riparian167.img")/100

rast.pj439<-raster("pj439.img")/100

rsf.lincoln_spring<-exp((rast.bs1451 * 15.4287) + (rast.herb1451 * 18.7421) + (rast.nss439 * -14.682) + (rast.for167 * -17.6236) + (rast.lcvar1451 * 0.347) + (rast.rip167 * 2.1149) + (rast.pj439 * -13.4142) + (rast.distagr_km * -0.5909) + (rast.roughnorm * -9.0456) + (rast.distwtrbdy_km * -0.5274) + (rast.distwm_exp * 3.3473) + (rast.distperen_km * -0.3113) + (rast.distspring_km * -0.4378))

hsi.lincoln_spring<-rsf.lincoln_spring/(1+rsf.lincoln_spring)

writeRaster(rsf.lincoln_spring,"rsf.lincoln_spring.img", overwrite=TRUE)

writeRaster(hsi.lincoln_spring,"hsi.lincoln_spring.img", overwrite=TRUE)

#summer

rast.distagr_km<-raster("linear_ag.img")/1000

rast.distwtrbdy_km<- raster("linear_waterbody.img")/1000

rast.distperen_km<- raster("linear_perennialstream.img")/1000

rast.distspring_km<- raster("linear_spring.img")/1000

#land cover rasters- convert from percent to ratio

rast.edgevar439<-raster("edgevar439.img")

rast.for167<-raster("forest167.img")/100

rast.lcvar1451<-raster("lcvar1451.img")

rast.rip1451<-raster("riparian1451.img")/100

rast.pj439<-raster("pj439.img")/100

rast.bs1451<-raster("bigsage1451.img")/100

rast.herb1451<-raster("herb1451.img")/100

rast.nss1451<-raster("nonsage1451.img")/100

rast.sagehgt439m<-raster("sagehgt439.img")/100

rsf.lincoln_summer<-exp((rast.edgevar439 * 0.2438) + (rast.for167 * -1.2348) + (rast.lcvar1451 * 0.6374) + (rast.rip1451 * 9.2975) + (rast.pj439 * -15.0656) + (rast.bs1451 * 21.015) + (rast.herb1451 * 42.85) + (rast.nss1451 * 19.2881) + (rast.sagehgt439m * 2.1198) + (rast.distagr_km * -1.0134) + (rast.distwtrbdy_km * -0.8744) + (rast.distspring_km * -0.6496) + (rast.distperen_km * -0.3463))

hsi.lincoln_summer<-rsf.lincoln.summer/(1+rsf.lincoln.summer)

writeRaster(rsf.lincoln_summer,"rsf.lincoln_summer.img", overwrite=TRUE)

writeRaster(hsi.lincoln_summer,"hsi.lincoln_summer.img", overwrite=TRUE)

#winter

rast.distagr_km<-raster("linear_ag.img")/1000

rast.distwtrbdy_km<- raster("linear_waterbody.img")/1000

rast.distwm_km<- raster("linear_wetmeadow.img")/1000

rast.distspring_km<- raster("linear_spring.img")/1000

rast.distallstr_km<- raster("linear_anystream.img")/1000

### Create exp. dist rasters - mean of used points to create exp. dist rasters

#Average of distspring = 3.627861409

rast.distspring_exp<- exp(-1*rast.distspring_km/3.627861409)

#topo rasters

rast.tpi510<-raster("tpi510.img")

#land cover rasters- convert from percent to ratio

rast.for1451<-raster("forest1451.img")/100

rast.lcvar167<-raster("lcvar167.img")

rast.rip439<-raster("riparian439.img")/100

rast.pj1451<-raster("pj1451.img")/100

rast.bs1451<-raster("bigsage1451.img")/100

rast.nss439<-raster("nonsage439.img")/100

rast.os1451<-raster("osage1451.img")/100

rast.sagehgt1451m<-raster("sagehgt1451.img")/100

rsf.lincoln_winter<-exp((rast.for1451 * 1.4308) + (rast.lcvar167 * -0.3865) + (rast.rip439 * -11.1996) + (rast.pj1451 * -15.2103) + (rast.bs1451 * 11.579) + (rast.nss439 * -7.6042) + (rast.os1451 *  90.5096) + (rast.sagehgt1451m * 2.668) + (rast.distagr_km * -0.2427) + (rast.tpi510 * 0.0199) + (rast.distwtrbdy_km * -0.1554) + (rast.distallstr_km * -2.1506) + (rast.distwm_km * -0.0409) + (rast.distspring_exp * 1.5624))

hsi.lincoln_winter<-rsf.lincoln_winter/(1+rsf.lincoln_winter)

writeRaster(rsf.lincoln_winter,"rsf.lincoln_winter.img", overwrite=TRUE)

writeRaster(hsi.lincoln_winter,"hsi.lincoln_winter.img", overwrite=TRUE)

#####

#Midway

######

#spring

#distance rasters - convert to km

rast.distwtrbdy_km<- raster("linear_waterbody.img")/1000

rast.distwm_km<- raster("linear_wetmeadow.img")/1000

rast.distspring_km<- raster("linear_spring.img")/1000

rast.distallstr_km<- raster("linear_anystream.img")/1000

### Create exp. dist rasters - mean of used points to create exp. dist rasters

#Average of distwm = 8.872937107

rast.distwm_exp<- exp(-1*rast.distwm_km/8.872937107)

#topo rasters

rast.tpi2010<-raster("tpi2010.img")

rast.roughnorm<-raster("roughnorm_cl.img")

#land cover rasters- convert from percent to ratio

rast.herb167<-raster("herb167.img")/100

rast.nss167<-raster("nonsage167.img")/100

rast.os439<-raster("osage439.img")/100

rast.sagehgt1451m<-raster("sagehgt1451.img")/100

rast.agr439<-raster("agr439.img")/100

rast.edgevar167<-raster("edgevar167.img")

rast.lcvar1451<-raster("lcvar1451.img")

rast.pj439<-raster("pj439.img")/100

rsf.midway_spring<-exp((rast.herb167 * 15.7568) + (rast.nss167 * 3.9056) + (rast.os439 * 48.2629) + (rast.sagehgt1451m * 4.2971) + (rast.agr439 * 9.5113) + (rast.edgevar167 * 0.2441) + (rast.lcvar1451 * 0.2651) + (rast.pj439 * -29.5628) + (rast.roughnorm * -1.0887) + (rast.tpi2010 * -0.0023) + (rast.distwm_exp * 2.6831) + (rast.distallstr_km * -1.8187) + (rast.distwtrbdy_km * 0.1608) + (rast.distspring_km * -0.1629))

hsi.midway_spring<-rsf.midway_spring/(1+rsf.midway_spring)

writeRaster(rsf.midway_spring,"rsf.midway_spring.img", overwrite=TRUE)

writeRaster(hsi.midway_spring,"hsi.midway_spring.img", overwrite=TRUE)

#summer

#distance rasters - convert to km

rast.distwtrbdy_km<- raster("linear_waterbody.img")/1000

rast.distwm_km<- raster("linear_wetmeadow.img")/1000

rast.distspring_km<- raster("linear_spring.img")/1000

rast.distagr_km<- raster("linear_ag.img")/1000

### Create exp. dist rasters - mean of used points to create exp. dist rasters

#Average of distwtrbdy = 1.786758734

#Average of distspring = 2.940089193

#Average of distagr = 3.28513958

rast.distwtrbdy_exp<- exp(-1*rast.distwtrbdy_km/1.786758734)

rast.distspring_exp<- exp(-1*rast.distspring_km/2.940089193)

rast.distagr_exp<- exp(-1*rast.distagr_km/3.28513958)

#topo rasters

rast.tpi2010<-raster("tpi2010.img")

#land cover rasters- convert from percent to ratio

rast.edgevar1451<-raster("edgevar1451.img")

rast.for167<-raster("forest167.img")/100

rast.lcvar1451<-raster("lcvar1451.img")

rast.pj1451<-raster("pj1451.img")/100

rast.herb1451<-raster("herb1451.img")/100

rast.nss1451<-raster("nonsage1451.img")/100

rast.os439<-raster("osage439.img")/100

rast.sagehgt1451m<-raster("sagehgt1451.img")/100

rsf.midway_summer<-exp((rast.edgevar1451 * 0.2223) + (rast.for167 * -10) + (rast.lcvar1451 * 0.2213) + (rast.pj1451 * -18.524) + (rast.herb1451 * 36.2511) + (rast.nss1451 * 44.8706) + (rast.os439 * 57.7703) + (rast.sagehgt1451m * -2.5609) + (rast.distagr_exp * 1.3584) + (rast.tpi2010 * -0.015) + (rast.distwm_km * -0.5768) + (rast.distwtrbdy_exp * 3.6783) + (rast.distspring_exp * 2.7534))

hsi.midway_summer<-rsf.midway_summer/(1+rsf.midway_summer)

writeRaster(rsf.midway_summer,"rsf.midway_summer.img", overwrite=TRUE)

writeRaster(hsi.midway_summer,"hsi.midway_summer.img", overwrite=TRUE)

#####

#North Swip

#####

#spring

#distance rasters - convert to km

rast.distwm_km<- raster("linear_wetmeadow.img")/1000

rast.distspring_km<- raster("linear_spring.img")/1000

rast.diststream_km<- raster("linear_anystream.img")/1000

### Create exp. dist rasters - mean of used points to create exp. dist rasters

#Average of distspring = 4.259933397

rast.distspring_exp<- exp(-1*rast.distspring_km/4.259933397)

#topo rasters

rast.elevkm<-raster("elevation.img")/1000

#land cover rasters- convert from percent to ratio

rast.ag1451<-raster("ag1451.img")/100

rast.agr1451<-raster("agr1451.img")/100

rast.for1451<-raster("forest1451.img")/100

rast.lcvar167<-raster("lcvar167.img")

rast.rip1451<-raster("riparian1451.img")/100

rast.pj167<-raster("pj167.img")/100

rast.bs1451<-raster("bigsage1451.img")/100

rast.herb1451<-raster("herb1451.img")/100

rast.nss1451<-raster("nonsage1451.img")/100

rast.os1451<-raster("osage1451.img")/100

rsf.northswip_spring<-exp((rast.ag1451 * -93.412) + (rast.agr1451 * 18.2454) + (rast.for1451 * -10.0809) + (rast.lcvar167 * -0.1078) + (rast.rip1451 * -12.7486) + (rast.pj167 * -3.4204) + (rast.elevkm * 0.2516) + (rast.distspring_exp * -0.2053) + (rast.diststream_km * -1.4408) + (rast.distwm_km * 0.0957) + (rast.bs1451 * 13.827) + (rast.herb1451 * 31.4804) + (rast.nss1451 * -11.4745) + (rast.os1451 * 63.9588))

hsi.northswip_spring<-rsf.northswip_spring/(1+rsf.northswip_spring)

writeRaster(rsf.northswip_spring,"rsf.northswip_spring.img", overwrite=TRUE)

writeRaster(hsi.northswip_spring,"hsi.northswip_spring.img", overwrite=TRUE)

#summer

#distance rasters - convert to km

rast.distagr_km<- raster("linear_ag.img")/1000

rast.distspring_km<- raster("linear_spring.img")/1000

rast.distwtrbdy_km<- raster("linear_waterbody.img")/1000

rast.distwm_km<- raster("linear_wetmeadow.img")/1000

### Create exp. dist rasters - mean of used points to create exp. dist rasters

#Average of distspring = 1.46999118

#Average of distwm = 10.29856542

rast.distspring_exp<- exp(-1*rast.distspring_km/1.46999118)

rast.distwm_exp<- exp(-1*rast.distwm_km/10.29856542)

#topo rasters

rast.elevkm<-raster("elevation.img")/1000

rast.tpi2010<-raster("tpi2010.img")

#land cover rasters- convert from percent to ratio

rast.ag167<-raster("ag167.img")/100

rast.edgevar1451<-raster("edgevar1451.img")

rast.for1451<-raster("forest1451.img")/100

rast.lcvar167<-raster("lcvar167.img")

rast.rip167<-raster("riparian167.img")/100

rast.pj167<-raster("pj167.img")/100

rast.bs1451<-raster("bigsage1451.img")/100

rast.herb1451<-raster("herb1451.img")/100

rast.nss1451<-raster("nonsage1451.img")/100

rast.os439<-raster("osage439.img")/100

rast.sagehgt1451<-raster("sagehgt1451.img")/100

rsf.northswip_summer<-exp((rast.ag167 * -39.7606) + (rast.edgevar1451 * 0.1276) + (rast.for1451 * -5.4273) + (rast.lcvar167 * 0.2796) + (rast.rip167 * 6.3242) + (rast.pj167 * -4.5342) + (rast.bs1451 * 12.5717) + (rast.herb1451 * 59.3569) + (rast.nss1451 * 11.532) + (rast.os439 * 14.4314) + (rast.sagehgt1451 * 3.2879) + (rast.distagr_km * 0.086) + (rast.elevkm * 1.7424) + (rast.tpi2010 * -0.0071) + (rast.distspring_exp * 3.3865) + (rast.distwtrbdy_km * -0.0575) + (rast.distwm_exp * -0.974))

hsi.northswip_summer<-rsf.northswip_summer/(1+rsf.northswip_summer)

writeRaster(rsf.northswip_summer,"rsf.northswip_summer.img", overwrite=TRUE)

writeRaster(hsi.northswip_summer,"hsi.northswip_summer.img", overwrite=TRUE)

#winter

#distance rasters - convert to km

rast.distagr_km<- raster("linear_ag.img")/1000

rast.distwtrbdy_km<- raster("linear_waterbody.img")/1000

rast.distwm_km<- raster("linear_wetmeadow.img")/1000

### Create exp. dist rasters - mean of used points to create exp. dist rasters

#Average of distwtrbdy = 7.054993575

#Average of distwm = 17.50578612

rast.distwtrbdy_exp<- exp(-1*rast.distwtrbdy_km/7.054993575)

rast.distwm_exp<- exp(-1*rast.distwm_km/17.50578612)

#topo rasters

rast.tpi2010<-raster("tpi2010.img")

#land cover rasters- convert from percent to ratio

rast.edgevar1451<-raster("edgevar1451.img")

rast.for1451<-raster("forest1451.img")/100

rast.rip1451<-raster("riparian1451.img")/100

rast.bare439<-raster("bare439.img")/100

rast.bs1451<-raster("bigsage1451.img")/100

rast.herb1451<-raster("herb1451.img")/100

rast.nss1451<-raster("nonsage1451.img")/100

rast.os1451<-raster("osage1451.img")/100

rsf.northswip_winter<-exp((rast.edgevar1451 * -0.1665) + (rast.for1451 * -8.1898) + (rast.rip1451 * -158.8993) + (rast.bare439 * 6.6463) + (rast.bs1451 * 4.4969) + (rast.herb1451 * 27.8767) + (rast.nss1451 * -8.3686) + (rast.os1451 * 90.9287) + (rast.distagr_km * -0.2958) + (rast.tpi2010 * -0.0035) + (rast.distwm_exp * -7.6342) + (rast.distwtrbdy_exp * -4.8366))

hsi.northswip_winter<-rsf.northswip_winter/(1+rsf.northswip_winter)

writeRaster(rsf.northswip_winter,"rsf.northswip_winter.img", overwrite=TRUE)

writeRaster(hsi.northswip_winter,"hsi.northswip_winter.img", overwrite=TRUE)

#####

#SouthSwip

#####

#spring

#distance rasters - convert to km

rast.distagr_km<- raster("linear_ag.img")/1000

rast.distinter_km<- raster("linear_intermittentstream.img")/1000

rast.distwtrbdy_km<- raster("linear_waterbody.img")/1000

rast.distspring_km<- raster("linear_spring.img")/1000

### Create exp. dist rasters - mean of used points to create exp. dist rasters

#Average of distagr = 4.086654415

#Average of distinter = 0.161309374

#Average of distspring = 4.564071292

rast.distagr_exp<- exp(-1*rast.distagr_km/4.086654415)

rast.distinter_exp<- exp(-1*rast.distinter_km/0.161309374)

rast.distspring_exp<- exp(-1*rast.distspring_km/4.564071292)

#topo rasters

rast.roughnorm<-raster("roughnorm_cl.img")

#land cover rasters- convert from percent to ratio

rast.edgevar1451<-raster("edgevar1451.img")

rast.lcvar1451<-raster("lcvar1451.img")

rast.rip167<-raster("riparian167.img")/100

rast.pj439<-raster("pj439.img")/100

rast.bs1451<-raster("bigsage1451.img")/100

rast.herb1451<-raster("herb1451.img")/100

rast.nss1451<-raster("nonsage1451.img")/100

rsf.southswip_spring<-exp((rast.edgevar1451 *-0.1797) + (rast.lcvar1451*0.5829) + (rast.rip167*-6.135) + (rast.pj439*-9.6006) + (rast.bs1451*8.1254) + (rast.herb1451*-38.6245) + (rast.nss1451*-7.5055) + (rast.distagr_exp*3.8913) + (rast.roughnorm*-9.9535) + (rast.distinter_exp*1.185) + (rast.distwtrbdy_km*-0.1084) + (rast.distspring_exp*-0.3306))

hsi.southswip_spring<-rsf.southswip_spring/(1+rsf.southswip_spring)

writeRaster(rsf.southswip_spring,"rsf.southswip_spring.img", overwrite=TRUE)

writeRaster(hsi.southswip_spring,"hsi.southswip_spring.img", overwrite=TRUE)

#summer

#distance rasters - convert to km

rast.distagr_km<- raster("linear_ag.img")/1000

rast.distwm_km<- raster("linear_wetmeadow.img")/1000

rast.distspring_km<- raster("linear_spring.img")/1000

rast.distallstr_km<- raster("linear_anystream.img")/1000

rast.distwtrbdy_km<- raster("linear_waterbody.img")/1000

### Create exp. dist rasters - mean of used points to create exp. dist rasters

#Average of distagr = 1.262229234

rast.distagr_exp<- exp(-1*rast.distagr_km/1.262229234)

#topo rasters

rast.elevkm<-raster("elevation.img")/1000

rast.tpi2010<-raster("tpi2010.img")/1000

#land cover rasters- convert from percent to ratio

rast.edgevar439<-raster("edgevar439.img")

rast.rip1451<-raster("riparian1451.img")/100

rast.pj439<-raster("pj439.img")/100

rast.bs1451<-raster("bigsage1451.img")/100

rast.herb1451<-raster("herb1451.img")/100

rsf.southswip_summer<-  exp((rast.edgevar439 *-0.4168) + (rast.rip1451 * -27.4116) + (rast.pj439 * -12.2835) + (rast.bs1451 * 19.8288) + (rast.herb1451 * -26.5077) + (rast.distagr_exp*7.5022) + (rast.elevkm * -1.5521) + (rast.tpi2010 * -0.0094) + (rast.distwm_km * -0.1096) + (rast.distspring_km * -0.1888) + (rast.distallstr_km * -3.2517) + (rast.distwtrbdy_km*-0.3055))

hsi.southswip_summer<-rsf.southswip_summer/(1+rsf.southswip_summer)

writeRaster(rsf.southswip_summer,"rsf.southswip_summer.img", overwrite=TRUE)

writeRaster(hsi.southswip_summer,"hsi.southswip_summer.img", overwrite=TRUE)

#####

#Toiyabe

#####

#spring

#distance rasters- convert to km

rast.distagr_km<- raster("linear_ag.img")/1000

rast.distspring_km<- raster("linear_spring.img")/1000

rast.distwm_km<- raster("linear_wetmeadow.img")/1000

rast.distwtrbdy_km<- raster("linear_waterbody.img")/1000

#land cover rasters- convert from percent to ratio

rast.ag1451<-raster("ag1451.img")/100

rast.edgevar1451<-raster("edgevar1451.img")

rast.rip1451<-raster("riparian1451.img")/100

rast.pj439<-raster("pj439.img")/100

rast.herb1451<-raster("herb1451.img")/100

rast.nss167<-raster("nonsage167.img")/100

rast.osage1451<-raster("osage1451.img")/100

rast.sagehgt1451<-raster("sagehgt1451.img")/100

rsf.toiyabe_spring<-exp((rast.ag1451*5.953) + (rast.edgevar1451*0.1393) + (rast.rip1451*27.1963) + (rast.pj439*-55.5039) + (rast.herb1451 *20.5437) + (rast.nss167 *14.3917) + (rast.osage1451*101.1017) + (rast.sagehgt1451*10.6718) + (rast.distagr_km*-0.2627) + (rast.distspring_km*-0.5512) + (rast.distwm_km*-0.1841) + (rast.distwtrbdy_km*-0.1474))

hsi.toiyabe_spring<-rsf.toiyabe_spring/(1+rsf.toiyabe_spring)

writeRaster(rsf.toiyabe_spring,"rsf.toiyabe_spring.img", overwrite=TRUE)

writeRaster(hsi.toiyabe_spring,"hsi.toiyabe_spring.img", overwrite=TRUE)

#summer

#distance rasters- convert to km

rast.distagr_km<- raster("linear_ag.img")/1000

rast.distperen_km<- raster("linear_perennialstream.img")/1000

rast.distwm_km<- raster("linear_wetmeadow.img")/1000

rast.distwtrbdy_km<- raster("linear_waterbody.img")/1000

#topo rasters

rast.roughnorm<-raster("roughnorm_cl.img")

rast.tpi510<-raster("tpi510.img")

#land cover rasters- convert from percent to ratio

rast.bs1451<-raster("bigsage1451.img")/100

rast.herb1451<-raster("herb1451.img")/100

rast.nss1451<-raster("nonsage1451.img")/100

rast.osage1451<-raster("osage1451.img")/100

rast.ag1451<-raster("ag1451.img")/100

rast.edgevar1451<-raster("edgevar1451.img")

rast.rip1451<-raster("riparian1451.img")/100

rast.pj439<-raster("pj439.img")/100

rsf.toiyabe_summer<-exp((rast.bs1451*28.9438) + (rast.herb1451*32.3815) + (rast.nss1451*-5.6426) + (rast.osage1451*57.8935) + (rast.ag1451*-8.4993) + (rast.edgevar1451*0.4078) + (rast.rip1451*26.0632) + (rast.pj439*-51.8312) + (rast.distagr_km *-0.3417) + (rast.roughnorm*-4.408) + (rast.tpi510*-0.0145) + (rast.distperen_km*-0.3517) + (rast.distwm_km*-0.1154) + (rast.distwtrbdy_km*-0.1027))

hsi.toiyabe_summer<-rsf.toiyabe_summer/(1+rsf.toiyabe_summer)

writeRaster(rsf.toiyabe_summer,"rsf.toiyabe_summer.img", overwrite=TRUE)

writeRaster(hsi.toiyabe_summer,"hsi.toiyabe_summer.img", overwrite=TRUE)

#Winter

#distance rasters- convert to km

rast.distagr_km<- raster("linear_ag.img")/1000

rast.distspring_km<- raster("linear_spring.img")/1000

rast.distwtrbdy_km<- raster("linear_waterbody.img")/1000

rast.distwm_km<- raster("linear_wetmeadow.img")/1000

#topo rasters

rast.tpi510<-raster("tpi510.img")

#land cover rasters- convert from percent to ratio

rast.ag1451<-raster("ag1451.img")/100

rast.edgevar1451<-raster("edgevar1451.img")

rast.pj439<-raster("pj439.img")/100

rast.bs1451<-raster("bigsage1451.img")/100

rast.herb1451<-raster("herb1451.img")/100

rast.nss1451<-raster("nonsage1451.img")/100

rast.osage1451<-raster("osage1451.img")/100

rsf.toiyabe_winter<-exp((rast.ag1451 * -2.0839) + (rast.edgevar1451 * -.2511) + (rast.pj439 * -56.4268) + (rast.bs1451 * -8.0514) + (rast.herb1451 * -6.3113) + (rast.nss1451 * -13.3611) + (rast.osage1451 * 86.0449) + (rast.distagr_km * -0.3368) + (rast.tpi510 * 0.0084) + (rast.distspring_km * -0.7139) + (rast.distwtrbdy_km * -0.3104) + (rast.distwm_km * -0.1173))

hsi.toiyabe_winter<-rsf.toiyabe_winter/(1+rsf.toiyabe_winter)

writeRaster(rsf.toiyabe_winter,"rsf.toiyabe_winter.img", overwrite=TRUE)

writeRaster(hsi.toiyabe_winter,"hsi.toiyabe_winter.img", overwrite=TRUE)

#####

#Tuscarora

#####

#spring

#distance rasters- convert to km

rast.distagr_km<- raster("linear_ag.img")/1000

rast.distwm_km<- raster("linear_wetmeadow.img")/1000

rast.diststr_km<- raster("linear_anystream.img")/1000

rast.distwtrbdy_km<- raster("linear_waterbody.img")/1000

rast.distspring_km<- raster("linear_spring.img")/1000

### Create exp. dist rasters - mean of used points to create exp. dist rasters

#Average of distagr = 1.763895157

#Average of distwtrbdy = 3.626481695

rast.distagr_exp<- exp(-1*rast.distagr_km/1.763895157)

rast.distwtrbdy_exp<- exp(-1*rast.distwtrbdy_km/3.626481695)

#topo rasters

rast.roughnorm<-raster("roughnorm_cl.img")

rast.tpi510<-raster("tpi510.img")

rast.elevkm<-raster("elevation.img")/1000

#land cover rasters- convert from percent to ratio

rast.ag1451<-raster("ag1451.img")/100

rast.edgevar1451<-raster("edgevar1451.img")

rast.for439<-raster("forest439.img")/100

rast.lcvar167<-raster("lcvar167.img")

rast.rip167<-raster("riparian167.img")/100

rast.bare1451<-raster("bare1451.img")/100

rast.herb167<-raster("herb167.img")/100

rast.nss167<-raster("nonsage167.img")/100

rast.osage439<-raster("osage439.img")/100

rsf.tuscarora_spring<-exp((rast.distagr_exp * 0.5726) + (rast.roughnorm * -12.604) + (rast.tpi510 * 0.0136) + (rast.elevkm * -6.6691) + (rast.distwm_km * -0.1894) + (rast.diststr_km * 3.5463) + (rast.distwtrbdy_exp * -2.9042) + (rast.distspring_km * 0.0776) + (rast.ag1451 * -21.8003) + (rast.edgevar1451 * 0.0107) + (rast.for439 * -13.3164) + (rast.lcvar167 * -0.1717) + (rast.rip167 * -3.1823) + (rast.bare1451 * -6.6824) + (rast.herb167 * 7.7715) + (rast.nss167 * -25.4107) + (rast.osage439 * 71.8143))

hsi.tuscarora_spring<-rsf.tuscarora_spring/(1+rsf.tuscarora_spring)

writeRaster(rsf.tuscarora_spring,"rsf.tuscarora_spring.img", overwrite=TRUE)

writeRaster(hsi.tuscarora_spring,"hsi.tuscarora_spring.img", overwrite=TRUE)

#summer

#distance rasters- convert to km

rast.distwm_km<- raster("linear_wetmeadow.img")/1000

rast.distwtrbdy_km<- raster("linear_waterbody.img")/1000

rast.distspring_km<- raster("linear_spring.img")/1000

### Create exp. dist rasters - mean of used points to create exp. dist rasters

#Average of distwtrbdy = 3.189391144

rast.distwtrbdy_exp<- exp(-1*rast.distwtrbdy_km/3.189391144)

#topo rasters

rast.roughnorm<-raster("roughnorm_cl.img")

#land cover rasters- convert from percent to ratio

rast.ag1451<-raster("ag1451.img")/100

rast.agr1451<-raster("agr1451.img")/100

rast.for439<-raster("forest439.img")/100

rast.lcvar439<-raster("lcvar439.img")

rast.rip1451<-raster("riparian1451.img")/100

rast.bare1451<-raster("bare1451.img")/100

rast.herb1451<-raster("herb1451.img")/100

rast.osage1451<-raster("osage1451.img")/100

rsf.tuscarora_summer<-exp((rast.ag1451 * -83.203) + (rast.agr1451 * 6.3963) + (rast.for439 * -22.2337) + (rast.lcvar439 * -0.0359) + (rast.rip1451 * -3.3435) + (rast.bare1451 * -14.9272) + (rast.herb1451 * -4.2117) + (rast.osage1451 * 119.187) + (rast.roughnorm * -13.3922) + (rast.distwm_km * -0.1839) + (rast.distwtrbdy_exp * -0.8677) + (rast.distspring_km * 0.0767))

hsi.tuscarora_summer<-rsf.tuscarora_summer/(1+rsf.tuscarora_summer)

writeRaster(rsf.tuscarora_summer,"rsf.tuscarora_summer.img", overwrite=TRUE)

writeRaster(hsi.tuscarora_summer,"hsi.tuscarora_summer.img", overwrite=TRUE)

#Winter

#distance rasters- convert to km

rast.distagr_km<- raster("linear_ag.img")/1000

rast.distwm_km<- raster("linear_wetmeadow.img")/1000

rast.diststr_km<- raster("linear_anystream.img")/1000

rast.distwtrbdy_km<- raster("linear_waterbody.img")/1000

rast.distspring_km<- raster("linear_spring.img")/1000

### Create exp. dist rasters - mean of used points to create exp. dist rasters

#Average of distwtrbdy = 3.29468186

rast.distwtrbdy_exp<- exp(-1*rast.distwtrbdy_km/3.29468186)

#topo rasters

rast.roughnorm<-raster("roughnorm_cl.img")

rast.elevkm<-raster("elevation.img")/1000

rast.tpi2010<-raster("tpi2010.img")

#land cover rasters- convert from percent to ratio

rast.ag1451<-raster("ag1451.img")/100

rast.for1451<-raster("forest1451.img")/100

rast.lcvar439<-raster("lcvar439.img")

rast.rip439<-raster("riparian439.img")/100

rast.herb1451<-raster("herb1451.img")/100

rast.nss439<-raster("nonsage439.img")/100

rast.osage1451<-raster("osage1451.img")/100

rast.sagehgt1451<-raster("sagehgt1451.img")/100

rsf.tuscarora_winter<-exp((rast.ag1451 * -106.856) + (rast.for1451 * -17.6995) + (rast.lcvar439 * -0.4632) + (rast.rip439 * -10.5088) + (rast.herb1451 * 15.6222) + (rast.nss439  * -35.581) + (rast.osage1451 * 68.8564) + (rast.sagehgt1451 * 13.2043) + (rast.distagr_km * -0.2567) + (rast.roughnorm *  -18.7254) + (rast.elevkm * -3.8991) + (rast.tpi2010 * -0.0033) + (rast.distwm_km * -0.1477) + (rast.diststr_km * 4.1234) + (rast.distspring_km * -0.5061) + (rast.distwtrbdy_exp * -3.2527))

hsi.tuscarora_winter<-rsf.tuscarora_winter/(1+rsf.tuscarora_winter)

writeRaster(rsf.tuscarora_winter,"rsf.tuscarora_winter.img", overwrite=TRUE)

writeRaster(hsi.tuscarora_winter,"hsi.tuscarora_winter.img", overwrite=TRUE)

#####

#Virginia

#####

#spring

#distance rasters- convert to km

rast.distwm_km<- raster("linear_wetmeadow.img")/1000

rast.diststr_km<- raster("linear_anystream.img")/1000

rast.distwtrbdy_km<- raster("linear_waterbody.img")/1000

### Create exp. dist rasters - mean of used points to create exp. dist rasters

#Average of diststr = 0.380337612

rast.diststr_exp<- exp(-1*rast.diststr_km/0.380337612)

#topo rasters

rast.roughnorm<-raster("roughnorm_cl.img")

rast.tpi2010<-raster("tpi2010.img")

#land cover rasters- convert from percent to ratio

rast.ag1451<-raster("ag1451.img")/100

rast.agr1451<-raster("agr1451.img")/100

rast.for1451<-raster("forest1451.img")/100

rast.lcvar167<-raster("lcvar167.img")

rast.bare1451<-raster("bare1451.img")/100

rast.herb1451<-raster("herb1451.img")/100

rast.nss167<-raster("nonsage167.img")/100

rast.osage1451<-raster("osage1451.img")/100

rast.sagehgt1451<-raster("sagehgt1451.img")/100

rsf.virginias_spring<-exp((rast.ag1451 * 10.8782) + (rast.agr1451 * -25.1676) + (rast.for1451 * 55.2536) + (rast.lcvar167 * 0.3239) + (rast.bare1451 * -2.7037) + (rast.herb1451 * 28.318) + (rast.nss167 * 22.2067) + (rast.osage1451 * 23.0726) + (rast.sagehgt1451 * 6.58) + (rast.tpi2010 * 0.011) + (rast.roughnorm * 3.8269) + (rast.distwm_km * -0.3506) + (rast.diststr_exp * -2.4872) + (rast.distwtrbdy_km * 0.132))

hsi.virginias_spring<-rsf.virginias_spring/(1+rsf.virginias_spring)

writeRaster(rsf.virginias_spring,"rsf.virginias_spring.img", overwrite=TRUE)

writeRaster(hsi.virginias_spring,"hsi.virginias_spring.img", overwrite=TRUE)

#summer

#distance rasters- convert to km

rast.distagr_km<- raster("linear_ag.img")/1000

rast.distwm_km<- raster("linear_wetmeadow.img")/1000

rast.distperen_km<- raster("linear_perennialstream.img")/1000

rast.distwtrbdy_km<- raster("linear_waterbody.img")/1000

### Create exp. dist rasters - mean of used points to create exp. dist rasters

#Average of distagr = 5.661047223

rast.distagr_exp<- exp(-1*rast.distagr_km/5.661047223)

#topo rasters

rast.tpi510<-raster("tpi510.img")

rast.roughnorm<-raster("roughnorm_cl.img")

#land cover rasters- convert from percent to ratio

rast.ag1451<-raster("ag1451.img")/100

rast.for1451<-raster("forest1451.img")/100

rast.bare1451<-raster("bare1451.img")/100

rast.herb1451<-raster("herb1451.img")/100

rast.nss1451<-raster("nonsage1451.img")/100

rast.osage167<-raster("osage167.img")/100

rsf.virginias_summer<-exp((rast.ag1451 * 9.4518) + (rast.for1451 * 43.3626) + (rast.bare1451 * 8.6867) + (rast.herb1451 * 56.3768) + (rast.nss1451 * 37.3035) + (rast.osage167 * -22.7872) + (rast.distagr_exp * -6.1824) + (rast.tpi510 * 0.0369) + (rast.roughnorm * -8.77) + (rast.distwm_km * -0.1202) + (rast.distperen_km * -0.2261) + (rast.distwtrbdy_km * -0.6569))

hsi.virginias_summer<-rsf.virginias_summer/(1+rsf.virginias_summer)

writeRaster(rsf.virginias_summer,"rsf.virginias_summer.img", overwrite=TRUE)

writeRaster(hsi.virginias_summer,"hsi.virginias_summer.img", overwrite=TRUE)

#############

## Step Two: Create seasonal rasters by averaging across sites

#############

hsi.spring<- (hsi.buffsked_spring + hsi.cortez_spring + hsi.gollaher_spring + hsi.lincoln_spring + hsi.midway_spring + hsi.northswip_spring + hsi.southswip_spring + hsi.toiyabe_spring + hsi.tuscarora_spring + hsi.virginias_spring)/10

hsi.summer<- (hsi.buffsked_summer + hsi.cortez_summer + hsi.gollaher_summer + hsi.lincoln_summer + hsi.midway_summer + hsi.northswip_summer + hsi.southswip_summer + hsi.toiyabe_summer + hsi.tuscarora_summer + hsi.virginias_summer)/10

hsi.winter<- (hsi.buffsked_winter + hsi.cortez_winter + hsi.gollaher_winter + hsi.lincoln_winter + hsi.northswip_winter + hsi.toiyabe_winter + hsi.tuscarora_winter)/7

writeRaster(hsi.spring,"hsi_spring.img", overwrite=TRUE)

writeRaster(hsi.summer,"hsi_summer.img", overwrite=TRUE)

writeRaster(hsi.winter,"hsi_winter.img", overwrite=TRUE)

############

#Step 3: in ArcMap, clip each seasonal raster by hydrographic boundary shapefile. Relativize #by maximum value for each region. Mosaic back together

############

############

#Step 4: Multiply relativized maps (from ArcMap) to create annual HSI continuous surface map

#############

annual.hsi<-hsi.spring.rmax*hsi.summer.rmax*hsi.winter.rmax

writeRaster(annual.hsi,"annual.hsi.img", overwrite=TRUE)

###########################################################################

###########################################################################

#Program R example script used to run meta-analysis for spring habitat variables. Meta-analyses #were conducted for all seasons, and by hydrographic region.

#########

library(metafor)

####Data - Standardized parameter estimates from RSF models from 27 site/seasons

alldata<-read.csv('metadata_all.csv')

agspring<-rma(yi=ParEst, sei=SE, data=alldata, subset=group=='ag'& season=="spring")

agrspring<-rma(yi=ParEst, sei=SE, data=alldata, subset=group=="agr"& season=="spring")

distagrspring<-rma(yi=ParEst, sei=SE, data=alldata, subset=group=="distagr"& season=="spring")

distagr_expspring<-rma(yi=ParEst, sei=SE, data=alldata, subset=group=="distagr_exp"& season=="spring")

distallstrspring<-rma(yi=ParEst, sei=SE, data=alldata, subset=group=="distallstr"& season=="spring")

distallstr_expspring<-rma(yi=ParEst, sei=SE, data=alldata, subset=group=="distallstr_exp"& season=="spring")

distinterspring<-rma(yi=ParEst, sei=SE, data=alldata, subset=group=="distinter"& season=="spring")

distinter_expspring<-rma(yi=ParEst, sei=SE, data=alldata, subset=group=="distinter_exp"& season=="spring")

distperenspring<-rma(yi=ParEst, sei=SE, data=alldata, subset=group=="distperen"& season=="spring")

distperen_expspring<-rma(yi=ParEst, sei=SE, data=alldata, subset=group=="distperen_exp"& season=="spring")

distspringspring<-rma(yi=ParEst, sei=SE, data=alldata, subset=group=="distspring"& season=="spring")

distspring_expspring<-rma(yi=ParEst, sei=SE, data=alldata, subset=group=="distspring_exp"& season=="spring")

distwmspring<-rma(yi=ParEst, sei=SE, data=alldata, subset=group=="distwm"& season=="spring")

distwm_expspring<-rma(yi=ParEst, sei=SE, data=alldata, subset=group=="distwm_exp"& season=="spring")

distwtrbdyspring<-rma(yi=ParEst, sei=SE, data=alldata, subset=group=="distwtrbdy"& season=="spring")

distwtrbdy_expspring<-rma(yi=ParEst, sei=SE, data=alldata, subset=group=="distwtrbdy_exp" & season=="spring")

edgespring<-rma(yi=ParEst, sei=SE, data=alldata, subset=group=="edge"& season=="spring")

elevspring<-rma(yi=ParEst, sei=SE, data=alldata, subset=group=="elev"& season=="spring")

forspring<-rma(yi=ParEst, sei=SE, data=alldata, subset=group=="for"& season=="spring")

lcvarspring<-rma(yi=ParEst, sei=SE, data=alldata, subset=group=="lcvar"& season=="spring")

pbarspring<-rma(yi=ParEst, sei=SE, data=alldata, subset=group=="pbar"& season=="spring")

pbsspring<-rma(yi=ParEst, sei=SE, data=alldata, subset=group=="pbs"& season=="spring")

pherspring<-rma(yi=ParEst, sei=SE, data=alldata, subset=group=="pher"& season=="spring")

pjspring<-rma(yi=ParEst, sei=SE, data=alldata, subset=group=="pj"& season=="spring")

pnssspring<-rma(yi=ParEst, sei=SE, data=alldata, subset=group=="pnss"& season=="spring")

posspring<-rma(yi=ParEst, sei=SE, data=alldata, subset=group=="pos"& season=="spring")

ripspring<-rma(yi=ParEst, sei=SE, data=alldata, subset=group=="rip"& season=="spring")

roughspring<-rma(yi=ParEst, sei=SE, data=alldata, subset=group=="rough"& season=="spring")

sagehtspring<-rma(yi=ParEst, sei=SE, data=alldata, subset=group=="sageht"& season=="spring")

tpispring<-rma(yi=ParEst, sei=SE, data=alldata, subset=group=="tpi"& season=="spring")

wmspring<-rma(yi=ParEst, sei=SE, data=alldata, subset=group=="wm"& season=="spring")

#############################

##Compile model results

##############################

springresults<-data.frame(

  ParEstspring=c(

    coef(agspring),

    coef(agrspring),

    coef(distagrspring),

    coef(distagr_expspring),

    coef(distallstrspring),

    coef(distallstr_expspring),

    coef(distinterspring),

    coef(distinter_expspring),

    coef(distperenspring),

    coef(distperen_expspring),

    coef(distspringspring),

    coef(distspring_expspring),

    coef(distwmspring),

    coef(distwm_expspring),

    coef(distwtrbdyspring),

    coef(distwtrbdy_expspring),

    coef(edgespring),

    coef(elevspring),

    coef(forspring),

    coef(lcvarspring),

    coef(pbarspring),

    coef(pbsspring),

    coef(pherspring),

    coef(pjspring),

    coef(pnssspring),

    coef(posspring),

    coef(ripspring),

    coef(roughspring),

    coef(sagehtspring),

    coef(tpispring),

    coef(wmspring)),

  stderror=c(agspring$se,

              agrspring$se ,

              distagrspring$se ,

              distagr_expspring$se ,

              distallstrspring$se ,

              distallstr_expspring$se ,

              distinterspring$se ,

              distinter_expspring$se ,

              distperenspring$se ,

              distperen_expspring$se ,

              distspringspring$se ,

              distspring_expspring$se ,

              distwmspring$se ,

              distwm_expspring$se ,

              distwtrbdyspring$se ,

              distwtrbdy_expspring$se ,

              edgespring$se ,

              elevspring$se ,

              forspring$se ,

              lcvarspring$se ,

              pbarspring$se ,

              pbsspring$se ,

              pherspring$se ,

              pjspring$se ,

              pnssspring$se ,

              posspring$se ,

              ripspring$se ,

              roughspring$se ,

              sagehtspring$se ,

              tpispring$se ,

              wmspring$se

              ),

  lcl=c(agspring$ci.lb ,

         agrspring$ci.lb ,

         distagrspring$ci.lb ,

         distagr_expspring$ci.lb ,

         distallstrspring$ci.lb ,

         distallstr_expspring$ci.lb ,

         distinterspring$ci.lb ,

         distinter_expspring$ci.lb ,

         distperenspring$ci.lb ,

         distperen_expspring$ci.lb ,

         distspringspring$ci.lb ,

         distspring_expspring$ci.lb ,

         distwmspring$ci.lb ,

         distwm_expspring$ci.lb ,

         distwtrbdyspring$ci.lb ,

         distwtrbdy_expspring$ci.lb ,

         edgespring$ci.lb ,

         elevspring$ci.lb ,

         forspring$ci.lb ,

         lcvarspring$ci.lb ,

         pbarspring$ci.lb ,

         pbsspring$ci.lb ,

         pherspring$ci.lb ,

         pjspring$ci.lb ,

         pnssspring$ci.lb ,

         posspring$ci.lb ,

         ripspring$ci.lb ,

         roughspring$ci.lb ,

         sagehtspring$ci.lb ,

         tpispring$ci.lb ,

         wmspring$ci.lb

         ),

  ucl=c( agspring$ci.ub ,

         agrspring$ci.ub ,

         distagrspring$ci.ub ,

         distagr_expspring$ci.ub ,

         distallstrspring$ci.ub ,

         distallstr_expspring$ci.ub ,

         distinterspring$ci.ub ,

         distinter_expspring$ci.ub ,

         distperenspring$ci.ub ,

         distperen_expspring$ci.ub ,

         distspringspring$ci.ub ,

         distspring_expspring$ci.ub ,

         distwmspring$ci.ub ,

         distwm_expspring$ci.ub ,

         distwtrbdyspring$ci.ub ,

         distwtrbdy_expspring$ci.ub ,

         edgespring$ci.ub ,

         elevspring$ci.ub ,

         forspring$ci.ub ,

         lcvarspring$ci.ub ,

         pbarspring$ci.ub ,

         pbsspring$ci.ub ,

         pherspring$ci.ub ,

         pjspring$ci.ub ,

         pnssspring$ci.ub ,

         posspring$ci.ub ,

         ripspring$ci.ub ,

         roughspring$ci.ub ,

         sagehtspring$ci.ub ,

         tpispring$ci.ub ,

         wmspring$ci.ub

         ),

   I2=c(agspring$I2,

        agrspring$I2 ,

        distagrspring$I2 ,

        distagr_expspring$I2 ,

        distallstrspring$I2 ,

        distallstr_expspring$I2 ,

        distinterspring$I2 ,

        distinter_expspring$I2 ,

        distperenspring$I2 ,

        distperen_expspring$I2 ,

        distspringspring$I2 ,

        distspring_expspring$I2 ,

        distwmspring$I2 ,

        distwm_expspring$I2 ,

        distwtrbdyspring$I2 ,

        distwtrbdy_expspring$I2 ,

        edgespring$I2 ,

        elevspring$I2 ,

        forspring$I2 ,

        lcvarspring$I2 ,

        pbarspring$I2 ,

        pbsspring$I2 ,

        pherspring$I2 ,

        pjspring$I2 ,

        pnssspring$I2 ,

        posspring$I2 ,

        ripspring$I2 ,

        roughspring$I2 ,

        sagehtspring$I2 ,

        tpispring$I2 ,

        wmspring$I2

        ) )
